# The Role of APOSTART in Switching between Sexuality and Apomixis in *Poa pratensis*

**DOI:** 10.3390/genes11080941

**Published:** 2020-08-14

**Authors:** Gianpiero Marconi, Domenico Aiello, Bryan Kindiger, Loriano Storchi, Alessandro Marrone, Lara Reale, Niccolò Terzaroli, Emidio Albertini

**Affiliations:** 1 Dipartimento di Scienze Agrarie, Alimentari e Ambientali, Università degli Studi di Perugia, Borgo XX Giugno 74, 06121 Perugia, Italy; gianpiero.marconi@unipg.it (G.M.); aiellodomenico@yahoo.it (D.A.); lara.reale@unipg.it (L.R.); niccolo.terzaroli@studenti.unipg.it (N.T.); 2 USDA-ARS, Grazinglands Research Laboratory, 7207 West Cheyenne St., El Reno, OK 73036, USA; bryan.kindiger@ars.usda.gov; 3 Dipartimento di Farmacia, Università G. d’Annunzio, via dei Vestini 31, 66100 Chieti, Italy; loriano@storchi.org (L.S.); alessandro.marrone@unich.it (A.M.); 4 Molecular Discovery Limited, Elstree WD6 3FG, UK

**Keywords:** apomixis, APOSTART, plant reproduction, *Poa pratensis*

## Abstract

The production of seeds without sex is considered the holy grail of plant biology. The transfer of apomixis to various crop species has the potential to transform plant breeding, since it will allow new varieties to retain valuable traits thorough asexual reproduction. Therefore, a greater molecular understanding of apomixis is fundamental. In a previous work we identified a gene, namely APOSTART, that seemed to be involved in this asexual mode of reproduction, which is very common in *Poa pratensis* L., and here we present a detailed work aimed at clarifying its role in apomixis. In situ hybridization showed that PpAPOSTART is expressed in reproductive tissues from pre-meiosis to embryo development. Interestingly, it is expressed early in few nucellar cells of apomictic individuals possibly switching from a somatic to a reproductive cell as in aposporic apomixis. Moreover, out of 13 APOSTART members, we identified one, APOSTART_6, as specifically expressed in flower tissue. APOSTART_6 also exhibited delayed expression in apomictic genotypes when compared with sexual types. Most importantly, the SCAR (Sequence Characterized Amplified Region) derived from the APOSTART_6 sequence completely co-segregated with apomixis.

## 1. Introduction

Angiosperms reproduce primarily through sexual reproduction, however in plants, this requires a series of developmental steps that culminate in the formation of the seed. Meiosis and fertilization as the main signs of sexuality, secure the formation of genetically variable diploid progeny [1]. However, this is not the only way to produce seed. In an asexual mode of reproduction, namely various forms of apomixis [2], successful apomictic events typically silence sexual development within the same ovule, allowing for the generation of unreduced gametes [2]. Apomixis generally causes germline cells to completely avoid meiosis or, by modification of meiosis, the full restoration of the sporophyte chromosome number.

Apomixis is a challenging trait that offers unique opportunities for developing superior, true breeding cultivars irrespective of their level of heterozygosity [3,4,5]. The conversion of a sexual reproducing genotype to one that reproduces by apomixis would make it possible to fix the genotype of a superior plant variety selected for a particular environment or market niche, whereby the clonally reproduced seeds could be continuously and inexpensively produced, independent of pollination or pollinator [6].

The main advantages in having an apomictic genotype are that it reduces cost and breeding time for a new cultivar, avoiding various complications associated with sexual reproduction, such as incompatibility barriers and viral transfer in plants typically propagated vegetatively [7]. Moreover, farmers within the developing world could exploit the advantages of maintaining the genetic integrity of local, high-yielding varieties from generation to generation, removing the tendency of open-pollinated local varieties to lose their genetic advantages through sexual reproduction and outcrossing over time. Utilizing local varieties that are adapted to local mechanized agricultural systems could also lower production costs.

Today, the general consensus regarding apomixis and sexual reproduction indicates these as evolutionarily related processes, sharing many regulatory components. Once apomictic genes initiate embryo development and the initial cell forms and divides, the genes controlling embryo cell formation and patterning are most likely the same as those required for sexual embryo development. These similarities recently have been debated [2,8,9], and a new hypothesis has been proposed that considers apomicts anciently polyphenic with sex. This polyphenic viewpoint suggests that apomixis fails to occur in obligate sexual eukaryotes because genetic or epigenetic modifications have silenced the primitive sexual-apomictic switch and/or disrupted molecular capacities for apomixis [2]. According to the polyphyletic hypothesis, apomixis occurs in plants due to specific mutations [10,11]. If apomixis were well understood and harnessed, it could be utilized to indefinitely propagate superior hybrids or specific genotypes bearing complex gene sets. Until the gene(s) that promote and control apomixis are understood at the molecular level, this trait can only be introgressed into agricultural crops through traditional breeding methods, most of which are slow and laborious. Progeny testing for the selection of apomictic genotypes following each round of backcrossing also slows the introgression of apomixis into a cultivar.

Apomixis occurs in thousands of species across all kingdoms of the Eukaria [9], and this includes several important forage grasses, such as *Poa*, *Brachiaria*, *Cenchrus*/*Pennisetum*, *Panicum*, and *Paspalum*. Among these, we elected to investigate *Poa pratensis* L. Kentucky bluegrass (*P. pratensis*) is a highly polyploid, hardy, persistent, and attractive forage and turf grass adapted to a wide range of soils and climates. Its mode of reproduction is extremely versatile ranging from sexuality to natural obligate apomixis (apospory and parthenogenesis). In *P. pratensis*, apospory involves the development of embryo sacs from somatic cells that differentiate into the nucellus. If unreduced polar nuclei positioned centrally within the embryo sac fuse with a sperm cell released from the pollen tube (pseudogamy), the unreduced egg can develop autonomously through parthenogenesis to form viable apomictic seeds [12]. Moreover, overall results indicate that in this species, apospory and parthenogenesis are controlled by two distinct genetic factors that are genetically uncoupled [12]. Therefore, the possibility to find or obtain recombinant genotypes (aposporic but non-parthenogenetic or vice-versa) could be very useful for investigating the impact of different candidate genes. In a previous work we [13] applied a cDNA-AFLP transcriptional profiling assay to developmental staged inflorescences of *P. pratensis* and isolated 2.248 ESTs, most of which (60%) were specific of floral organs and/or were involved in megasporogenesis and seed development. In particular, one expressed sequence tag (EST) clone showed a high similarity with an EST isolated from a pistil-specific cDNA library of apomictic *Pennisetum ciliare* and with the human *StAR* gene that can cause pseudohermaphroditism. Due to its START domain and putative involvement in apomixis, we named this gene *APOSTART* [14]. In this previous work we described the cloning of two APOSTART members (*APOSTART_1* and *APOSTART_2*). Here, we report the isolation of additional 13 cDNA and six genomic clones for which we determined genomic organization, temporal, and spatial expression and, computationally, infer on the binding of phytosterols at the START domain of their coded proteins. Moreover, we used the sequence of our best candidate (*APOSTART_6*) to search for similar proteins and found 207 proteins belonging to 158 species. Their relationships based on distances in the protein amino acid sequences composition were used to build a phylogenetic tree. The putative involvement of APOSTART in apomixis is also reported and discussed.

## 2. Materials and Methods

### 2.1. Plant Material

Cloning of cDNA and genomic full-length clones: a completely sexual clone (S1/1-7) and two highly apomictic genotypes (RS7-3 and L4) of *Poa pratensis* were used to amplify, clone, and sequence the full-lengths of APOSTART members (Table S1).

SCAR marker test: 48 genotypes from a *P. pratensis* segregating F_1_ population (PG-F1) derived from a cross between S1/1-7 and RS7-3 [15] and their parents; forty-three F_1_ interspecific hybrids from a cross between a sexual *P. arachnifera* (Pa1FM) and a highly apomictic *P. pratensis* genotype, evaluated for their mode of reproduction by progeny tests (Table S1); thirteen genotypes of *P. pratensis* from USA with known reproductive behavior, and one commercially available *P. arachnifera* × *P. pratensis* hybrid (Reveille) (Table S1). These Poa materials were selected for the APO-SCAR marker to trait segregation analysis because the *P. pratensis* germplasm was characterized as a monoecious, obligate apomictic, where no sexuality is expressed. *P. arachnifera*, representing an indigenous species to the Central USA, is dioecious and has a purely sexual form of reproduction. Individuals representing the *P. arachnifera* × *P. pratensis* F_1_ hybrid population utilized in the APO-SCAR analysis had been previously characterized for their expression of either a sexual or apomictic form of reproduction. The F_1_ population provided a segregating sexual/apomictic sample with which to evaluate the utility and validity of the APOSTART_6 marker.

qPCR (quantitative PCR): S1/1-7 (sexual), RS7-3 and L4 (apomictic), together with three progeny plants from the S1/1-7 × RS7 cross (2 apomictic and 1 sexual) and four progeny plants from the S1/1-7 × L4 cross (2 sexual, one apomictic, and one parthenogenetic, but not aposporic) of *P. pratensis* (Table S1). Reproduction mode and chromosome numbers of these plants were described in Raggi et al. [16].

### 2.2. Rapid Amplification of cDNA Ends Analysis

Clone-specific primers (Table S2) were used for performing both 5′-and 3′-rapid amplification of cDNA ends (RACE) to obtain the full-length genes. The SMART RACE cDNA amplification kit (BD Biosciences) was applied to the mRNA poly(A)+ of the stage where the cDNA was scored according to the manufacturer’s instructions. Twelve colonies for each RACE experiment were sequenced and full-length cDNA sequences were reconstructed from RACE fragments using the VECTOR NTI^®^ Suite 8 Contig Express (Invitrogen TM, Carlsbad, CA, USA). Sequences were aligned using VECTOR NTI^®^ Suite 8 AlignX software (Invitrogen TM, Carlsbad, CA, USA). Based on this alignment, specific primers were designed to perform end-to-end PCR on cDNA and genomic DNAs (Table S2).

### 2.3. Cloning of Full-Length Genomic and cDNAs

Specific primers, as reported above, were used for performing end-to-end amplifications of cDNAs and genomic DNAs to obtain the entire transcriptional units. A 0.7 μL aliquot of PCR-derived products was sticky-end ligated into a pCR4-TOPO vector using the TOPO TA cloning kit for sequencing (Invitrogen TM, Carlsbad, CA, USA). The plasmid DNA was purified from 5 mL of an overnight culture on LB medium of *E. coli* using the GenElute plasmid miniprep kit (Sigma-Aldrich, St. Louis, MO, USA). After a first confirmation sequence was performed using M13 forward and reverse primers, a primer walking approach was adopted to sequence the complete clones. Alignment between the full-length cDNAs and genomic clones (Table S3) disclosed the intron/exon structures of the genes (Figure S1).

### 2.4. RNA Isolation and cDNA Synthesis

Florets, harvested at five developmental stages (pre-meiosis, meiosis, post-meiosis, anthesis, and post-anthesis) according to cytohistological investigations [12,14], leaves, and roots of five apomictic, four sexual, and one parthenogenetic genotype of *P. pratensis*, were collected. Nucleic acids were isolated from about 0.1 g of fresh tissue using the GenElute total RNA purification kit (Sigma-Aldrich, St. Louis, MO, USA), according to the manufacturer’s instructions, with some modifications to adapt it to plants. Total RNA was purified from residual genomic DNA by using the DNA-free (Ambion, Austin, TX, USA). Reverse transcription and second-strand synthesis were carried out with 1 µg of total RNA, and the standard procedure was followed [17].

### 2.5. qPCR

All qPCR analyses were performed using an Mx3000P qPCR (Stratagene, La Jolla, CA, USA) system with the SYBR green JumpStart Taq ReadyMix for quantitative PCR (Sigma-Aldrich, St. Louis, MO, USA). Specific primers were designed within the sequences of each allele (Table S2). The PCR fragments were analysed using a dissociation protocol to ensure that each amplicon was a single product. Amplicons were also sequenced to verify the specificities of the targets. The amplification efficiency was calculated from raw data using the LingRegPCR software. All RT-qPCRs were performed using three biological replicates in a final volume of 25 µL containing 5 µL of cDNA template (previously diluted 1:10), 0.2 µM of each primer, and 12.5 µL of 2 × SYBR Green PCR Master Mix (Sigma-Aldrich, St. Louis, MO, USA), according to the manufacturer’s instructions. The following thermal cycling profile was used: 95 °C for 10 min, followed by 50 cycles of 95 °C for 10 s, 57 °C for 15 s, and 72 °C for 15 s. Following cycling, the melting curve was determined in the range 57–95 °C, with a temperature increment of 0.01 °C/sec. Each reaction was run in triplicate (technical replicates). Negative controls included in each run were a reaction conducted in the absence of reverse transcriptase and a reaction with no template (2 µL of nuclease-free water instead of 2 µL of cDNA). For negative controls, no signals were observed (data not shown). Raw C_t_ data from the MX3000P instrument were exported to a data file and analysed using GeneEx Pro software (bioMCC, Freising, Germany). During the pre-processing phase, data were corrected for PCR efficiencies and the three technical repeats were averaged. The selected reference genes, *P. pratensis* β-tubulin [13] subsequently used to normalize C_t_ values [18,19], and quantities were calculated relative to the maximum C_t_ value. Because our interest was in fold changes in gene expression between groups, we ultimately converted quantities to a logarithmic scale using a log base 2 conversion, which also allowed us to test the normal distribution of values.

### 2.6. SCAR Marker Development and Testing

Genomic sequences of APOSTART alleles (Table S3) were aligned using the VECTOR NTI^®^ Suite 8 AlignX software (Invitrogen TM, Carlsbad, CA, USA) to disclose differences in nucleotide composition. Several primer pairs were designed on the basis of genomic differences and tested on the F_1_ population segregating for the mode of reproduction (Table S1). Amplifications of genomic DNAs with primer pairs were done in a final volume of 25 µL containing 1× PCR buffer (Invitrogen TM, Carlsbad, CA, USA), 5 mM dNTPs, 20 pmol of each primer, 25 ng of genomic DNA, and 1 U recombinant Taq DNA polymerase (Invitrogen). PCR was carried out in an initial denaturation step of 94 °C for 5 min, followed by 30 cycles of 94 °C for 30 s, 60 °C for 30 s, 72 °C for 1 min, and a final extension step of 72 °C for 10 min. Amplification products were separated by electrophoresis in 2% agarose gels. The primer pair that co-segregate with apomixis (hereafter named APO-SCAR, Table S2) was then tested also on other genotypes. The APO-SCAR PCR was carried out in an initial denaturation step of 94 °C for 5 min, followed by 40 cycles of 94 °C for 30 s, 59 °C for 30 s, 72 °C for 30 s, and a final extension step of 72 °C for 10 min. In-PCR positive controls were obtained by amplifying a chloroplast sequence using a chloroplast-specific pair of primers (Table S2).

### 2.7. In Situ Hybridization

During inflorescence development, in sexual and apomictic *P. pratensis* genotypes, we distinguished four different stages (pre-meiosis, meiosis, post-meiosis, anthesis, and post-anthesis). At each stage, single spikelets were collected, fixed in ethanol-formaldehyde-acetic acid, embedded in paraffin, and used for ISH experiments. Tissue preparation and hybridization conditions were the same as described by Angenent et al. [20].

Sense and antisense probes were obtained by in vitro transcription using cloned PCR-derived fragments APOSTART as templates. In particular, APOSTART riboprobes were obtained from a single cDNA fragment of 1060 bp, which comprised only a small part of the START and DUF1336 domains and the sequence between the two domains. APOSTART riboprobes did not discriminate between alleles due to the very small differences in sequences.

DIG-UTP sense and antisense riboprobes were synthesized by the T3 and T7 RNA polymerase. Transcripts were partially hydrolyzed by incubation at 60 °C in 0.2 M Na_2_CO_3_/NaHCO_3_ buffer, pH 10.2, for about 35 min. Immunological detection was performed as described by Cañas et al. [21].

### 2.8. Sequence Data Analysis and Phylogenetic Trees

Using *APOSTART_6* cDNA and amino acid sequences as a query, similarities were searched in the National Center for Biotechnology Information (NCBI; www.ncbi.nlm.nih.gov) [22]. In both cases, multiple sequence alignment and phylogenetic tree were obtained using the function ClustalW with default parameters in the Molecular Evolutionary Genetics Analysis Version 7.0 (MEGA7). Protein sequences were first analyzed with MEGA function “Find Best DNA/Protein Models (ML)”, which indicated Jones-Taylor-Thornton as the best method to construct the maximum-likelihood phylogenetic tree [23]. A discrete γ distribution was used to model evolutionary rate differences among sites (5 categories (+Gamma parameter)) with 1000 of Bootstrap as the resampling method [24].

### 2.9. Phosphorylation Site, Secondary Structure Predictions, and Subcellular Localization Prediction

Phosphorylation sites analyses of the 15 APOSTART amino acids were performed with NetPhos 3.1 Server [25]. Secondary structures, solvent accessibility, disordered regions, and trans-membrane helices were predicted using RaptorX servers (http://raptorx.uchicago.edu/) and PredictProtein servers (http://www.predictprotein.org) [26,27,28,29]. Subcellular localization prediction of APOSTART proteins was carried out using in-silico prediction tools, such as: CELLO2GO [30], DeepLoc [31], MultiLoc2 [32], Plant-mPloc [33], and SherLoc2 [34], Yloc [35].

### 2.10. Molecular Modeling

The protein sequences coded by APOSTART_1, APOSTART_6, and APOSTART_8 genes were subjected to the computational workflow depicted in Figure S1. Preliminary, reliable template structures for the initiation of the homology modeling investigations were searched for by using the HHpred web-server toolkits [36]. In particular, we carried out template searches on all available databases. The protein structures with higher scores and reported in the pdb archive were identified as plausible entries to be used as homology modeling templates. The graphical inspection of these structures was then performed by the use of the Maestro software [37] and allowed to identify the pdb coded 1LN1 entry as the most reliable for our purposes (vide infra). Homology modeling calculations were, then, carried out on the protein sequences of APOSTART_1, APOSTART_6, and APOSTART_8 with the 1LN1 protein structure as a template by using the Modeller software [38]. Modeller models 3D structures of proteins and their assemblies by the use of spatial restraints is most frequently used for homology or comparative protein structure modeling. Once provided with an alignment of a sequence to be modeled with a proper template, Modeller calculates a 3D model with all non-hydrogen atoms. The APOSTART_1-1LN1 and APOSTART_6-1LN1 sequence alignments used in the Modeller runs were reported in S2. The resulting 3D models were obtained by optimizing the molecular probability density function (pdf) subjected to only spatial restraints in Cartesian space and by employing the conjugate gradients and molecular dynamics with simulated annealing procedures implemented in the Modeller software.

The 3D structures of APOSTART_1, APOSTART_6, and APOSTART_8 provided at the homology modeling stage were refined by using the Protein Preparation Wizard implemented in Maestro [39]. This step allowed us to gain an all-atom and OPLS force field [40] with consistent protein structures to be processed in the further stages of the workflow. To gain a proper orientation of the binding site protein side chains, the structure of the co-crystallized ligand retrieved from the 1ln1 pdb entry was included in the model during the refinement steps. Optimized structures of APOSTART_1 and APOSTART_6 corresponding to local minima of the potential energy surface (PES) were calculated with the OPLS force field by using the GB/SA method [41] to simulate the water environment and the LBFGS minimization algorithm at a maximum iteration limit of 10,000 steps and a gradient-based convergence threshold of 0.05 kJ mol^−1^ Å^−1^. A further refinement of the obtained protein structures of APOSTART_1 and APOSTART_6 was carried out by the use of the large-scale low-mode (LLMOD) algorithm implemented in MacroModel [42,43]. This procedure allowed sampling the PES by moving along the direction of the low-energy eigenvectors of the hessian matrix to search for lower energy structures, thus corresponding to a “local” conformational search. A maximum of 1000 LLMOD steps along the directions (eigenvectors) associated to the 30 lowest eigenvalues of the hessian matrix is used in the conformational sampling; each sampled structure is filtered according to the Monte Carlo criterium and eventually minimized in the OPLS force field by the use of loose convergence criteria (gradient-based convergence threshold of 0.5 kJ mol^−1^ Å^−1^). Each LLMOD run provided 20 conformations that were further optimized with MacroModel, and the resulting lowest energy structure was eventually selected as the final 3D model of the considered protein (upon removing the 1ln1 co-crystallized ligand).

The 3D structures of stigmasterol, brassicasterol and campesterol were sketched in the Maestro workspace and the corresponding local minimum structures were calculated using the OPLS force field with the TNCG minimization algorithm and a convergence threshold of 0.05 kJ mol^−1^ Å^−1^.

Subsequently, docking calculations were performed by the use of Glide software [44] in two stages. Initially, the grid generation module was employed to delimit the binding site region on APOSTART_1 and APOSTART_6 protein structure and to calculate the molecular interaction fields to be used for the following docking evaluation. The binding site was represented by a box of 28 Å per dimension and a smaller sub-box of 10 Å per dimension to delimit the space in which the center of mass of each ligand can be positioned. The docking SP procedure was then employed to dock flexibly the considered phytosterols in the binding site of APOSTART_1 and APOSTART_6 to obtain 10 poses per ligand.

### 2.11. Consensus Scoring and Molecular Dynamics

The bound complexes of APOSTART_1, APOSTART_6, and APOSTART_8 obtained from the docking calculations underwent a further refinement step consisting of a multiple minimization performed with MacroModel using the same settings as protein structure minimization (see Section 2.10). The 90 structures of bound complexes obtained after the post-docking refinement were processed to estimate the corresponding target-ligand binding energy by using three different methods. First, we performed “Rigid” docking calculations with Glide at the XP level of precision using the GlideScore fitness function [45]. Then, an estimation of the energy for the “snapping” of bound complexes obtained through the single-point energy calculation on complex, free protein, and free ligands with MacroModel were generated. Finally, we estimated the target-ligand affinity by using the FLAP (fingerprints for ligands and proteins) procedure [46] that provides a common reference framework for comparing molecules, using GRID molecular interaction fields (MIFs). The GRID MIFs (i.e., GRID molecular interaction fields) [47], originally developed for structure-based drug design [48], have been applied to a variety of drug discovery areas over the years, such as *pKa* [49] and tautomers modeling [50], scaffold-hopping [51], 3D-QSAR [52], and metabolism prediction [53]. Using the GRID MIFs one can easily obtain information related to non-covalent bonding between the selected probe and the target. The target may either be a small molecule or a protein. Probes, on the other hand, represent different chemical moieties (e.g., OH2 a water molecule, DRY the hydrophobic probe, N1 a neutral lat N-H, O a carbonyl oxygen, etc.) that are located in a 3D grid surrounding the target. At each point of the 3D grid the interaction energy is computed by determining and summing up the Lennard-Jones (ELJ), electrostatic (EEL), hydrogen-bonding (EHB), and entropic (ES) terms. We estimated the target-ligand affinity using the GP value returned by FLAP that is basically a product of the major GRID probes (H, N1, O, and DRY) similarity (i.e., FLAP similarity is a kind of Tanimoto similarity, weighted by energy).

The consensus scoring was obtained by averaging the estimations of Glide, MacroModel, and FLAP. For this purpose, each set of estimations was transformed to assign the same metrics, based on standard deviation units (i.e., they have been normalized), to the three-scoring set. The deviation of each value from the minimum score (penalty) was calculated with:

$$P_{ij}=\frac{S_{ij}-\min\left\{S_{1j},S_{2j}\ldots S_{Nj}\right\}}{\sigma_{j}}$$

where *S_ij_* is the *i*-th scoring value of *j*-th scoring, and *σ_j_* is the standard deviation on the *j*-th set of N scoring values. The *j*-th normalized score is then obtained as the deviation of penalty from its maximum:

$${\bar{S}}_{ij}=\max\;\left\{p_{1j},p_{2j}\ldots p_{Nj}\right\}-p_{ij}$$

The consensus scoring for the *i*-th bound system is eventually expressed by:

$$S_{i}=\;\frac{\sum_{j-i}^{3}\;{\bar{S}}_{ij}}{3}$$

The bound complexes of APOSTART_1, APOSTART_6, and APOSTART_8 with stigmasterol, brassicasterol, and campesterol gaining the highest consensus scoring (two top-scored per bound complex) were further investigated by molecular dynamics (MD) simulations with the Gromacs package [54]. Each bond complex was placed in a cubic box whose dimensions prevent self-interaction and, then, solvated with up to 16,300 water molecules, depending on both ligand and binding mode, at the typical density of water at 298 K and 1.0 atm and by employing the single point charge (SPC) model [55]. A proper number of counterions (sodium and chloride ions) were added to ensure the electrical neutrality of the whole system and induce salt concentrations of 0.15 M. All the simulations were performed adopting the same computational scheme: (i) after an energy minimization, the whole system was slowly heated up to 300 K using short (100.0 ps) MD runs, (ii) the simulation was extended up to 25 ns for all simulated systems, at 300 K in an isothermal/isobaric ensemble, using the velocity rescaling scheme (temperature) and the isotropic Parinello-Rahman coupling scheme (pressure) [56,57], (iii) the investigated bound complexes were simulated in the GROMOS, a united-atom force field [58], the LINCS algorithm was adopted to constrain all bond lengths [59], and the long-range electrostatics were computed by the Particle Mesh Ewald method [60]. Trajectory analyses were carried out by using the available Gromacs utilities and by the support of VMD graphical interface [61].

The system configuration obtained at 500 ps of each production MD run (vide infra) was selected for a final consensus scoring of each ligand pose. For this purpose, only the water molecules within 5 Å from the center of mass of the bound ligand were included in the model, which then underwent a local minimization with MacroModel (see above). The minimized structures were thus processed using the same approach employed to estimate the ligand–protein affinities in the bound complexes obtained by docking, and, thus, providing a value of consensus scoring for each bound complex.

## 3. Results

### 3.1. Cloning of APOSTART Alleles/Members

Starting from the sequences reported in [13], forward and reverse primers were designed for both 5′ and 3′ rapid amplification of cDNA ends (RACE). Several RACE experiments were required to obtain the entire 5′-end of APOSTART. RACE identified 13 other members of APOSTART in addition to APOSTART1 and APOSTART2 already published in [13]. Following the isolation of the full lengths, we performed end-to-end PCRs with primers specific for each cDNA reconstructed sequence, and obtained complete cDNA and DNA clones of each allele (Table S3). Allele specificity was confirmed in replicated experiments by directly sequencing the amplified products. Therefore, we were able to obtain 13 full-length cDNAs (APOSTART_3 to APOSTART_15) and the relative full-length genomic sequences for six of them (APOSTART_5, APOSTART_6, APOSTART_7, APOSTART_8, APOSTART_10, APOSTART_12) as reported in Table S3.

The final length of the cDNA clones ranged from 2158 nt for APOSTART_14 to 2256 nt for APOSTART_15, while the total length of the eight genomic clones ranged from 5030 nt (APOSTART_6) to 5531 nt (APOSTART_8). The alignment between genomic and full-length cDNA clones confirmed what was already known for APOSTART_1 and APOSTART_2 [13], that all APOSTART members are structured into 21 introns and 22 exons (Figure S2).

Moreover, the average identity of the 15 cDNA clones was 96.87% ranging from 91.48% to 100% (Table S4). The most diverse clone was APOSTART_5 that shared a 93.47% mean identity with other clones (min 91.48% and max 96.18%). The average identity of the 8 genomic clones was 91.70% ranging from 85.33% to 99.58%. The two most diverse clones were APOSTART_6 and APOSTART_5 that shared a mean identity with other clones of 87.11% and 87.49%, respectively (Table S4).

### 3.2. APOSTART Members Are Also or Exclusively Expressed in Flower Tissues

Expression of six APOSTART members was assayed in genotypes with different modes of reproduction by using allele specific primers in the qPCR analyses (Table S2). Allele specificity was verified by directly sequencing an aliquot of the amplified products of each experiment. Reactions were performed in triplicate on independently isolated and retrotranscribed mRNAs from three apomictic and three sexual genotypes. APOSTARTs expression levels were investigated in inflorescences collected during five developmental stages (pre-meiosis, meiosis, post-meiosis, anthesis, and post-anthesis) from sexual and apomictic genotypes as assessed by cytohistological investigations [12,14]. Moreover, two other tissues (leaves and roots) were used in the analysis.

**Figure 1:**
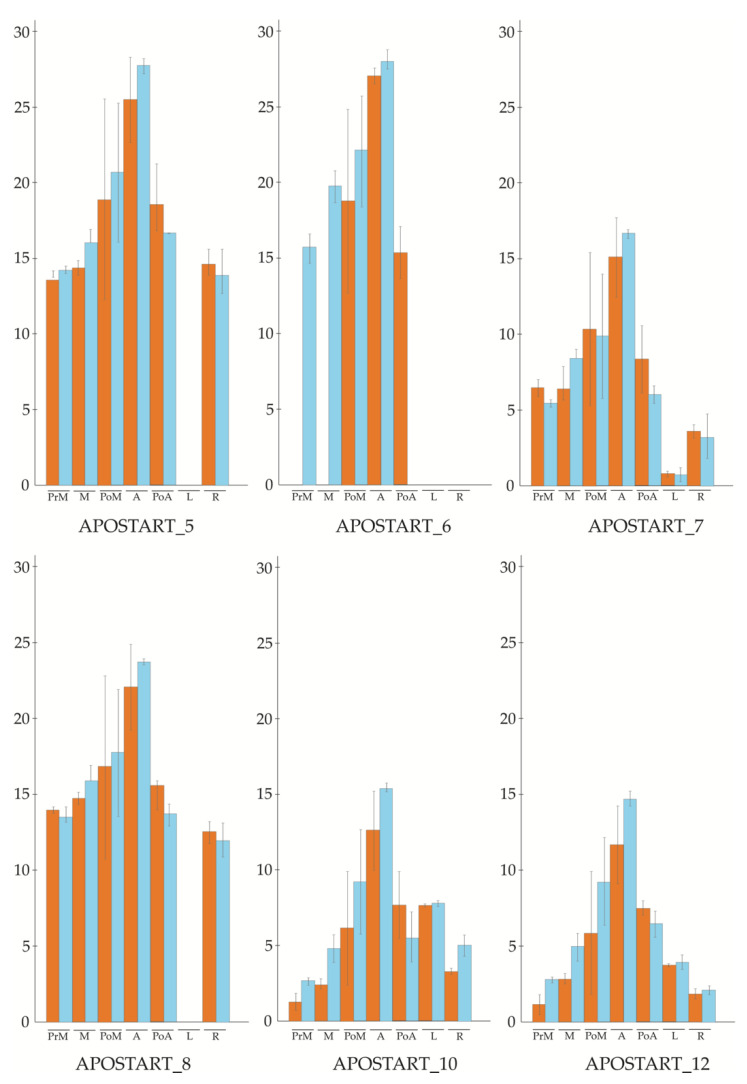
qPCR (quantitative PCR) profile of six APOSTARTs in two different sets of genotypes of *P. pratensis* (orange, apomictic genotypes; blue, sexual genotypes). The expression level was evaluated in five different flowering stages (pre-meiosis; meiosis; post-meiosis; anthesis; post-anthesis) and two tissues (leaves and roots).

Overall, three of the six members investigated (APOSTART_7, APOSTART_10, and APOSTART_12) were expressed in floral tissues as well as in roots and leaves. In all three cases the higher expression was recorded in flower tissues at anthesis, while in leaves and roots it was, on average, lower than in flowers (Figure 1). The expression pattern of the other two members (APOSTART_5 and APOSTART_8) was almost identical as it was expressed in flowers and roots but not in leaves (Figure 1). The most interesting member was APOSTART_6, not only because it showed to be flower specific as its expression was absent both in leaves and roots but also for the marked differences in its expression between apomictic and sexual genotypes (Figure 1).

While in sexual genotypes APOSTART_6 shows an increasing expression level from pre-meiosis to anthesis after which its expression drops to an undetectable level; in apomictic genotypes it is possible to note a shift in expression. In fact, the apomictic genotypes APOSTART_6 starts to be expressed at post-meiosis, and it remains expressed throughout meiosis until the post-anthesis stage.

**Figure 2:**
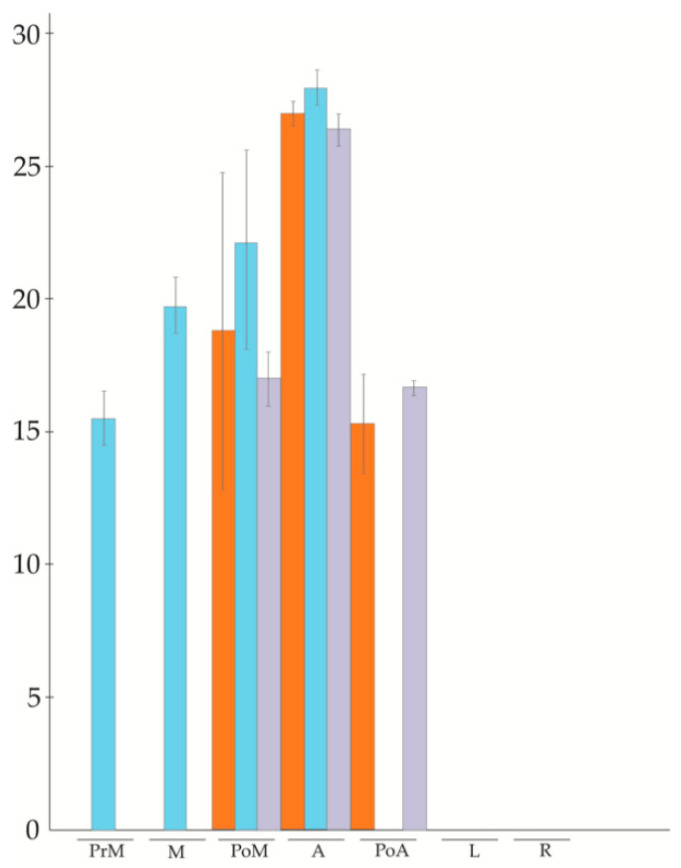
qPCR profile of APOSTART_6 member in three different sets of genotypes (orange, apomictic genotypes; blue, sexual genotypes; purple, parthenogenetic recombinant genotype).

Since our population included one recombinant genotype (non-aposporic and parthenogenetic), we performed a new qPCR analysis for APOSTART_6 including this genotype. As shown in Figure 2, the parthenogenetic genotypes exhibited the same behavior as the apomictic genotypes.

### 3.3. APO-SCAR Cosegregate with Apomixis

**Figure 3:**
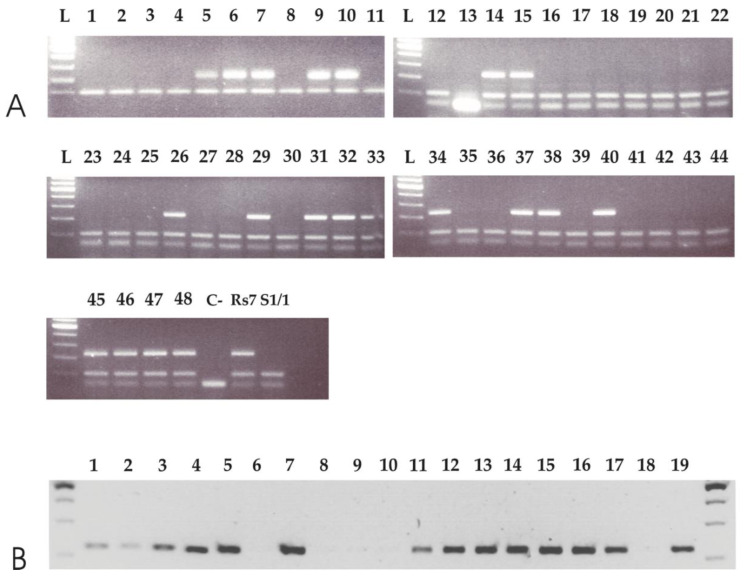
(**A**) APO-SCAR primer pair tested on 48 F1 individuals (lanes 1–48) from a segregating population for the mode of reproduction and obtained by crossing an apomictic (RS7, lane 50) and a sexual (S1/1, lane 51) genotype, as reported in Albertini et al. 2001 [12]. APO-SCAR completely co-segregate with apomixis; (**B**) on exotic germplasm sources of known reproductive behavior (Table S1): lanes 1–5 and 11, apomictic PaPp F_1_ hybrids; lane 6, sexual *P. arachnifera* maternal parent (Pa1FM); lane 7, apomictic KB3 *P. pratensis* pollen parent; lanes 8–10, non-apomictic PaPp F_1_ hybrids; lanes 12–13, apomictic 9601, 1915 *P. pratensis*.

Based on the results of the qPCRs, we were interested in determining if APOSTART_6 co-segregated with apomixis. To do so, we aligned all APOSTART genomic clones (Figure S2) and designed several APOSTART_6-specific primer pairs to test the segregation of the derived marker and its putative association with apomixis. Of all primer pairs evaluated, one, hereafter named APO-SCAR (Table S2, Figure S2 underlined in red), showed to be the best candidate based on preliminary testing. The APO-SCAR primer pair was then tested on an F_1_ population of 68 individuals segregating for the mode of reproduction [12,15,62] and on its parental genotypes (Figure 3A). The APO-SCAR pair of primers produced a single amplification product of 225 bp present in the apomictic paternal genotype but absent in the maternal sexual plant. Figure 3A also shows the complete co-segregation of the APO-SCAR compared with reproductive behavior as assessed by progeny testing [12,15,62]. To further test the efficiency of APO-SCAR in detecting the mode of reproduction, APO-SCAR primers were tested on apomictic and sexual genotypes of *P. pratensis* or *Poa aracnifera* × *P. pratensis* provided by the USDA, Grazinglands Research Laboratory, El Reno, OK, USA all having adaptation to the contrasting environments of Southern and Eastern Europe and the Southern Plains Region of the United States. The reproductive behavior, and the provenance of the analyzed genotypes, are reported in Table S1. The amplification pattern generated by the APO-SCAR primer pair strongly resembled that observed in the mapping progeny and unequivocally distinguished the sexual from the highly apomictic individuals (Figure 3B).

### 3.4. Phylogenetic Cluster Analysis

**Figure 4:**
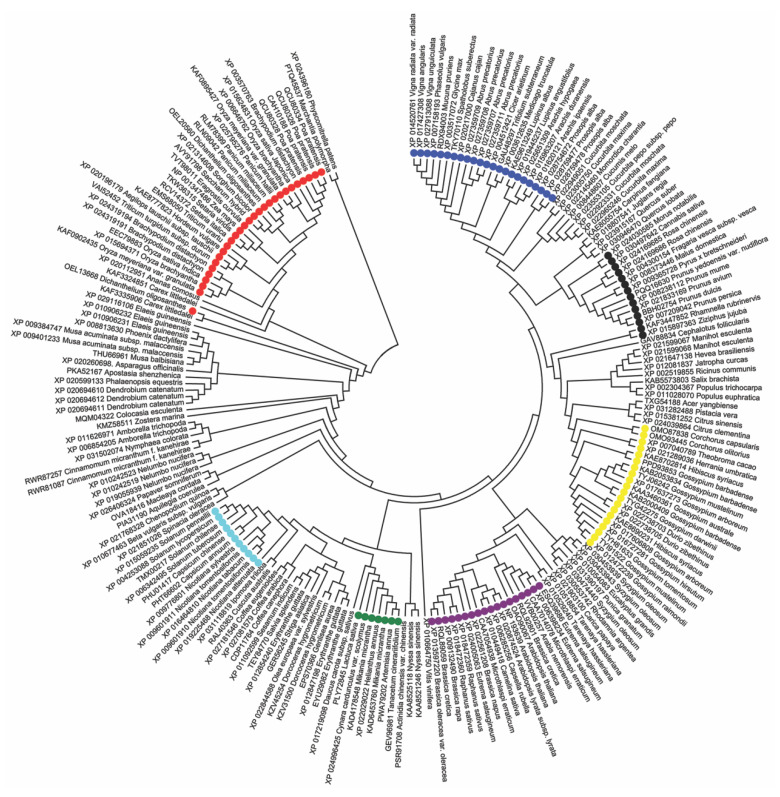
Maximum-likelihood phylogenetic unrooted tree based on APOSTART_6 similar amino acid sequences. The tree has the highest log likelihood (-45391.1656) and is condensed with bootstrap value >70. Phylogenetic analysis was performed using Neighbor-Join and BioNJ algorithms to a matrix of pairwise distances estimated using a JTT model and a discrete γ distribution (5 categories (+G, parameter = 0.6937)). The analysis involved 208 amino acid sequences and was conducted in MEGA7 [23]. Bootstrap values are reported in Figure S3 for more accuracy. Poaceae are indicated in red, Solanaceae in light blue, Asteraceae in green, Malvaceae in yellow, Brassicaceae in purple, Rosaceae in black, and Fagaceae in dark blue.

Since APOSTART_6 completely co-segregated with apomixis, we searched in the BLAST database (https://blast.ncbi.nlm.nih.gov/Blast.cgi) [22] for protein sequences similar to APOSTART_6 and found 208 proteins belonging to 158 species. Protein sequences were aligned with MEGA 7 and resulted in an alignment of 1208 positions. By estimating the pairwise distances obtained using a JTT model of the aligned protein sequences, a maximum-likelihood phylogenetic tree was constructed. The unrooted condensed original tree (Figure 4, Figure S3) is drawn to scale, with branch lengths measured in the number of substitutions per site and a bootstrap value cut-off of 70.

From a first look at the tree, *Physcomitrella patens* and *Marchantia polymorpha*, two non-vascular plants belonging to Bryophyta and Marchantiophyta, respectively, clustered together in an outgroup clade. Genera with more than one sequence (*Oryza*, *Triticum*, *Gossypium*, *Coffea*, etc.) exhibited consistent clustering. *Poa* sequences clustered together as well. The first main cluster in fact includes monocots, in particular the Poaceae family (in red). Continuing counterclockwise, monocots are followed by a few independent taxa (*Amborella*, *Nymphaea*, *Cinnamomum*) belonging to the Magnoliopsida class, intermediate between mono- and dicotyledons. The last large clade contains all the *Eudicots* class plants, divided into very homogeneous groups corresponding to the orders and then to the botanical families. As a matter of fact, it is possible to Solanales, divided in Solanaceae (in light blue) and Convolvulaceae, Lamiales with several families, Asteraceae (in green), Malvaceae (in yellow), Malpighiales with Salicaceae and Euphorbiaceae, Brassicales, divided in Brassicaceae to which *Arabidopsis* belongs (in purple), Cleomaceae and Caricaceae, Myrtales, Fagales, Cucurbitaceae, and Fabaceae (in dark blue).

Overall, only one sequence of *Dichanthelium oligosanthes* is not in the Poaceae cluster and Rosales (in black), with Rhamnaceae on one side and Rosaceae, Cannabaceae, and Moraceae on the other, clustered in independent clades, suggesting that APOSTART_6 phylogeny is strictly dependent on systematic botany.

### 3.5. In Situ Hybridization (ISH)

**Figure 5:**
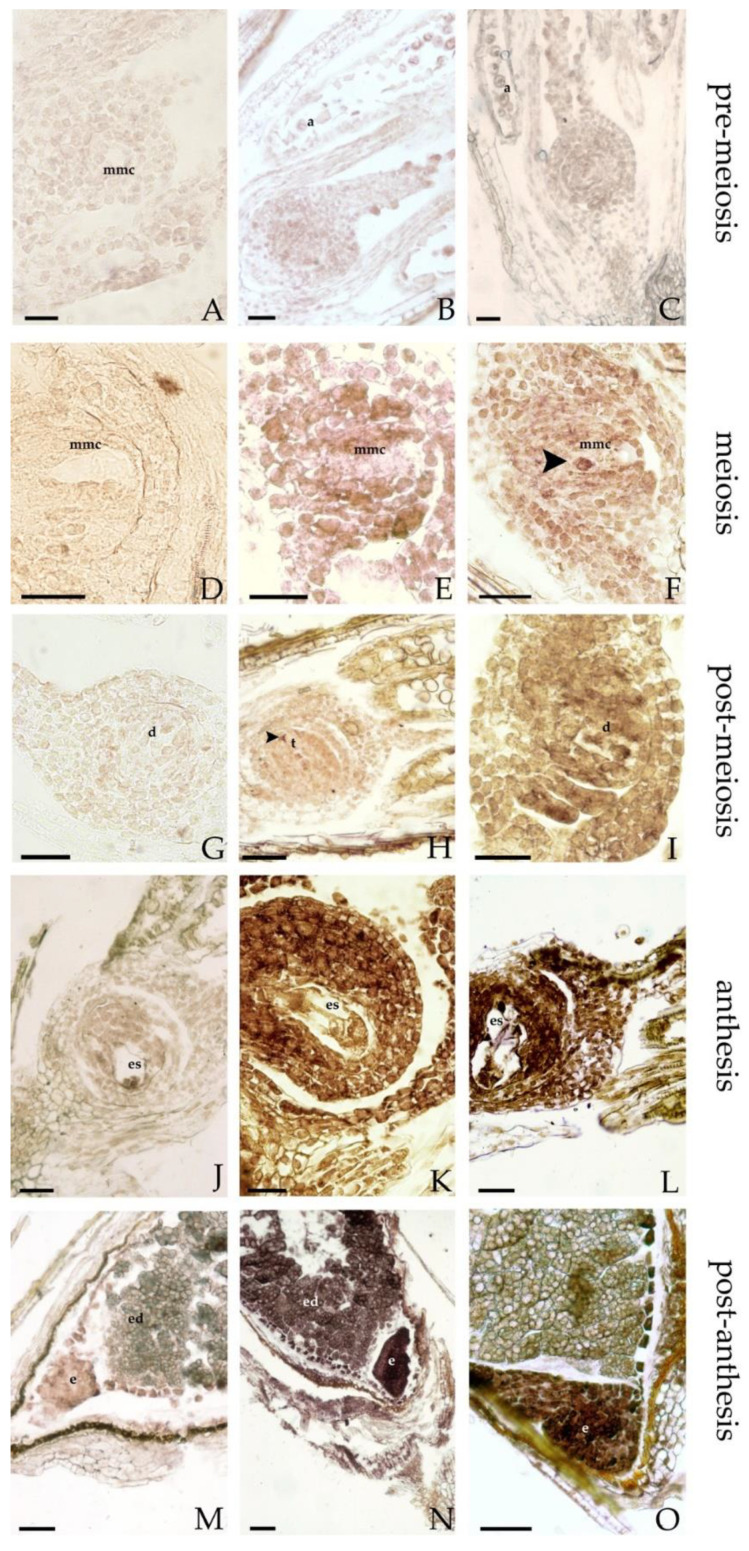
APOSTART expression in longitudinal sections of flowers studied by ISH. Sections of sexual and apomictic genotypes were probed with DIG-labeled antisense (**B**,**C**,**E**,**F**,**H**,**I**,**K**,**L**,**N**,**O**) or sense (**A**,**D**,**G**,**J**,**M**) RNAs and viewed under a microscope bright field that gives a purple label. No signals were detected in the sections hybridized with the sense probes ((**A**,**J**,**M**) sexual genotypes; (**D**,**G**) apomictic genotypes). (**B**,**C**), longitudinal sections of ovules of sexual (**B**) and apomictic (**C**) genotypes containing the MMC, in both genotypes a poor hybridization signal was observed. In sexual (**E**) and apomictic (**F**) genotype ovules during megasporogenesis, the hybridization signal detected in the MMC was the same background in both genotypes, while in apomictic ovules a strong signal was observed in the aposporic cells (indicated by a black arrow) near the MMC (**F**). (**H**,**I**), dyads and tetrads observed in ovules of sexual (**H**) and apomictic (**I**) genotypes have the same signal the background unless they are destined to degenerate. (**K**,**L**), at the anthesis the hybridization signal was strong in the sexual embryo sac (**K**) and stronger in the apomictic embryo sacs (**L**). (**N**,**O**), a strong hybridization signal was observed also in the embryo of sexual (**N**) and apomictic (**O**) genotypes. **a** = anther; **d** = dyad; **e** = embryo; **ed** = endosperm; **es** = embryo sac; **mmc** = megaspore mother cell; **t** = tetrad. Bars = 40 μm.

APOSTART expression was investigated by in situ hybridization (ISH) in longitudinal sections of sexual and apomictic flowers. In a previous analysis [14], APOSTART transcripts were detected both in male and female meiosis in micro- and megaspores. APOSTART was expressed both during megasporogenesis and megagametogenesis, from megaspore mother cells (MMCs) to the mature embryo sac developmental stages. Moreover, it was also expressed during embryo sac development. The new analysis, herein reported, studied the expression of APOSTART considering a wider range in reproduction processes, from pre-meiosis to embryo stage. During pre-meiosis in both reproductive behaviors (apomictic and sexual) a poor hybridization signal was observed in all tissues of the ovule and in the MMCs (Figure 5A–C). During meiosis the hybridization signal detected in the MMC was the same as that of the neighboring nucellar cells (Figure 5E,F), but a strong signal was observed in one or more nucellar cells, detected in the apomictic genotypes near the MMC, that can be the cells that putatively change their fate and become aposporous initials (Figure 5F, black arrow). At post-meiosis stage, before the degeneration of non-functional megaspores, both genotypes’ dyads and tetrads exhibited the identical signal in the background; instead, in the degenerating cells of dyads (Figure 5H, black arrow) or tetrads the hybridization signal was much weaker than that of the nucellar cells (Figure 5H,I). At anthesis (Figure 5K,L) APOSTART expression was strong in all tissues of the ovule but particularly in the embryo sac. Moreover, the expression of the APOSTART appeared stronger in the multiple apomictic embryo sac (Figure 5L). After anthesis, a strong hybridization signal was observed in the embryos of sexual and apomictic genotypes (Figure 5N,O), while a weaker nonspecific signal was detected in the aleuronic layer due to endogenous phosphatase activity.

### 3.6. Localization, Phosphorylation Sites, and Secondary Structure Predictions of PpAPOSTART Proteins

We predicted the subcellular localizations of the 15 APOSTART proteins based on amino acid sequence motifs. We used six different software, and their outputs were not coincident. Despite this, it is evident that the most predicted localization for PpAPOSTART proteins is the mitochondrion and the nucleus (Table S5).

Moreover, using NetPhos 3.1 software [25] we significantly predicted (with a threshold >0.5) serine, threonine, and tyrosine phosphorylation on the 15 APOSTARTs isolated from *P. pratensis* (Table S6). For serine, we observed an average of 46.86 loci (ST.DEV ± 2.09) ranging from 41 to 50 for APOSTART_14 and APOSTART_15, respectively. A lower number of significant loci (24 ± 1.85 sites) were recorded for threonine, and this ranged from 22 (APOSTART_11 and APOSTART_8) to 28 (APOSTART_15). The lowest number of phosphorylation sites (8.2 ± 1.52) that ranged from 7 (APOSTART_4, APOSTART_7, APOSTART_12, APOSTART_13, and APOSTART_14) to 13 (APOSTART_10) was recorded for tyrosines (Table S6).

The datasets were analyzed using the PCoA ordination method and by employing the Bray–Curtis distance matrix [63]. In the PCoA for serine phosphorylation eleven out of 15 APOSTARTs were clearly separated by coordinate 1 (26.44%) in two groups. The other four proteins (APOSTART_6, APOSTART_1, APOSTART_10 and APOSTART_8) were separated (Figure S4A).

Coordinate 2 (15.83%) distinguished APOSTART_13, APOSTART_14, and APOSTART_11 from the others. In the PCoA of threonines phosphorylation coordinate 1 (29.63%) separated 13 out of 15 APOSTART proteins into three groups (Figure S4B) from which APOSTART_6 and APOSTART_2 were excluded. Coordinate 2 (18.29%) was able to differentiate APOSTART_10 and APOSTART_15 from the others. In the PCoA for tyrosine, coordinate1 (36.88%) separated all APOSTART proteins with the exception of APOSTART_10 into three main groups (Figure S4C), while the second coordinate (22.31%) separated APOSTART_2, APOSTART_6, and APOSTART_15 from the others.

Sequences of all the proteins were also analyzed for secondary structure similarities. The secondary structure is defined by the pattern of hydrogen bonds of the protein, such as α-helices, β-sheets, and coils that are observed in an atomic-resolution structure. All the *P. pratensis* APOSTART proteins and all APOSTART-like proteins (in monocots and eudicots) scored in the phylogenetic analysis were tested for protein structure prediction analyses.

PpAPOSTART proteins consist on average of 12.8% α-helix, 26.6% β-sheet, and 57.9% coil (Table S7). In monocots, APOSTART-like proteins are composed on average of 13.2% α-helix, 28.4% β-sheet, and 57% coil, while in eudicots are on average composed of: 13.2% α-helix, 28.7% β-sheet, and 56.7% coil (Table S8). These data do not show statistical differences of secondary structure between monocots and eudicots within the *P. pratensis* APOSTART protein structures. In addition, solvent accessibility was considered as another parameter to characterize the surface area of the protein. The relevance of this measure may explain how different protein shapes have different solvent accessibilities (distinct in exposed and buried sites). This may reveal that the examined genes do not share the same 3D protein structure, even with high sequence homology. PpAPOSTART proteins showed an average percentage of exposed sites of 48.13%, 23.5% of buried sites, and 26.6% of intermediate sites (Table S7). Monocots, solvent exposed sites indicated an average of 48.5%, 24% of buried sites, and 25.9% of intermediate zones. Eudicots show an average of 48.9% of exposed sites and a percentage of 25.7% of buried sites, with 24.7% of undetermined zones. When compared, no statistical differences were found between monocots and eudicots and between the PpAPOSTART and APOSTART-like proteins (Table S8).

A third parameter considered was the intrinsic protein disorder (IDP). IDP regions fail to form a stable structure and are characterized by a low content of bulky hydrophobic amino acids and a high proportion of polar and charged amino acids. This is usually referred to as low hydrophobicity [43]. Furthermore, high net charges promote disorder because of electrostatic repulsion resulting from equally charged residues. A lack of this structure provides a larger interaction surface area that allows bindings with several other proteins. Because of this, IDPs are enriched in signaling and regulatory functions. Up to 33% of eukaryotic proteins are thought to have disordered segments to some degree [44]. In this context, disorder content prediction (DISO) comparison was employed as a tool to speculatively delineate proteins that share similar gene sequences. This analysis (see Tables S7 and S8) shows that *P. pratensis* have a disordered segment percentage of 26.8% higher with respect to the monocots and eudicots paralogs genes with a DISO average of 25.5% and 25%, respectively.

### 3.7. Molecular Modeling and Dynamics

Several lines of evidence have indicated the role of START domain in the shuttling of steroids and other biological lipids between the mitochondria compartments and may suggest a possible relationship between the expression of APOSTART proteins and the trafficking of phytosterols.

To corroborate this hypothesis, the 3D structures of APOSTARTs START domains and their interaction with phytosterols, namely stigmasterol, brassicasterol, and campesterol, were modeled by using a computational workflow (Figure S1) based on the combination of comparative homology modeling and molecular mechanics approaches. To do so, the protein sequences coded by APOSTART_1, APOSTART_6, and APOSTART_8 genes were chosen as representative of the three main clusters extracted by the multiple sequence alignment restricted to the START domain (see Figure S5). Initially 3D structures of the START domain were generated through the comparative modeling of APOSTART proteins to a template structure reported in the RCSB-PDB (Research Collaboratory for Structural Bioinformatics Protein Data Bank) archive (https://www.rcsb.org/) [64], coded 1LN1, whose START domain was discovered to be co-crystallized with a membrane lipid [65]. Thus, 203 residues were correctly modeled, thus corresponding to approximately 73% of the sub-sequences labelled as START domains. The 3D structures of APOSTART_1, APOSTART_6, and APOSTART_8 obtained by the homology modeling investigations are almost superimposable and are very close to the template model (Figure S6). The large binding site region on both proteins is composed of a β-sheet pavement comprising residues from 20 to 46 (residue numbering referred to the protein sequence processed by homology modeling) (β1-3) and from 136 to 167 (β8-9), which is buried on the top by two small helices (52 to 61 and 64 to 70 assigned to α 2 and 3, respectively) and surrounded by a loop from 168 to 171 (ω11) and by the extended C-terminal helix from 172 to 198 (α4). On these protein models, the most plausible binding modes, i.e., poses, of the three considered phytosterols, were identified by the use of docking approaches and a consensus scoring procedure for the evaluation of the protein-ligand affinity on each calculated bound system. Two top scored poses per ligand were eventually detected on either modeled protein thus yielding to 18 bound complexes. Overall, two different orientations of the steroid ligand were appreciated in the top scored poses characterized by the distal or vicinal position of hydroxyl group with respect to the C-terminus; these orientations resembled the IN or OUT mode, respectively, reported by Murcia at al. [66]. MD (Molecular Dynamics) calculations were then carried out by soaking the bimolecular complexes obtained at the docking stage in a box simulating the physiological aqueous medium, which yielded a more realistic description of the solvation effect on both the protein structure and the ligand binding. MD simulations corresponding to 250 ns trajectories were performed for the 18 bound complexes; however, we found a substantial loss of secondary structure, mainly affecting the N-terminal and the C-terminal domains in most of the considered systems after only 500 ps.

**Figure 6:**
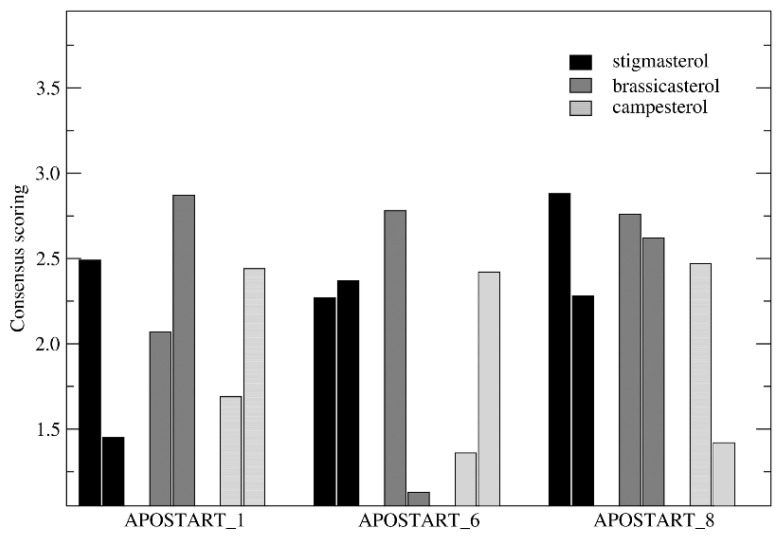
Consensus scoring of stigmasterol (black), brassicasterol (dark gray), and campesterol (light gray) poses binding at APOSTART_1 (left), APOSTART_6 (middle), and APOSTART_8 (right) obtained after 500 ps of MD (Molecular Dynamics) simulation (vide infra).

This structural detriment may be explained by assuming that the START domain of these proteins could be stabilized by the interaction with other domains of the APOSTART coded protein, such as the PH domain. Although this result substantially reduced the “informative potential” of the MD analyses, we employed the molecular systems obtained at 500 ps of each trajectory (treated as reported in the method section) in which the secondary structure was conserved in all-molecular systems to assay the effect of explicit solvation on the physterol binding properties. At this point, consensus scoring calculations were performed on the 18 sampled bound complexes yielding to the top scored poses of stigmasterol, brassicasterol, and campesterol at APOSTART_1, APOSTART_6, and APOSTART_8. The results of consensus scoring (performed as described in the previous section) are reported in Figure 6.

**Figure 7:**
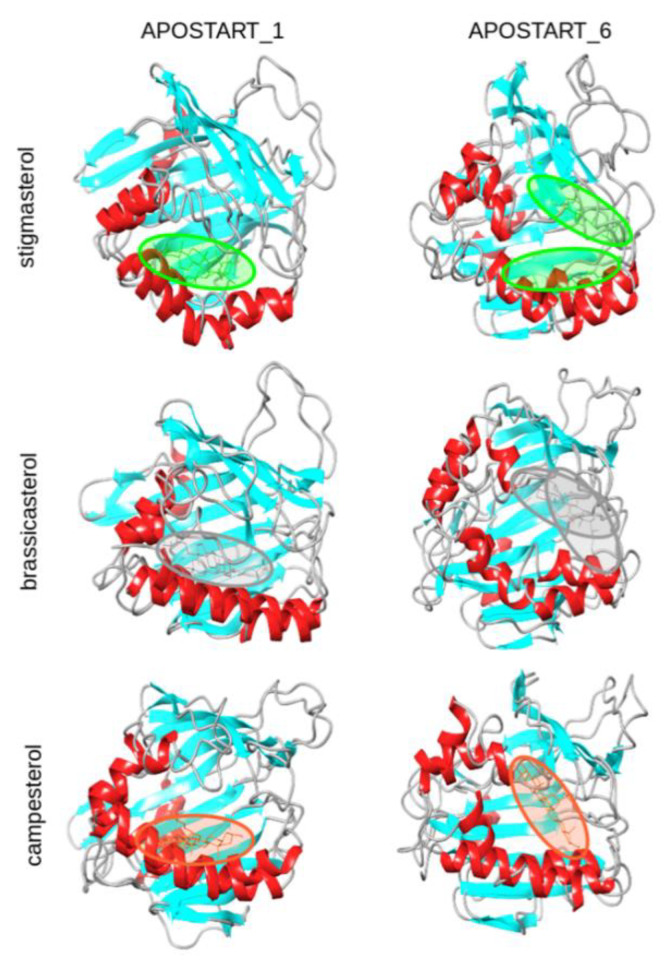
Representation of the binding complexes of APOSTART_1 and APOSTART_6 (cartoon) with the considered phytosterols (sticks). Binding sites of stigmasterol (green), brassicasterol (gray), and campesterol (orange) at the two proteins are also displayed.

As shown, ten of the 18 bound systems were characterized by score values higher than 2.3 (corresponding to approximately the difference between the maximum score, 2.88, and the standard deviation on the whole set, 0.56) and were identified as top-scoring and analyzed at major details. The poses of the investigated phytosterols binding at APOSTART_1 and APOSTART_6 is reported in Figure 7.

It is worth noting that two binding site regions were eventually detected in the calculated molecular systems of either APOSTART_1 and APOSTART_6 (Figure 7). Four of the six top scored complexes, including the bound complexes of APOSTART_1 and stigmasterol binding at APOSTART_6, presented a hydrophobic binding region, which hosted the polycyclic steroid moiety of ligands independently on its IN or OUT orientation, with a majority formed in the inner wall of the C-terminal helix domain. In this binding region, residues L179, C184, L188, and Y191 on α4 were identified as mainly involved in the hydrophobic interaction with these bound steroids, whereas the hydrophilic interactions of the ligand hydroxyl group may involve H156 or water molecules depending on the IN or OUT, respectively, binding mode (Figure S7). On the other hand, the bound complexes of brassicasterol and campesterol at APOSTART_6 were found to also be positioned in another region comprising the hydrophobic residues M89, V91, and P93 on β5 and residues L167, Y175, and L179 on ω11. Hydrophilic contacts of the ligand OH group with residue E70 and/or with water molecules were also detected. It is worth noting that the two bound complexes of stigmasterol at APOSTART_6 were both characterized by a moderately high score. By this consideration, it may be concluded that the APOSTART_1 binding site is mainly localized in proximity of the inner wall of the C-terminal helix domain, whereas in the bound complexes of APOSTART_6 steroids are more likely to be positioned in the region between the β5 and ω11 domains.

## 4. Discussion

A successful transfer of apomixis to non-apomictics will allow clonal seed production in a myriad of crop species. Hopefully, this event will reduce input costs of fruit and vegetable production, while also raising yield [67]. A better understanding of the inheritance patterns for apomixis is fundamental for facilitating the identification of candidate genes, which in turn, is essential for engineering apomixis into sexual crops [68].

In a previous work [13] we have isolated APOSTART, a gene containing three domains, one of which is START whose name was given after the discovery of the *StAR* gene that is involved in human congenital lipoid adrenal hyperplasia. The clinical phenotype of this disease includes male pseudo hermaphroditism resulting from deficient fetal testicular testosterone synthesis. In apospory, a cell of the nucellus becomes an aposporous initial and then develops into a nonreduced embryo sac, which through parthenogenesis, gives rise to a viable embryo. How and why somatic cells of the ovule change their developmental fate and gain embryogenic potency is unknown. This question, together with the features of the *StAR* gene, made APOSTART our primary candidate for the control of apomixis. As a consequence, we decided to further characterize its expression, structure, and inheritance. Our data seem to confirm our vision. If we take all PpAPOSTARTs into consideration, we can see that while some of them are expressed in all tissues even if sometimes differentially between apomictic and sexual genotypes (i.e., APOSTART_10 and APOSTART_12), APOSTART_6 is the only PpAPOSTART specifically expressed in tissue flowers. Moreover APOSTART_6 showed a delayed expression in apomictic genotypes when compared with sexual ones, and this tendency was confirmed in a parthenogenetic individual. Both sexual reproduction and apomixis involve life-cycle renewal from gamete or gamete-like cells following ploidy restitution. The difference in expression timing between apomictic and sexual genotypes may imply that APOSTART_6 is the key regulator that switches a “normal” nucellar cell into an aposporous initial.

In addition, as shown by ISH, in apomictic genotypes a strong signal was detected only in some nucellar cells neighboring the MMC. This confirmed the hypothesis that *APOSTART* is involved in the change of fate of these cells and, therefore, in embryo sac development from nucellar cells.

Based on specific polymorphisms between PpAPOSTARTs, we developed some member-specific primer pairs, and the one designed for APOSTART_6 co-segregated with apomixis and was renamed APO-SCAR. This, together with the qPCR data, made APOSTART_6 the candidate to control apomixis in *P. pratensis*.

Moreover, an important quality of SCAR markers is their suitability on different genetic backgrounds. We then tested APO-SCAR on material belonging to different areas and continents and confirmed its capability to discriminate apomictic versus sexual genotypes. Therefore, APO-SCAR can be valuable tool for use in breeding and selection programs, considering that among the practical choices for minimizing time and costs of breeding programs, an easy method for early selection based on molecular markers would be of major advantage.

The subcellular localization predicts that PpAPOSTART might be localized in the mitochondrion or nucleus, although the six different software programs we used provided contrasting results. Nevertheless, a comparison with two proteins belonging to *Arabidopsis thaliana*, known as AtAPOSTART_1 and AtEDR2 (or AtAPOSTART_2), that share a high amino acid similarity and identity score with PpAPOSTART proteins could be made. In fact, AtAPO1 protein has been predicted to be localized in mitochondria and plastids [69], while Vorwerk et al. [70] could not co-localize the chimeric EDR2:HA:eGFP with the mitochondrial dye MitoTracker.

Conversions from diplospory to normal tetrad formation was observed in *Boechera* when pistils were subjected to variety of stress treatments, including carbohydrate starvation, osmotic stress, exposure to H_2_O_2_, and inhibition of brassinosteroid synthesis [2,71]. In addition, Antennaria-type diplospory occurred when DNA methylation was suppressed prior to MMC formation [72]. The conversions, from apomictic to sexual, and from one apomeiotic type to another, are evidence that the metabolic status of ovules (and the genes that affect this status) regulate the developmental sex/apomixis decision, and that the type of apomixis expressed is a function of the temporal and spatial expression of perhaps a single apomixis-conferring signal [2]. It has been proposed [73] that *EDR2*, one of the Arabidopsis orthologs of *PpAPOSTART*, may play a role in lipid signaling, mitochondria, and the activation of PCD (Programmed Cell Death) in plants. The prediction of PpAPOSTART localizes in mitochondria matches with this hypothesis. We also suggested [13] that APOSTART expression in *P. pratensis* may be related to the PCD that is involved in the non-functional megaspores and nucellar cell degeneration events that permit enlargement of maturing embryo sacs. The strong signal detected by ISH in degenerating cells of tetrads and in developing embryo sacs and nucellar cells in close proximity would appear to confirm this hypothesis.

Moreover, even if our tests to identify phosphorylation sites are predictions, they provided interesting hints about the potential post-translational different functions of the 15 PpAPOSTART identified. In serine, threonine, and tyrosine PCoA some PpAPOSTART members showed a clear differentiation from all others and, in particular, APOSTART_6 was separated from other members in all tests. This suggests a potential unique post-translational molecular function, in line with its flower-specific expression, and could represent a sign of its specific, important role in the cell changing fate and/or the apomictic embryo sac development.

Due to cell proliferation during evolution, the START domain acquired two distinct functions: the first appears to be generally related to the stress response [74,75,76] and the second to signaling mediated by lipid binding. In the last, the START domain underwent the lineage-specific fusions to other effector domains that are typical of multidomain eukaryotic signaling proteins. At least 60 START-containing multidomain proteins have been found in *Arabidopsis* and rice.

Sterols are essential membrane components and are critical for many physiological processes in all eukaryotes. In plants, it has been demonstrated that sexual reproduction is highly affected when the ratio of campesterol to sitosterol differs by a factor of 10, and yet vegetative growth such as stem elongation is only moderately affected when the ratio of campesterol to sitosterol differs by a factor of 30 to the wild-type [77]. Moreover, when stigmasterol is above a threshold level in the diets of grasshoppers [78] and aphids [79], these insects show high mortality and low reproduction. Therefore, determining the lipid or sterol molecule bound by the START domain would be an important step in unraveling the role of PpAPOSTART. Sterols are essential molecules for embryogenesis, and the high signal observed in an embryo during ISH could be related to this role of APOSTART. Therefore, the possible binding of stigmasterol, campesterol, and brassicasterol at the START domain of APOSTART_1, APOSTART_6, and APOSTART_8 coded proteins were investigated by means of a multilayered computational procedure. Calculations indicated that these three phytosterols effectively bind at the START domain of the modeled proteins, although differences were unveiled in the binding site structures. In APOSTART_1, all tested sterols bind at the same hydrophobic region, while a different binding pocket was found in APOSTART_12, thus probably reflecting a different responsiveness of the modeled proteins to phytosterols.

Recently, using CRISPR-Cas9, some researchers attempted to set up synthetic apomixis in rice but a maximum of 29% of offspring were maternal clones [80,81,82]. Here we have demonstrated that PpAPOSTART is expressed in different cells (MMC vs. Nucellar), and for one of its members (APOSTART_6), the expression is delayed confirming the hypothesis that the shift in timing and place of expression may cause apomixis or, at least, one of the two components of apomictic development. Since our results showed the closeness of three *Oryza* spp. sequences to PpAPOSTARTs, this makes APOSTART a gold candidate for future editing approaches for improving the maternal-like rate in rice progenies.

## Supplementary Materials

The following are available online at https://www.mdpi.com/2073-4425/11/8/941/s1, Figure S1: Computational workflow used to model phytosterol-APOSTART_# binding complexes, Figure S2: Nucleotidic alignment of 15 APOSTART cDNA and 8 APOSTART genomic clones, Figure S3: Maximum-likelihood phylogenetic unrooted tree based on APOSTART_6 similar amino acid sequences, Figure S4: Bidimensional PCoA ordination method on predicted phosphorylation sites, Figure S5: Evolutionary relationships of the START domain aminoacidic sequences belonging to the 15 APOSTART members, Figure S6: Comparison between the calculated structure of the START domain of APOSTART_1, Figure S7: Maps of the ligand–protein interactions detected in the binding complexes of APOSTART_1 and APOSTART_6, Table S1: Information on germplasm source mode of reproduction, number of genotypes analyses and results of the APO-SCAR, Table S2: Sequence of primers used, Table S3: List of PpAPOSTART cDNA and genomic clones, Table S4: Identity values between either APOSTART genomic or APOSTART cDNA clones, Table S5: APOSTART proteins subcellular localization, Table S6: Predicted phosphorylation sites, Table S7: PpAPOSTART protein secondary structures predictions, Table S8: APOSTART and APOSTART-like proteins’ secondary structures predictions.
