# Supplementary material for: The Role of APOSTART in Switching between Sexuality and Apomixis in Poa pratensis
Source: Genes (Basel). 2020 Aug 14;11(8):941. doi: 10.3390/genes11080941 (PMC7464379; doi:10.3390/genes11080941)
Supplement: Supplementary file 1 [file genes-11-00941-s001.zip › Supplementary Figures/Figure S2.pdf]

Figure S2. Nucleotidic alignment of 15 APOSTART cDNA and 8 APOSTART genomic clones.

```
                                                                                                                    Section 1
                  (1) 1      10        20        30        40        50        60        70        80        90        100       110       120         134
APOSTART 1        (1) ATGGTGCGCTACGGCCGGCGCAAGATCGGCAGGTCAATCTTCCAAACGCGCTACTTCGTGCTCGAGAGCAAGCTGCTCGCCTACTACAAGAAGAAGCCCAAGGACAGCATG-----------------------
APOSTART 2        (1) ATGGTGCGGTACGGCCGGCGCAAGATCGGTAGGTCCTTCTTCCACACACGCTACTTCGTGCTCGAGAGCAAGCTGCTCGCCTACTACAAGAAGAAGCCCAAGGACAGCATG-----------------------
APOSTART_3        (1) ATGGTGCGCTACGGCCGGCGCAAGATCGGCAGGTCAATCTTCCAAACGCGCTACTTCGTGCTCGAGAGCAAGCTGCTCGCCTACTACAAGAAGAAGCCCAAGGACAGCATG-----------------------
APOSTART_4        (1) ATGGTGCGCTACGGCCGGCGCAAGATCGGCAGGTCAATCTTCCAAACGCGCTACTTCGTGCTCGAGAGCAAGCTGCTCGCCTACTACAAGAAGAAGCCCAAGGACAGCATG-----------------------
APOSTART_5        (1) ATGGTGCGCTACGGCCGGCGCAAGATCGGCAGGTCAATCTTCCAAACGCGCTACTTCGTGCTCGAGAGCAAGCTGCTCGCCTACTACAAGAAGAAGCCCAAGGACAGCATG-----------------------
APOSTART_6        (1) ATGGTGCGGTACGGCCGGCGCAAGATCGGTAGGTCCTTCTTCCACACGCGCTACTTCGTGCTCGAGTCCAAGCTGCTCGCCTACTACAAGAAGAAGCCCAAGGACAGCATG-----------------------
APOSTART 7        (1) ATGGTGCGCTACGGCCGGCGCAAGATCGGCAGGTCAATCTTCCAAACGCGCTACTTCGTGCTCGAGACCAAGCTGCTCGCCTACTACAAGAAGAAGCCCAAGGACAGCATG-----------------------
APOSTART_8        (1) ATGGTGCGCTACGGCCGGCGCAAGATCGGCAGGTCAATCTTCCAAACGCGCTACTTCGTGCTCGAGAGCAAGCTGCTCGCCTACTACAAGAAGAAGCCCAAGGACAGCATG-----------------------
APOSTART_9        (1) ATGGTGCGCTACGGCCGGCGCAAGATCGGCAGGTCAATCTTCCAAACGCGCTACTTCGTGCTCGAGAGCAAGCTGCTCGCCTACTACAAGAAGAAGCCCAAGGACAGCATG-----------------------
APOSTART_10       (1) ATGGTGCGGTACGGCCGGCGCAAGATCGGTAGGTCCTTCTTCCACACACGTTAATTCGTGCTCGAGAGCAAGCTGCTCGCCTACTACAAGAAGAAGCCCAAGGACAGCATG-----------------------
APOSTART_11       (1) ATGGTGCGCTACGGCCGGCGCAAGATCGGCAGGTCAATCTTCCAAACGCGCTACTTCGTGCTCGAGAGCAAGCTGCTCGCCTACTACAAGAAGAAGCCCAAGGACAGCATG-----------------------
APOSTART_12       (1) ATGGTGCGCTACGGCCGGCGCAAGATCGGCAGGTCAATCTTCCAAACGCGCTACTTCGTGCTCGAGACCAAGCTGCTCGCCTACTACAAGAAGAAGCCCAAGGACAGCATG-----------------------
APOSTART_14       (1) ATGGTGCGCTACGGCCGGCGCAAGATCGGCAGGTCAATCTTCCAAACGCGCTACTTCGTGCTCGAGACCAAGCTGCTCGCCTACTACAAGAAGAAGCCCAAGGACAGCATG-----------------------
APOSTART_15       (1) ATGGTGCGCTACGGCCGGCGCAAGATCGGCAGGTCAATCTTCCAAACGCGCTACTTCGTGCTCGAGACCAAGCTGCTCGCCTACTACAAGAAGAAGCCCAAGGACAGCATG-----------------------
APOSTART_13       (1) ATGGTGCGCTACGGCCGGCGCAAGATCGGCAGGTCAATCTTCCAAACGCGCTACTTCGTGCTCGAGACCAAGCTGCTCGCCTACTACAAGAAGAAGCCCAAGGACAGCATG-----------------------
APOSTART_1_GEN    (1) ATGGTGCGCTACGGCCGGCGCAAGATCGGCAGGTCAATCTTCCAAACGCGCTACTTCGTGCTCGAGAGCAAGCTGCTCGCCTACTACAAGAAGAAGCCCAAGGACAGCATGGTACGTA----AATTATTCGATC
APOSTART_2_GEN    (1) ATGGTGCGGTACGGCCGGCGCAAGATCGGTAGGTCCTTCTTCCACACACGCTACTTCGTGCTCGAGGGCAAGCTGCTCGCCTACTACAAGAAGAAGCCCAAGGACAGCATGGTACGTA----AATTATTCAATC
APOSTART_5_GEN    (1) ATGGTGCGCTACGGCCGGCGCAAGATCGGCAGGTCAATCTTCCAAACGCGCTACTTCGTGCTCGAGAGCAAGCTGCTCGCCTACTACAAGAAGAAGCCCAAGGACAGCATGGTACGCC----CATCATTCCATC
APOSTART_6_GEN    (1) ATGGTGCGGTACGGCCGGCGCAAGATCGGTAGGTCCTTCTTCCACACGCGCTACTTCGTGCTCGAGTCCAAGCTGCTCGCCTACTACAAGAAGAAGCCCAAGGACAGCATGGTACGTA----C-----------
APOSTART_7_GEN    (1) ATGGTGCGCTACGGCCGGCGCAAGATCGGCAGGTCAATCTTCCAAACGCGCTACTTCGTGCTCGAGACCAAGCTGCTCGCCTACTACAAGAAGAAGCCCAAGGACAGCATGGTACGTATGTAAATTATTCGATC
APOSTART_8_GEN    (1) ATGGTGCGCTACGGCCGGCGCAAGATCGGCAGGTCAATCTTCCAAACGCGCTACTTCGTGCTCGAGAGCAAGCTGCTCGCCTACTACAAGAAGAAGCCCAAGGACAGCATGGTACGTA----AATTATTCGATC
APOSTART_10_GEN   (1) ATGGTGCGGTACGGCCGGCGCAAGATCGGTAGGTCCTTCTTCCACACACGTTAATTCGTGCTCGAGAGCAAGCTGCTCGCCTACTACAAGAAGAAGCCCAAGGACAGCATGGTACGTA----AATTATTCGATC
APOSTART_12_GEN   (1) ATGGTGCGCTACGGCCGGCGCAAGATCGGCAGGTCAATCTTCCAAACGCGCTACTTCGTGCTCGAGACCAAGCTGCTCGCCTACTACAAGAAGAAGCCCAAGGACAGCATGGTACGTA----AATTATTCGATC
                                                                                                                    Section 2
                (135) 135 140       150       160       170       180       190       200       210       220       230       240       250              268
APOSTART 1      (112) --------------------------------------------------------------------------------------------------------------------------------------
APOSTART 2      (112) --------------------------------------------------------------------------------------------------------------------------------------
APOSTART_3      (112) --------------------------------------------------------------------------------------------------------------------------------------
APOSTART_4      (112) --------------------------------------------------------------------------------------------------------------------------------------
APOSTART_5      (112) --------------------------------------------------------------------------------------------------------------------------------------
APOSTART_6      (112) --------------------------------------------------------------------------------------------------------------------------------------
APOSTART 7      (112) --------------------------------------------------------------------------------------------------------------------------------------
APOSTART_8      (112) --------------------------------------------------------------------------------------------------------------------------------------
APOSTART_9      (112) --------------------------------------------------------------------------------------------------------------------------------------
APOSTART_10     (112) --------------------------------------------------------------------------------------------------------------------------------------
APOSTART_11     (112) --------------------------------------------------------------------------------------------------------------------------------------
APOSTART_12     (112) --------------------------------------------------------------------------------------------------------------------------------------
APOSTART_14     (112) --------------------------------------------------------------------------------------------------------------------------------------
APOSTART_15     (112) --------------------------------------------------------------------------------------------------------------------------------------
APOSTART_13     (112) --------------------------------------------------------------------------------------------------------------------------------------
APOSTART_1_GEN  (131) GAATGTCACAAGAAGAAAGCTTGGATGATTTTTGGGTTTGTCGTTCTCTGATCGACACAATGCTGTGTTTTCTTAAAGGGATACAATGCTGT---GTTCGTAATATGGATCATGGGAACTGTAATTTCGCCGAT
APOSTART_2_GEN  (131) GAATGTCACAAGAAGAAAGCTTGGATGATTTTTGGGTTTGTCGTTCTCTGATCGACACAATGCTGTGTTTTCTTAAAGGGACACAATGCTGT---GTTCGTAATATGGATCATGGGAACTGGAATTTCGCCGAT
APOSTART_5_GEN  (131) GGACTGAATCTGATCAAATTGTCGTGTTCATTGATCATCAAACCACGAACAAATTTTGGAAAATGTGAGAGATTTCTGGATGATCATTTTATTTACTGTTGTCTCATGATC--GATCCCAACAATGTATGGCGT
APOSTART_6_GEN  (120) ----G--------------CCACGCCCATCATTCCATCGGACTGAATCTGATCAAATTGCCCTCGTTGATAGATCAGTGTTGCTTATTGTTC---TCTGCTAATATGGAT--TGTGCATGGAATTCCGTTGTTG
APOSTART_7_GEN  (135) GAATTAATGCCACAGAAAGCTTGGACGATTTTTGG-TTTGTTGTTGTCTGATCGACACAATGCTGTGTGTTCTTAAAGGGACACAATGCTGTATTGTTCGTAATATGGATCATGGGAACTGGAATTTCGTCGAT
APOSTART_8_GEN  (131) GAATGTCACAAGAAGAAAGCTTGGATGATTTTTGGGTTTGTCGTTCTCTGATCGACACAATGCTGTGTTTTCTTAAAGGGATACAATGCTGT---GTTCGTAATATGGATCATGGGAACTGTAATTTCGCCGAT
APOSTART_10_GEN (131) GAATGTCACAAGAAGAAAGCTTGGATGATTTTTGGGTTTGTCGTTCTCTGATCGACACAATGCTGTGTTTTCTTAAAGGGATACAATGCTGT---GTTCGTAATATGGATCATGGGAACTGTAATTTCGCCGAT
APOSTART_12_GEN (131) GAATGTCACAAGAAGAAAGCTTGGATGATTTTTGGGTTTGTCGTTCTCTGATCGACACAATGCTGTGTTTTCTTAAAGGGATACAATGCTGT---GTTCGTAATATGGATCATGGGAACTGTAATTTCGCCGAT
```

Section 3

```
              (269) 269       280       290       300       310       320       330       340       350       360       370       380       390       402
APOSTART 1      (112) ----------------------------------------------------------------------------------------------------------------------------------------
APOSTART 2      (112) ----------------------------------------------------------------------------------------------------------------------------------------
APOSTART_3      (112) ----------------------------------------------------------------------------------------------------------------------------------------
APOSTART_4      (112) ----------------------------------------------------------------------------------------------------------------------------------------
APOSTART_5      (112) ----------------------------------------------------------------------------------------------------------------------------------------
APOSTART_6      (112) ----------------------------------------------------------------------------------------------------------------------------------------
APOSTART 7      (112) ----------------------------------------------------------------------------------------------------------------------------------------
APOSTART_8      (112) ----------------------------------------------------------------------------------------------------------------------------------------
APOSTART_9      (112) ----------------------------------------------------------------------------------------------------------------------------------------
APOSTART_10     (112) ----------------------------------------------------------------------------------------------------------------------------------------
APOSTART_11     (112) ----------------------------------------------------------------------------------------------------------------------------------------
APOSTART_12     (112) ----------------------------------------------------------------------------------------------------------------------------------------
APOSTART_14     (112) ----------------------------------------------------------------------------------------------------------------------------------------
APOSTART_15     (112) ----------------------------------------------------------------------------------------------------------------------------------------
APOSTART_13     (112) ----------------------------------------------------------------------------------------------------------------------------------------
APOSTART_1_GEN  (262) CAAATTGTAATTCTTATGAATGAATGCCAACCTGATAACTTTGTCTTACAGATGCCAATTAACTGTAATTCTTGGTCTGAAACAGAATTGTAATCTTTTTCCTCCCCTGACACATAAGAAATAATTTTATATTT
APOSTART_2_GEN  (262) CAAATTGTAATTCTTATGAATGAATGCCAACCTGATAACTTTGTCTTACAGATGCCAATTAACTGTAATTCTTGGTCTGAAACAGAAATGTAATCTTTTTCCTCCCCTGACACATAAGAAATAATTTTATATTT
APOSTART_5_GEN  (263) GTGTTTTCTGAAAGGGAGGCGCAATGCTGTCTTGCAAAGTGGAATTTCGCCGTTGAAATTTTTCCACTTACAAGGATGTCGGCCCACCCGATAATTTTATCTC-----ACCAATGTCAATTAATTGTAAACTG
APOSTART_6_GEN  (231) AAATTTTCCAATTCAAAGGATGTCGGCCCACCGGATAAATCTATCTCACCAATGTCAATTAA-------------------------TTGTAAACTTTGTGCTCCTCTGACAGATAAAAAGTTTAAAACTGGTT
APOSTART_7_GEN  (268) CAAATTGCAGTTCTTATGAATGAATGCCAACCTGACAACTTTGTCTTACATATGCCAATTAACTGTAATTCTTGGTGTGAAATAGAGTTGTAATCTTTTTTCTCCCCTGACACATCAGAATTAGTTTTATAGTT
APOSTART_8_GEN  (262) CAAATTGTAATTCTTATGAATGAATGCCAACCTGATAACTTTGTCTTACAGATGCCAATTAACTGTAATTCTTGGTCTGAAACAGAATTGTAATCTTTTTCCTCCCCTGACACATAAGAAATAATTTTATATTT
APOSTART_10_GEN (262) CAAATTGTAATTCTTATGAATGAATGCCAACCTGATAACTTTGTCTTACAGATGCCAATTAACTGTAATTCTTGGTCTGAAACAGAATTGTAATCTTTTTCCTCCCCTGACACATAAGAAATAATTTTATATTT
APOSTART_12_GEN (262) CAAATTGCAGTTCTTATGAATGAATGCCAACCTGACAACTTTGTCTTACATATGCCAATTAACTGTAATTCTTGGTGTGAAATAGAGTTGTAATCTTTTTTCTCCCCTGACACATCAGAATTAGTTTTATAGTT
```

Section 4

```
              (403) 403    410       420       430       440       450       460       470       480       490       500       510       520            536
APOSTART 1      (112) ----------------------------------------------------------------------------------------------------------------------------------------
APOSTART 2      (112) ----------------------------------------------------------------------------------------------------------------------------------------
APOSTART_3      (112) ----------------------------------------------------------------------------------------------------------------------------------------
APOSTART_4      (112) ----------------------------------------------------------------------------------------------------------------------------------------
APOSTART_5      (112) ----------------------------------------------------------------------------------------------------------------------------------------
APOSTART_6      (112) ----------------------------------------------------------------------------------------------------------------------------------------
APOSTART 7      (112) ----------------------------------------------------------------------------------------------------------------------------------------
APOSTART_8      (112) ----------------------------------------------------------------------------------------------------------------------------------------
APOSTART_9      (112) ----------------------------------------------------------------------------------------------------------------------------------------
APOSTART_10     (112) ----------------------------------------------------------------------------------------------------------------------------------------
APOSTART_11     (112) ----------------------------------------------------------------------------------------------------------------------------------------
APOSTART_12     (112) ----------------------------------------------------------------------------------------------------------------------------------------
APOSTART_14     (112) ----------------------------------------------------------------------------------------------------------------------------------------
APOSTART_15     (112) ----------------------------------------------------------------------------------------------------------------------------------------
APOSTART_13     (112) ----------------------------------------------------------------------------------------------------------------------------------------
APOSTART_1_GEN  (396) CTTTCAAAAGTATATTTTCT-GCA--TTTTAATTATAATTCTGATATCACTATTTAAT------GATATTCAAAAATTTGATCATGCTGTCTGATGGCTAAATTCAGATAGCACCTCGGAACCTCTGGTTTTTT
APOSTART_2_GEN  (396) CTTTCAAAAGTATATTTTCCAGCATTTTTTTATTATAATTCTGATATCACTATTTAAT------GAAATTCAAAAATTTGATCATGATGTCTGATGGCTAAATTCAGATAGCACCTCGGAACCTCTGGTTTTCT
APOSTART_5_GEN  (392) TGTGCTTTTTACCTGCATTCATGTTATTTCTGATGAACTGATGAGATTA----TTGATCCTAATGTGATTCGAAAAGATGATGCTGC----------CTAAATTCGAAAAG-A-----------TTTGG---C-
APOSTART_6_GEN  (340) TTACCATCATTCATGTT--A------TTTCGGATGAACTGATGAGGTTACTA----ACCCTGATATGATTCGAAAAGATTATTCTGCA----------TAAGTTCCAGTAGCACACTAGA-------TTTG---
APOSTART_7_GEN  (402) GTTTCAATAATAGATTTTTCTGCA-TTTTTTGTTAGAATTCTGATATCACTATTTGATCGTAAAGAAATTCCAAAAGTTTATCGTGCTGTCTGATGGCTAAATTCAGATAGCACCTCGGAACCTCTGGTTTTTT
APOSTART_8_GEN  (396) CTTTCAAAAGTATATTTTCT---GCATTTTAATTATAATTCTGATATCACTATTTAAT------GATATTCAAAAATTTGATCATGCTGTCTGATGGCTAAATTCAGATAGCACCTCGGAACCTCTGGTTTTTT
APOSTART_10_GEN (396) CTTTCAAAAGTATATTTTCT-GCA--TTTTAATTATAATTCTGATATCACTATTTAAT------GATATTCAAAAATTTGATCATGCTGTCTGATGGCTAAATTCAGATAGCACCTCGGAACCTCTGGTTTTTT
APOSTART_12_GEN (396) GTTTCAATAATAGATTTTTC-TGCATTTTTTGTTAGAATTCTGATATCACTATTTGATCGTAAAGAAATTCCAAAAGTTTATCGTGCTGTCTGATGGCTAAATTCAGATAGCACCTCGGAACCTCTGGTTTTTT
```

Section 5

```
              (537) 537          550       560       570       580       590       600       610       620       630       640       650       660      670
APOSTART 1    (112) ----------------------------------GTGCCGCTGAAGTCGCTGCTAATAGACGGGAACTGCAGGGTGGAGGATAGAGGGCTCAAAACACATCATGGCAA--------------------------
APOSTART 2    (112) ----------------------------------GTGCCGCTGAAGTCGCTGCTAATCGATGGGAACTGCAGGGTGGAGGATAGAGGGCTCAAAACACATCATGGCAA--------------------------
APOSTART_3    (112) ----------------------------------GTGCCGCTGAAGTCGCTGCTAATAGACGGGAACTGCAGGGTGGAGGATAGAGGGCTCAAAACACATCATGGCAA--------------------------
APOSTART_4    (112) ----------------------------------GTGCCGCTGAAGTCGCTGCTAATCGATGGGAATTGCAGGGTGGAGGATAGAGGGCTGAAAACACATCATGGCAA--------------------------
APOSTART_5    (112) ----------------------------------GTGCCTCTTAAGTCTCTGCTAATCGACGGGAACTGCAGGGTGGAGGACAGAGGACTGAAAACACATCATGGCAA--------------------------
APOSTART_6    (112) ----------------------------------GTGCCTCTAAAGTCGCTGCTAATCGACGGGAACTGCAGGGTGGAGGACAGAGGACTGAAAACACATCATGGCAA--------------------------
APOSTART 7    (112) ----------------------------------GTGCCGCTGAAGTCGCTGCTAATCGATGGGAATTGCAGGGTGGAGGATAGAGGGCTGAAAACACATCATGGCAA--------------------------
APOSTART_8    (112) ----------------------------------GTGCCGCTGAAGTCGCTGCTAATAGACGGGAACTGCAGGGTGGAGGATAGAGGGCTCAAAACACATCATGGCAA--------------------------
APOSTART_9    (112) ----------------------------------GTGCCGCTGAAGTCGCTGCTAATAGACGGGAACTGCAGGGTGGAGGATAGAGGGCTCAAAACACATCATGGCAA--------------------------
APOSTART_10   (112) ----------------------------------GTGCCGCTGAAGTCGCTGCTAATAGACGGGAACTGCAGGGTGGAGGATAGAGGGCTCAAAACACATCATGGCAA--------------------------
APOSTART_11   (112) ----------------------------------GTGCCGCTGAAGTCGCTGCTAATAGACGGGAACTGCAGGGTGGAGGATAGAGGGCTCAAAACACATCATGGCAA--------------------------
APOSTART_12   (112) ----------------------------------GTGCCGCTGAAGTCGCTGCTAATCGATGGGAATTGCAGGGTGGAGGATAGAGGGCTGAAAACACATCATGGCAA--------------------------
APOSTART_14   (112) ----------------------------------GTGCCGCTGAAGTCGCTGCTAATCGATGGGAATTGCAGGGTGGAGGATAGAGGGCTGAAAACACATCATGGCAA--------------------------
APOSTART_15   (112) ----------------------------------GTGCCGCTGAAGTCGCTGCTAATCGATGGGAATTGCAGGGTGGAGGATAGAGGGCTGAAAACACATCATGGCAA--------------------------
APOSTART_13   (112) ----------------------------------GTGCCGCTGAAGTCGCTGCTAATCGATGGGAATTGCAGGGTGGAGGATAGAGGGCTGAAAACACATCATGGCAA--------------------------
APOSTART_1_GEN (521) GTCAAGTGTTTATTAAGACTTG-TGTACATACAGGTGCCGCTGAAGTCGCTGCTAATAGACGGGAACTGCAGGGTGGAGGATAGAGGGCTCAAAACACATCATGGCAAGTGAGTCGGCTTTT---CCCTTTTC
APOSTART_2_GEN (524) GTCAAGTGTTTATTAAGACTTG-TGTACATACAGGTGCCGCTGAAGTCGCTGCTAATCGATGGGAACTGCAGGGTGGAGGATAGAGGGCTCAAAACACATCATGGCAAGTGAGTCGGCTTTT---CCCTTTTC
APOSTART_5_GEN (496) ----AGTTTTCATTAAGAAATG-TGTACATGCAGGTGCCTCTTAAGTCTCTGCTAATCGACGGGAACTGCAGGGTGGAGGACAGAGGACTGAAAACACATCATGGCAAGTGAGTTTGCTATTATTCTCTTTTC
APOSTART_6_GEN (442) GC--AATTTTCATTAAGAAATT-TGTACATGCAGGTGCCTCTAAAGTCGCTGCTAATCGACGGGAACTGCAGGGTGGAGGACAGAGGACTGAAAACACATCATGGCAAGTGAGTTTGCTATTATTCTCTTTTC
APOSTART_7_GEN (535) GTTAAGTGTTTATTAAGACTTTCTATACATACAGGTGCCGCTGAAGTCGCTGCTAATCGATGGGAATTGCAGGGTGGAGGATAGAGGGCTGAAAACACATCATGGCAAGTGAGTCTGCTTTT---CCCTTTTC
APOSTART_8_GEN (521) GTCAAGTGTTTATTAAGACTTG-TGTACATACAGGTGCCGCTGAAGTCGCTGCTAATAGACGGGAACTGCAGGGTGGAGGATAGAGGGCTCAAAACACATCATGGCAAGTGAGTCGGCTTTT---CCCTTTTC
APOSTART_10_GEN (521) GTCAAGTGTTTATTAAGACTTG-TGTACATACAGGTGCCGCTGAAGTCGCTGCTAATAGACGGGAACTGCAGGGTGGAGGATAGAGGGCTCAAAACACATCATGGCAAGTGAGTCGGCTTTT---CCCTTTTC
APOSTART_12_GEN (529) GTTAAGTGTTTATTAAGACTTTCTATACATACAGGTGCCGCTGAAGTCGCTGCTAATCGATGGGAATTGCAGGGTGGAGGATAGAGGGCTGAAAACACATCATGGCAAGTGAGTCTGCTTTT---CCCTTTTC
```

Section 6

```
              (671) 671      680       690       700       710       720       730       740       750       760       770       780       790        804
APOSTART 1    (187) ----------------------------------------------------------------------------------------------------------------------------------------
APOSTART 2    (187) ----------------------------------------------------------------------------------------------------------------------------------------
APOSTART_3    (187) ----------------------------------------------------------------------------------------------------------------------------------------
APOSTART_4    (187) ----------------------------------------------------------------------------------------------------------------------------------------
APOSTART_5    (187) ----------------------------------------------------------------------------------------------------------------------------------------
APOSTART_6    (187) ----------------------------------------------------------------------------------------------------------------------------------------
APOSTART 7    (187) ----------------------------------------------------------------------------------------------------------------------------------------
APOSTART_8    (187) ----------------------------------------------------------------------------------------------------------------------------------------
APOSTART_9    (187) ----------------------------------------------------------------------------------------------------------------------------------------
APOSTART_10   (187) ----------------------------------------------------------------------------------------------------------------------------------------
APOSTART_11   (187) ----------------------------------------------------------------------------------------------------------------------------------------
APOSTART_12   (187) ----------------------------------------------------------------------------------------------------------------------------------------
APOSTART_14   (187) ----------------------------------------------------------------------------------------------------------------------------------------
APOSTART_15   (187) ----------------------------------------------------------------------------------------------------------------------------------------
APOSTART_13   (187) ----------------------------------------------------------------------------------------------------------------------------------------
APOSTART_1_GEN (651) CGACAACACGAGCTTGCTCGCTGTTCCTTGTTTTGCATTCTGAATGTTGGGCGTCGAATTATCATGATGAAGCTGTCGAACTGTAGATGTTACTATATACTACTACAAGAATCTTAGTAGATGTTGTATTTGCT
APOSTART_2_GEN (654) CACCAACACGAGCTTGCTCGCTGTTCCTTGTTTTGCATTCTGAATGTTGGGCCTCAAATTATCTCGATGAAGCTGTCGAACTGTAGATGTTACTATATACAACTACCAGAATCTTAGTACATGTTGTATTTGCT
APOSTART_5_GEN (625) TGATAAGTGATAACACGACTAGTTTGCTTGCTGTTCCTTTTTTATATATTTGTTTGGATTCGCAATGCCG-GGTGTTGAATGATCTTGATGAGGGTGTCCAACAGTA--GATATTACTGCATCCTAGAGCAGGA
APOSTART_6_GEN (573) CGATAACACGAG-TAGTATGCTCGCTGTTCCGTTTTTATATATTTGTTTGGATTCGAATAGTGGGTGTTGAATGATCTTGATGAGGGTGTCGAACTGT---A-------GATGTTACTGCATCCTAAAGCAGGA
APOSTART_7_GEN (666) CGACAACATGAGCTTGCTCACTGTTCCTTGTTTTGCATTATGAATGTTGGGCATTGAATTATCTCGATGAAGCCGTCGAACTGTAGATGTTACTGTATCCCACTACAAGAATCTTAAGAGATGTTATATTTGCA
APOSTART_8_GEN (651) CGACAACACGAGCTTGCTCGCTGTTCCTTGTTTTGCATTCTGAATGTTGGGCGTCGAATTATCATGATGAAGCTGTCGAACTGTAGATGTTACTATATACTACTACAAGAATCTTAGTAGATGTTGTATTTGCT
APOSTART_10_GEN (651) CGACAACACGAGCTTGCTCGCTGTTCCTTGTTTTGCATTCTGAATGTTGGGCGTCGAATTATCATGATGAAGCTGTCGAACTGTAGATGTTACTATATACTACTACAAGAATCTTAGTAGATGTTGTATTTGCT
APOSTART_12_GEN (660) CGACAACATGAGCTTGCTCACTGTTCCTTGTTTTGCATTATGAATGTTGGGCATTGAATTATCTCGATGAAGCCGTCGAACTGTAGATGTTACTGTATCCCACTACAAGAATCTTAAGAGATGTTATATTTGCA
```

Section 7

```
                (805) 805 810 820 830 840 850 860 870 880 890 900 910 920 938
APOSTART 1      (187) --------------------------------------------------------------------------------------------------------------------------------------
APOSTART 2      (187) --------------------------------------------------------------------------------------------------------------------------------------
APOSTART_3      (187) --------------------------------------------------------------------------------------------------------------------------------------
APOSTART_4      (187) --------------------------------------------------------------------------------------------------------------------------------------
APOSTART_5      (187) --------------------------------------------------------------------------------------------------------------------------------------
APOSTART_6      (187) --------------------------------------------------------------------------------------------------------------------------------------
APOSTART 7      (187) --------------------------------------------------------------------------------------------------------------------------------------
APOSTART_8      (187) --------------------------------------------------------------------------------------------------------------------------------------
APOSTART_9      (187) --------------------------------------------------------------------------------------------------------------------------------------
APOSTART_10     (187) --------------------------------------------------------------------------------------------------------------------------------------
APOSTART_11     (187) --------------------------------------------------------------------------------------------------------------------------------------
APOSTART_12     (187) --------------------------------------------------------------------------------------------------------------------------------------
APOSTART_14     (187) --------------------------------------------------------------------------------------------------------------------------------------
APOSTART_15     (187) --------------------------------------------------------------------------------------------------------------------------------------
APOSTART_13     (187) --------------------------------------------------------------------------------------------------------------------------------------
APOSTART_1_GEN  (785) CTTTAAAAAAAAATACGATTTCCTTAATTTATCTGTAGCTTAACCAAGAATTGTTCTCAGTCGTACTGCATTTGTTTGGAACAATTGTATATCCTACGATTCTGACAGTGTTAAATACTTAA-TCTTCCATGCA
APOSTART_2_GEN  (788) CTTTTAAAAAA--TACGATTTCCTTAATTTATCTGTAGCTTAACCAAAAATTGTTCTCAGTCGTACTGCATTTGTTTGGAACATTTGTATATCCTACGATTCTGACAGTGTAAAATACTTAA-TCTTCCATGCA
APOSTART_5_GEN  (756) ATCCTAATAGTTTCTGGTTTTCCTTTTCATGTGAGAA-ATAATAGTTTTCTTCATTTATCCGTAGCTATCTTGATTCAGAATTGTCCTCATTCGTATGATACTGACAGTATAGAATACTTAGTTCCTCCATGCA
APOSTART_6_GEN  (696) ATCCTAATAGTTTTTGGTTTTCCTCTTCTGCGAGAAATAATAGTTTTCTTCATTTATCGGTAG--CTAGCTTGATTCAGAACTGTCCTCATTCCTATGATTCTGACAGTATAGAATACTTAGATCTTTCATGCA
APOSTART_7_GEN  (800) CTTTAAAAGAAA-TACGATTTCCTAAATTGATCTGTAGCTTAACCAAGAATTGTTCTCAGTCGTACTGCATTTGTTTGGAACAATTATATATCCTACGATTCTGACAGTGTAAAATACTTAAATCTTCCATGCA
APOSTART_8_GEN  (785) CTTTAAAAAAAAATACGATTTCCTTAATTTATCTGTAGCTTAACCAAGAATTGTTCTCAGTCGTACTGCATTTGTTTGGAACAATTGTATATCCTACGATTCTGACAGTGTTAAATACTTAA-TCTTCCATGCA
APOSTART_10_GEN (785) CTTTAAAAAAAAATACGATTTCCTTAATTTATCTGTAGCTTAACCAAGAATTGTTCTCAGTCGTACTGCATTTGTTTGGAACAATTGTATATCCTACGATTCTGACAGTGTTAAATACTTAA-TCTTCCATGCA
APOSTART_12_GEN (794) CTTTAAAAGAAA-TACGATTTCCTAAATTGATCTGTAGCTTAACCAAGAATTGTTCTCAGTCGTACTGCATTTGTTTGGAACAATTATATATCCTACGATTCTGACAGTGTAAAATACTTAAATCTTCCATGCA
```

Section 8

```
                (939) 939 950 960 970 980 990 1000 1010 1020 1030 1040 1050 1060 1072
APOSTART 1      (187) --------------------------------------------------------------------------------------------------------------------------------------
APOSTART 2      (187) --------------------------------------------------------------------------------------------------------------------------------------
APOSTART_3      (187) --------------------------------------------------------------------------------------------------------------------------------------
APOSTART_4      (187) --------------------------------------------------------------------------------------------------------------------------------------
APOSTART_5      (187) --------------------------------------------------------------------------------------------------------------------------------------
APOSTART_6      (187) --------------------------------------------------------------------------------------------------------------------------------------
APOSTART 7      (187) --------------------------------------------------------------------------------------------------------------------------------------
APOSTART_8      (187) --------------------------------------------------------------------------------------------------------------------------------------
APOSTART_9      (187) --------------------------------------------------------------------------------------------------------------------------------------
APOSTART_10     (187) --------------------------------------------------------------------------------------------------------------------------------------
APOSTART_11     (187) --------------------------------------------------------------------------------------------------------------------------------------
APOSTART_12     (187) --------------------------------------------------------------------------------------------------------------------------------------
APOSTART_14     (187) --------------------------------------------------------------------------------------------------------------------------------------
APOSTART_15     (187) --------------------------------------------------------------------------------------------------------------------------------------
APOSTART_13     (187) --------------------------------------------------------------------------------------------------------------------------------------
APOSTART_1_GEN  (918) CACA-TTATGATCACTTCTTCAGAAACATTCA-GTGTCCTTTTTTTTGGCAACATGAAGAGCTGATTTTTGTAATGATCTATGTAGCTTATACATGCATT-TTTTCCATGATCACCGTAATTTTCTTACCCTTT
APOSTART_2_GEN  (919) CACA-TTATGATCACTTCTTCAGAAACATTCA-GTGTCCTTTTATTTTGG--CA-------------------AC---ATGAAGAGCTTATACATGCATT-TTTTCCATGATCACCATAATGTTCTTACCCTTT
APOSTART_5_GEN  (889) CACAGTTACGTCGACTTCTTCAGAAACATTCAAGTGTTC--TTTTTTGGTAAAAGGAGCATCTGATTTTTGTAATGATCTATATG----------------TA---GCTTA-----------------------
APOSTART_6_GEN  (828) CACCGTTATGTTGACTTCTTCAGAATCATTCAAGTGTTCTTTTTTTTAGGCAAAGGAAGAGCTGATTTTCGTAATGATCTATATGTAGCTTATTTATACAGTTTTCCGTGATCAGTATAATATTCCTGCCCCCT
APOSTART_7_GEN  (933) CACA-TTATGATCACTTCTTCAGAAACATTCAGTGTTC--TTTTTTTGGCAACATGAAGAGCTGATTTTTTTAATGATCTATGTAGCTTATTCATGCATT-TTTTCCATGATCACCGTAATGTTCTTACCCTTT
APOSTART_8_GEN  (918) CACA-TTATGATCACTTCTTCAGAAACATTCA-GTGTCCTTTTTTTTGGCAACATGAAGAGCTGATTTTTGTAATGATCTATGTAGCTTATACATGCATT-TTTTCCATGATCACCGTAATTTTCTTACCCTTT
APOSTART_10_GEN (918) CACA-TTATGATCACTTCTTCAGAAACATTCA-GTGTCCTTTTTTTTGGCAACATGAAGAGCTGATTTTTGTAATGATCTATGTAGCTTATACATGCATT-TTTTCCATGATCACCGTAATTTTCTTACCCTTT
APOSTART_12_GEN (927) CACA-TTATGATCACTTCTTCAGAAACATTCA-GTGTTCTTTTTTT-GGCAACATGAAGAGCTGATTTTTTTAATGATCTATGTAGCTTATTCATGCATT-TTTTCCATGATCACCGTAATGTTCTTACCCTTT
```

Section 9

```
                  (1073) 1073   1080          1090          1100          1110          1120          1130          1140          1150          1160          1170          1180          1190                1206
APOSTART 1         (187) ---------------------------------------------------------ATGATTTATGTCCTGTGTGTTTATAACAAGAAAGAAAAGGAGCACCAAATCAC------------------------
APOSTART 2         (187) ---------------------------------------------------------ATGATTTATGTCCTGTGTGTTTATAACAAGAAAGAAAAGGAGCACCAAATCAC------------------------
APOSTART_3         (187) ---------------------------------------------------------ATGATTTATGTCCTGTGTGTTTATAACAAGAAAGAAAAGGAGCACCAAATCAC------------------------
APOSTART_4         (187) ---------------------------------------------------------ATGATTTATGTCCTGTGTGTTTATAACAAAAAAGAAAAGGAGCACCAAATCAC------------------------
APOSTART_5         (187) ---------------------------------------------------------AGCACCAAATCACGGTTTGTTCTAAAGCTGAGAGCCCTTCAACACTG--TCAC------------------------
APOSTART_6         (187) ---------------------------------------------------------ATGACTTATGTCCTGTGCGTTTATAACAAGAAAGAAAAGGAGCACCAAATCAC------------------------
APOSTART 7         (187) ---------------------------------------------------------ATGATTTATGTCCTGTGTGTTTATAACAAGAAAGAAAAGGAGCACCAAATCAC------------------------
APOSTART_8         (187) ---------------------------------------------------------ATGATTTATGTCCTGTGTGTTTATAACAAGAAAGAAAAGGAGCACCAAATCAC------------------------
APOSTART_9         (187) ---------------------------------------------------------ATGATTTATGTCCTGTGTGTTTATAACAAGAAAGAAAAGGAGCACCAAATCAC------------------------
APOSTART_10        (187) ---------------------------------------------------------ATGATTTATGTCCTGTGTGTTTATAACAAGAAAGAAAAGGAGCACCAAATCAC------------------------
APOSTART_11        (187) ---------------------------------------------------------ATGATTTATGTCCTGTGTGTTTATAACAAGAAAGAAAAGGAGCACCAAATCAC------------------------
APOSTART_12        (187) ---------------------------------------------------------ATGATTTATGTCCTGTGTGTTTATAACAAGAAAGAAAAGGAGCACCAAATCAC------------------------
APOSTART_14        (187) ---------------------------------------------------------ATGATTTATGTCCTGTGTGTTTATAACAAAAAAGAAAAGGAGCACCAAATCAC------------------------
APOSTART_15        (187) ---------------------------------------------------------ATGATTTATGTCCTGTGTGTTTATAACAAAAAAGAAAAGGAGCACCAAATCAC------------------------
APOSTART_13        (187) ---------------------------------------------------------ATGATTTATGTCCTGTGTGTTTATAACAAGAAAGAAAAGGAGCACCAAATCAC------------------------
APOSTART_1_GEN    (1049) ACATTTTCATATATTTGCTTTTCTGTTCTCATCCCTCCTTTTCT---ATATTTTCAGATGATTTATGTCCTGTGTGTTTATAACAAGAAAGAAAAGGAGCACCAAATCACG--GTTTGTTCTGAAGCTGAGAAC
APOSTART_2_GEN    (1026) ACATTTTCCTATATTTGCTTTTCTGTTCTCATCCCTCCTTTTCT---ATATTTTCAGATGATTTATGTCCTGTGTGTTTATAACAAGAAAGAAAAGGAGCACCAAATCACG--GTTTGTTCTAAAGCTGAGAAC
APOSTART_5_GEN     (979) ---TTTTGCTAAATTCG-------GTTATCATCGGCTCTTTCC----ATCTTATCAGAGCACCAAATCACGGTTTGTTCTAAAGCTGAGAGCCCTTCAACACTG--TCACTTTGTTTGTTCTAAAGCTGAGAGC
APOSTART_6_GEN     (962) TACATTTTCTAAATTCA--------TTCTCATCCCTCTTT-----CCATCTTATCAGATGACTTATGTCCTGTGCGTTTATAACAAGAAAGAAAAGGAGCACCAAATCACG--GTTTGTTCTAAAGCTGAGAGC
APOSTART_7_GEN    (1063) ACATTTTCCTATATTTGCTTTTCTGTTCTCATCCCTCCTTTTCAC---TATTTTCAGATGATTTATGTCCTGTGTGTTTATAACAAGAAAGAAAAGGAGCACCAAATCACG--GTTTGTTCTGAAGCTGAGAAC
APOSTART_8_GEN    (1049) ACATTTTCATATATTTGCTTTTCTGTTCTCATCCCTCCTTTTCTTCCATCTTATCAGATGATTTATGTCCTGTGTGTTTATAACAAGAAAGAAAAGGAGCACCAAATCACG--GTTTGTTCTAAAGCTGAGAGC
APOSTART_10_GEN   (1049) ACATTTTCATATATTTGCTTTTCTGTTCTCATCCCTCCTTTTCT---ATATTTTCAGATGATTTATGTCCTGTGTGTTTATAACAAGAAAGAAAAGGAGCACCAAATCACG--GTTTGTTCTGAAGCTGAGAAC
APOSTART_12_GEN   (1057) ACATTTTCCTATATTTGCTTTTCTGTTCTCATCCCTCCTTTTCAC---TATTTTCAGATGATTTATGTCCTGTGTGTTTATAACAAGAAAGAAAAGGAGCACCAAATCACG--GTTTGTTCTGAAGCTGAGAAC
```

Section 10

```
                  (1207) 1207         1220          1230          1240          1250          1260          1270          1280          1290          1300          1310          1320          1330          1340
APOSTART 1         (240) ------------------------------------------------------------------GATGGGTGCATATGACATTGAAGATGCGCTGGCTTGGAAAAAGAAGATAGAGCAGATCATCGATCAG-
APOSTART 2         (240) ------------------------------------------------------------------GATGGGTGCATATGACATTGAAGATGCGCTGGCTTGGAAAAAGAAGATAGAGCAGATCATTGATCAG-
APOSTART_3         (240) ------------------------------------------------------------------GATGGGTGCATATGACATTGAAGATGCGCTGGCTTGGAAAAAGAAGATAGAGCAGATCATCGATCAG-
APOSTART_4         (240) ------------------------------------------------------------------GATGGGTGCATATGACATTGAAGATGCGCTGGCTTGGAAAAAGAAGATAGAGCAGATCATCGATCAG-
APOSTART_5         (238) ---------------------------TT-------------------------------------TATGGGTGCATACGACATTGAAGATGCTCTGGCTTGGAAAAAGAAGATAGAGCAGATCATCGATCAG-
APOSTART_6         (240) ------------------------------------------------------------------GATGGGTGCATATGACATTGAAGATGCTCTAGCTTGGAAAAAGAAGATAGAGCAGATCATCGATCAG-
APOSTART 7         (240) ------------------------------------------------------------------GATGGGTGCATATGACATTGAAGATGCTCTGGCTTGGAAAAAGAAGATAGAACAGATCATCGATCAG-
APOSTART_8         (240) ------------------------------------------------------------------GATGGGTGCATATGACATTGAAGATGCGCTGGCTTGGAAAAAGAAGATAGAGCAGATCATCGATCAG-
APOSTART_9         (240) ------------------------------------------------------------------GATGGGTGCATATGACATTGAAGATGCGCTGGCTTGGAAAAAGAAGATAGAGCAGATCATCGATCAG-
APOSTART_10        (240) ------------------------------------------------------------------GATGGGTGCATATGACATTGAAGATGCGCTGGCTTGGAAAAAGAAGATAGAGCAGATCATCGATCAG-
APOSTART_11        (240) ------------------------------------------------------------------GATGGGTGCATATGACATTGAAGATGCGCTGGCTTGGAAAAAGAAGATAGAGCAGATCATCGATCAG-
APOSTART_12        (240) ------------------------------------------------------------------GATGGGTGCATATGACATTGAAGATGCGCTGGCTTGGAAAAAGAAGATAGAGCAGATCATCGATCAG-
APOSTART_14        (240) ------------------------------------------------------------------GATGGGTGCATATGACATTGAAGATGCGCTGGCTTGGAAAAAGAAGATAGAGCAGATCATCGATCAG-
APOSTART_15        (240) ------------------------------------------------------------------GATGGGTGCATATGACATTGAAGATGCGCTGGCTTGGAAAAAGAAGATAGAGCAGATCATCGATCAG-
APOSTART_13        (240) ------------------------------------------------------------------GATGGGTGCATATGACATTGAAGATGCGCTGGCTTGGAAAAAGAAGATAGAGCAGATCATCGATCAG-
APOSTART_1_GEN    (1178) CCAC---------ACTATCACTTTTTGTTTCATTCAAATTTATGCCATA-ATTAATTTCATTCTCAGATGGGTGCATATGACATTGAAGATGCGCTGGCTTGGAAAAAGAAGATAGAGCAGATCATCGATCAGG
APOSTART_2_GEN    (1155) CCA-C--------ACTATCACTTTTTGTTTCATTCAAATTTATGCCATA-ATTAATTTCATTCTCAGATGGGTGCATATGACATTGAAGATGCGCTGGCTTGGAAAAAGAAGATAGAGCAGATCATTGATCAGG
APOSTART_5_GEN    (1097) CCTTCAACACTGTCACTTTGTTCGTTATGTCATTCACATTTATGCCCTAGATAAATTTAATGCTCAGATGGGTGCATACGACATTGAAGATGCTCTGGCTTGGAAAAAGAAGATAGAGCAGATCATCGATCAGG
APOSTART_6_GEN    (1081) CCTTCAACACTGTCACTTTGTTCGTTATGTCATTCACATTTATGCCCTAGATAAATTTAATGCTCAGATGGGTGCATATGACATTGAAGATGCTCTAGCTTGGAAAAAGAAGATAGAGCAGATCATCGATCAGG
APOSTART_7_GEN    (1192) CCACA---------CTATCAGTTTTTGTTTCATTTGGATGTATGCCATA-ATTAATTTCATTCTCAGATGGGTGCATATGACATTGAAGATGCTCTGGCTTGGAAAAAGAAGATAGAACAGATCATCGATCAGG
APOSTART_8_GEN    (1181) CCTTCAACACTGTCACTTTGTTCGTTATGTCATTCACATTTATGCCCTAGATTAATTTCATTCTCAGATGGGTGCATATGACATTGAAGATGCGCTGGCTTGGAAAAAGAAGATAGAGCAGATCATCGATCAGG
APOSTART_10_GEN   (1178) CCA----------CACTATCACTTTTTGTTTCATTCAAATTTATGCCATAATTAATTTCATTCTCAGATGGGTGCATATGACATTGAAGATGCGCTGGCTTGGAAAAAGAAGATAGAGCAGATCATCGATCAGG
APOSTART_12_GEN   (1186) CCA----------CACTATCAGTTTTTGTTTCATTTGGATGTATGCCATAATTAATTTCATTCTCAGATGGGTGCATATGACATTGAAGATGCGCTGGCTTGGAAAAAGAAGATAGAGCAGATCATCGATCAGG
```

Section 11

```
                 (1341) 1341     1350          1360          1370          1380          1390          1400          1410          1420          1430          1440          1450          1460        1474
APOSTART 1        (307) --------------------------------------------------------------------------------------------------------------------------------------
APOSTART 2        (307) --------------------------------------------------------------------------------------------------------------------------------------
APOSTART_3        (307) --------------------------------------------------------------------------------------------------------------------------------------
APOSTART_4        (307) --------------------------------------------------------------------------------------------------------------------------------------
APOSTART_5        (307) --------------------------------------------------------------------------------------------------------------------------------------
APOSTART_6        (307) --------------------------------------------------------------------------------------------------------------------------------------
APOSTART 7        (307) --------------------------------------------------------------------------------------------------------------------------------------
APOSTART_8        (307) --------------------------------------------------------------------------------------------------------------------------------------
APOSTART_9        (307) --------------------------------------------------------------------------------------------------------------------------------------
APOSTART_10       (307) --------------------------------------------------------------------------------------------------------------------------------------
APOSTART_11       (307) --------------------------------------------------------------------------------------------------------------------------------------
APOSTART_12       (307) --------------------------------------------------------------------------------------------------------------------------------------
APOSTART_14       (307) --------------------------------------------------------------------------------------------------------------------------------------
APOSTART_15       (307) --------------------------------------------------------------------------------------------------------------------------------------
APOSTART_13       (307) --------------------------------------------------------------------------------------------------------------------------------------
APOSTART_1_GEN   (1302) TGCAGTAATG-ATTTGCTTCAGCGGAACTTGT-CAACAGTAAAATAATTTTAAATAAGATATCTGAGAGATGCTCAAATACG-----AGGCATTTATGTTCCATTTATTTCATGTTGGGTCCTGAAATAATCCC
APOSTART_2_GEN   (1279) TGCAGTAAGG-ATTTGCTTCAGCGGAACTTGT-CAACAGTAAAATAATTGTAAATAAGATATCTGAGAGATGCTCAAAT----C-GAGGCGTTTATGTTCCATTTATTTTCATGTTGGGTCCTGAAATAATCCC
APOSTART_5_GEN   (1231) TGCAGTAAGG-ATTAGCTTCAGTGGAACTTGT------------------------------------ACAACAGTAACACTTTTAA-------CTTACATCCCTGAAAAATGCT---------AAAAAAAAAA
APOSTART_6_GEN   (1215) TGCAGTAAGG-ATTAGCTTCAGTGGAACTTGT------------------------------------ACAACAGTAACACTTTTAAGTTTTAACTTACATCCCTGAGAAAATGCT---------AAAAAAAAT
APOSTART_7_GEN   (1316) TGCAGTAAGGGATTTTCTTCAGTGTAACTTGTACAACAGCAACACTTTCAAATAACATATATACTTGAAAATGCTAAAAAAAAAATGAGGCATTTGTGTCCCATTTATTTTCGTCTTCATCGTGAAATAATCGT
APOSTART_8_GEN   (1315) TGCAGTAATG-ATTTGCTTCAGCGGAACTTGT-CAACTACTTCGTGCTCGAGGGCAAGCTGCTCGCCTACTACAAGAAGAAGCCCAAGGACAGCATGGTGCCGCTGAAGTCATGTTGGGTCCTGAAATAATCCC
APOSTART_10_GEN  (1302) TGCAGTAATG-ATTTGCTTCAGCGGAACTTGT-CAACAGTAAAATAATTTTAAATAAGATATCTGAGAGATGCTCAAATACG-----AGGCATTTATGTTCCATTTATTTCATGTTGGGTCCTGAAATAATCCC
APOSTART_12_GEN  (1310) TGCAGTAATG-ATTTGCTTCAGCGGAACTTGT-CAACAGTAAAATAATTTTAAATAAGATATCTGAGAGATGCTCAAATACG-----AGGCATTTATGTTCCATTTATTTCATGTTGGGTCCTGAAATAATCCC
```

Section 12

```
                 (1475) 1475 1480          1490          1500          1510          1520          1530          1540          1550          1560          1570          1580          1590              1608
APOSTART 1        (307) --------------------------------------------------------------------------------------------------------------------------------------
APOSTART 2        (307) --------------------------------------------------------------------------------------------------------------------------------------
APOSTART_3        (307) --------------------------------------------------------------------------------------------------------------------------------------
APOSTART_4        (307) --------------------------------------------------------------------------------------------------------------------------------------
APOSTART_5        (307) --------------------------------------------------------------------------------------------------------------------------------------
APOSTART_6        (307) --------------------------------------------------------------------------------------------------------------------------------------
APOSTART 7        (307) --------------------------------------------------------------------------------------------------------------------------------------
APOSTART_8        (307) --------------------------------------------------------------------------------------------------------------------------------------
APOSTART_9        (307) --------------------------------------------------------------------------------------------------------------------------------------
APOSTART_10       (307) --------------------------------------------------------------------------------------------------------------------------------------
APOSTART_11       (307) --------------------------------------------------------------------------------------------------------------------------------------
APOSTART_12       (307) --------------------------------------------------------------------------------------------------------------------------------------
APOSTART_14       (307) --------------------------------------------------------------------------------------------------------------------------------------
APOSTART_15       (307) --------------------------------------------------------------------------------------------------------------------------------------
APOSTART_13       (307) --------------------------------------------------------------------------------------------------------------------------------------
APOSTART_1_GEN   (1429) ATAGTTTGACAATTAAACACTAGAATGTATTGATGTACGATAAATCCTAGTGGTAGGACTAGCTGAAATAGTTTGGACTGTGTAGTGAATTTTATAAAATTAATGG-AAAGCTCTCCATGTTGTTTCTGTCGGA
APOSTART_2_GEN   (1406) ATAGTTTGACAATTAAACACTAGAATGTATTGATGTATGATAAATCCTAGTGGTAGGACTAGCTGAAATAGTTTGGACTGTGTAGTGAATTATATAAAATTAGTGG-AAAACTCTCCATGTTGTTTCTGTCAGA
APOSTART_5_GEN   (1312) TGAGTTTGACAATTACACTGCAGGAAATCTTGATATACGATAAATCCTAGTGGTAGGACTAGTTGAAATGATCTGGATCATGTAGTGATTTCTATAGGATTAATGGGAAAATTCTCCATGTTGTTTCTGTCAAA
APOSTART_6_GEN   (1303) ATGAGTTGACAATTACACTGCAGGAAATCTTGATATACGATAAATCCTAGTGGTAGGACTAGTTGAAATGATCTGGATCATGTAGTGATTTCTATAGGATTAATGGGAAAATTCTCCATGTTGTTTCTTTCAAA
APOSTART_7_GEN   (1450) ATAGTTTGACAATTACACAGCAGGAACTCTTGATATACGATAAA-----GTGGTAGGACTAATTGAAATAATATGGATCATGTAGTGAATTTTA--AATCTAATAGTATAACTCTCCATGTTATTTCTATCAAA
APOSTART_8_GEN   (1447) ATAGTTTGACAATTAAACACTAGAATGTATTGATGTACGATAAATCCTAGTGGTAGGACTAGCTGAAATAGTTTGGACTGTGTAGTGAATTTTATAAAATTAATGG-AAAGCTCTCCATGTTGTTTCTGTCGGA
APOSTART_10_GEN  (1429) ATAGTTTGACAATTAAACACTAGAATGTATTGATGTACGATAAATCCTAGTGGTAGGACTAGCTGAAATAGTTTGGACTGTGTAGTGAATTTTATAAAATTAATGG-AAAGCTCTCCATGTTGTTTCTGTCGGA
APOSTART_12_GEN  (1437) ATAGTTTGACAATTAAACACTAGAATGTATTGATGTACGATAAATCCTAGTGGTAGGACTAGCTGAAATAGTTTGGACTGTGTAGTGAATTTTATAAAATTAATGG-AAAGCTCTCCATGTTGTTTCTATCAAA
```

Section 13

```
                  (1609) 1609       1620      1630      1640      1650      1660      1670      1680      1690      1700      1710      1720      1730   1742
APOSTART 1        (307)  ---------------------------CAGGACACTATGACAGCTGAAAACCGCAAAGCCTTTGCTTCAATGGACTTCGATGCGGAACTTGGAGGGCAGTTCTCATTCTCAGATCATGACAGCGCG--------
APOSTART 2        (307)  ---------------------------CAGGACATTATGACAGCTAAAAACCGCAAAGCCTTTGCTTCAATGGACTTCGATGCGGAACTTGGAGGGCAGTTCTCATTCTCAGATCATGACAGCGCG--------
APOSTART_3        (307)  ---------------------------CAGGACACTATGACAGCTGAAAACCGCAAAGCCTTTGCTTCAATGGACTTCGATGCGGAACTTGGAGGGCAGTTCTCATTCTCAGATCATGACAGCGCG--------
APOSTART_4        (307)  ------------------------CAGCAGGACACTATGACAGCTAAAAACCGGAAAGCCTTTGCTTCGATGGACTTCGATGCGGAACTTGGAGGGCAGTTCTCATTCTCAGATCATGACAGCGCT--------
APOSTART_5        (307)  ---------------------------CAGGACACTATGACAGCTAAAAACCGCAAGGCCTTTGCTTCAATGGACTTCGATGCGGAACTTGGAGGGCAGTTCTCATTCTCAGATCATGACAGCGGG--------
APOSTART_6        (307)  ---------------------------CAGGACACTATGACAGCTAAAAACCGCAAGGCCTTTGCTTCAATGGACTTCGATGCGGAACTTGGAGGGCAGTTCTCATTCTCAGATCATGACAGCGGG--------
APOSTART 7        (307)  ------------------------CAGCAGGACACTATGACTGCTAAAAACCGCAAGGCCTTTGCTTCAATGGACTTCGATGCGGAACTTGGAGGGCAGTTCTCATTCTCAGATCATGACAGCGCG--------
APOSTART_8        (307)  ------------------------CAGCAGGACACTATGACAGCTGAAAACCGCAAAGCCTTTGCTTCAATGGACTTCGATGCGGAACTTGGAGGGCAGTTCTCATTCTCAGATCATGACAGCGCG--------
APOSTART_9        (307)  ---------------------------CAGGACACTATGACAGCTGAAAACCGCAAAGCCTTTGCTTCAATGGACTTCGATGCGGAACTTGGAGGGCAGTTCTCATTCTCAGATCATGACAGCGCG--------
APOSTART_10       (307)  ------------------------CAGCAGGACACTATGACAGCTGAAAACCGCAAAGCCTTTGCTTCAATGGACTTCGATGCGGAACTTGGAGGGCAGTTCTCATTCTCAGATCATGACAGCGCG--------
APOSTART_11       (307)  ------------------------------GACACTATGACAGCTGAAAACCGCAAAGCCTTTGCTTCAATGGACTTCGATGCGGAACTTGGAGGGCAGTTCTCATTCTCAGATCATGACAGCGCG--------
APOSTART_12       (307)  ------------------------CAGCAGGACACTATGACAGCTAAAAACCGCAAAGCCTTTGCTTCGATGGACTTCGATGCGGAACTTGGAGGGCAGTTCTTATTCTCAGATCATGACAGCGCT--------
APOSTART_14       (307)  ---------------------------CAGGACACTATGACAGCTAAAAACCGGAAAGCCTTTGCTTCGATGGACTTCGATGCGGAACTTGGAGGGCAGTTCTCATTCTCAGATCATGACAGCGCT--------
APOSTART_15       (307)  ------------------------------GACACTATGACAGCTAAAAACCGGAAAGCCTTTGCTTCGATGGACTTCGATGCGGAACTTGGAGGGCAGTTCTCATTCTCAGATCATGACAGCGCT--------
APOSTART_13       (307)  ---------------------------CAGGACACTATGACAGCTAAAAACCGCAAAGCCTTTGCTTCGATGGACTTCGATGCGGAACTTGGAGGGCAGTTCTCATTCTCAGATCATGACAGCGCT--------
APOSTART_1_GEN    (1562) TATCACATCTCTACATTCTTGCAGCAGCAGGACACTATGACAGCTGAAAACCGCAAAGCCTTTGCTTCAATGGACTTCGATGCGGAACTTGGAGGGCAGTTCTCATTCTCAGATCATGACAGCGCGTATGTGAC
APOSTART_2_GEN    (1539) TATCACATCTCTACATTCTTGCAGCAGCAGGACATTATGACAGCTAAAAACCGCAAAGCCTTTGCTTCAATGGACTTCGATGCGGAACTTGGAGGGCAGTTCTCATTCTCAGATCATGACAGCGCGTATGTGAC
APOSTART_5_GEN    (1446) TATCACATATCTACATTCTTGCAGCAGCAGGACACTATGACAGCTAAAAACCGCAAGGCCTTTGCTTCAATGGACTTCGATGCGGAACTTGGAGGGCAGTTCTCATTCTCAGATCATGACAGCGGGTATGTAAT
APOSTART_6_GEN    (1437) TATCACATATCTACATTCTTGCAGCAGCAGGACACTATGACAGCTAAAAACCGCAAGGCCTTTGCTTCAATGGACTTCGATGCGGAACTTGGAGGGCAGTTCTCATTCTCAGATCATGACAGCGGGTATGTAAT
APOSTART_7_GEN    (1577) TATCACGTCTCTACATTCTTGCAGCAGCAGGACACTATGACTGCTAAAAACCGCAAGGCCTTTGCTTCAATGGACTTCGATGCGGAACTTGGAGGGCAGTTCTCATTCTCAGATCATGACAGCGCGTATGTGAC
APOSTART_8_GEN    (1580) TATCACATCTCTACATTCTTGCAGCAGCAGGACACTATGACAGCTGAAAACCGCAAAGCCTTTGCTTCAATGGACTTCGATGCGGAACTTGGAGGGCAGTTCTCATTCTCAGATCATGACAGCGCGTATGTGAC
APOSTART_10_GEN   (1562) TATCACATCTCTACATTCTTGCAGCAGCAGGACACTATGACAGCTGAAAACCGCAAAGCCTTTGCTTCAATGGACTTCGATGCGGAACTTGGAGGGCAGTTCTCATTCTCAGATCATGACAGCGCGTATGTGAC
APOSTART_12_GEN   (1570) TATCACGTCTCTACATTCTTGCAGCAGCAGGACACTATGACAGCTAAAAACCGCAAAGCCTTTGCTTCGATGGACTTCGATGCGGAACTTGGAGGGCAGTTCTTATTCTCAGATCATGACAGCGCGTATGTGAC
```

Section 14

```
                  (1743) 1743   1750      1760      1770      1780      1790      1800      1810      1820      1830      1840      1850      1860      1876
APOSTART 1        (406)  -----------------------------------------------------------------------GCTGAGGACGAAGAAGAGCGGCCCACTTTGACCCGTAGGACAACTATAGGAAACGGT------
APOSTART 2        (406)  -----------------------------------------------------------------------GCTGAGGACGAAGAAGAGCGGCCCACTTTGACCCGCAGGACAACTATAGGAAACGGT------
APOSTART_3        (406)  -----------------------------------------------------------------------GCTGAGGACGAAGAAGAGCGGCCCACTTTGACCCGTAGGACAACTATAGGAAACGGT------
APOSTART_4        (409)  -----------------------------------------------------------------------GCCGAGGACGAAGAGGAGCGACCCACTTTGACCCGTAGGACAACTATAGGAAACGGT------
APOSTART_5        (406)  -----------------------------------------------------------------------GCTGAAGACGAAGAGGAACGGCCGACTTTGACACGTAGGACAACTATAGGAAACGGT------
APOSTART_6        (406)  -----------------------------------------------------------------------GCTGAAGACGAAGAGGAACGGCCGACTTTGACACGTAGGACAACTATAGGAAACGGT------
APOSTART 7        (409)  -----------------------------------------------------------------------GCTGAAGACGAAGAAGAGCGACCCACTTTGACCCGTAGGACAACTATAGGAAACGGT------
APOSTART_8        (409)  -----------------------------------------------------------------------GCTGAGGACGAAGAAGAGCGGCCCACTTTGACCCGTAGGACAACTATAGGAAACGGT------
APOSTART_9        (406)  -----------------------------------------------------------------------GCTGAGGACGAAGAAGAGCGGCCCACTTTGACCCGTAGGACAACTATAGGAAACGGT------
APOSTART_10       (409)  -----------------------------------------------------------------------GCTGAGGACGAAGAAGAGCGGCCCACTTTGACCCGTAGGACAACTATAGGAAACGGT------
APOSTART_11       (403)  -----------------------------------------------------------------------GCTGAGGACGAAGAAGAGCGGCCCACTTTGACCCGTAGGACAACTATAGGAAACGGT------
APOSTART_12       (409)  -----------------------------------------------------------------------GCTGAGGACGAAGAGGAGCGACCCACTTTGACCCGTAGGACAACTATAGGAAACGGT------
APOSTART_14       (406)  -----------------------------------------------------------------------GCCGAGGACGAAGAGGAGCGACCCACTTTGACCCGTAGGACAACTATAGGAAACGGT------
APOSTART_15       (403)  -----------------------------------------------------------------------GCCGAGGACGAAGAGGAGCGACCCACTTTGACCCGTAGGACAACTATAGGAAACGGT------
APOSTART_13       (406)  -----------------------------------------------------------------------GCTGAGGACGAAGAGGAGCGACCCACTTTGACCCGTAGGACAACTATAGGAAACGGT------
APOSTART_1_GEN    (1696) ATTCTACTCTTAGACTATCTTGTACCTAAGTGTTTCGCTGAATTAAGTACAAATTTAATTACCAATGCAGGGCTGAGGACGAAGAAGAGCGGCCCACTTTGACCCGTAGGACAACTATAGGAAACGGTAAGAAG
APOSTART_2_GEN    (1673) ATTCTACTCTTAGACTATCTTGTACGTAAGTGTTTCGCTGAATTAAGTACAAATTTAATTACCAATGTAGGGCTGAGGACGAAGAAGAGCGGCCCACTTTGACCCGCAGGACAACTATAGGAAACGGTAAGAAG
APOSTART_5_GEN    (1580) ATT--------AGACTATCTTATACCTAAACCTTTCGCTAAATTAATTAGAAATTGAATTGCATATGTAGGGCTGAAGACGAAGAGGAACGGCCGACTTTGACACGTAGGACAACTATAGGAAACGGTAAGAAG
APOSTART_6_GEN    (1571) AT--T------AGACTATCTTATACCTAAACCTTTTGCTAAATTAACTAGAAATTGAATTGCATATGTAGGGCTGAAGACGAAGAGGAACGGCCGACTTTGACACGTAGGACAACTATAGGAAACGGTAAGAAG
APOSTART_7_GEN    (1711) ATTCTACTCTTAGACTATCTTATACCTAAACGTTTCACTGAATTAACTAGAAATTTAATTACCAATGCAGGGCTGAAGACGAAGAAGAGCGACCCACTTTGACCCGTAGGACAACTATAGGAAACGGTAAGAAG
APOSTART_8_GEN    (1714) ATTCTACTCTTAGACTATCTTATACCTAAACCTTTCGCTAAATTAATTAGAAATTGAATTGCATATGTAGGGCTGAGGACGAAGAAGAGCGGCCCACTTTGACCCGTAGGACAACTATAGGAAACGGTAAGAAG
APOSTART_10_GEN   (1696) ATTCTACTCTTAGACTATCTTGTACCTAAGTGTTTCGCTGAATTAAGTACAAATTTAATTCCA-ATGCAGGGCTGAGGACGAAGAAGAGCGGCCCACTTTGACCCGTAGGACAACTATAGGAAACGGTAAGAAG
APOSTART_12_GEN   (1704) ATTCTACTCTTAGACTATCTTATACCTAAACGTTTCACTGAATTAACTAGAAATTTAATTACCAATGCAGTGCTGAGGACGAAGAGGAGCGACCCACTTTGACCCGTAGGACAACTATAGGAAACGGTAAGAAG
```

```
                                                                                                                                                                              Section 15
               (1877) 1877         1890           1900           1910           1920           1930           1940           1950           1960           1970           1980           1990           2000           2010
APOSTART_1     (463)  --------------------------------------------------------------------------------------------------------CCCCCCGAATCGATACATGACTGGACCAAT
APOSTART_2     (463)  --------------------------------------------------------------------------------------------------------CCCCCCGAATCGATACATGACTGGACAAAT
APOSTART_3     (463)  --------------------------------------------------------------------------------------------------------CCCCCCGAATCGATACATGACTGGACCAAT
APOSTART_4     (466)  --------------------------------------------------------------------------------------------------------CCCCCCGAATCGATACATGACTGGACCAAT
APOSTART_5     (463)  --------------------------------------------------------------------------------------------------------CCCCCCGAATCCATACATGATTGGACCTAT
APOSTART_6     (463)  --------------------------------------------------------------------------------------------------------CCCCCCGAATCCATACATGATTGGACCAAT
APOSTART_7     (466)  --------------------------------------------------------------------------------------------------------CCCCCCGAATCGATACATGACTGGACCAAT
APOSTART_8     (466)  --------------------------------------------------------------------------------------------------------CCCCCCGAATCGATACATGACTGGACCAAT
APOSTART_9     (463)  --------------------------------------------------------------------------------------------------------CCCCCCGAATCGATACATGACTGGACCAAT
APOSTART_10    (466)  --------------------------------------------------------------------------------------------------------CCCCCCGAATCGATACATGACTGGACCAAT
APOSTART_11    (460)  --------------------------------------------------------------------------------------------------------CCCCCCGAATCGATACATGACTGGACCAAT
APOSTART_12    (466)  --------------------------------------------------------------------------------------------------------CCCCCCGAATCGATACATGACTGGACCAAT
APOSTART_14    (463)  --------------------------------------------------------------------------------------------------------CCCCCCGAATCGATACATGACTGGACCAAT
APOSTART_15    (460)  --------------------------------------------------------------------------------------------------------CCCCCCGAATCGATACATGACTGGACCAAT
APOSTART_13    (463)  --------------------------------------------------------------------------------------------------------CCCCCCGAATCGATACATGACTGGACCAAT
APOSTART_1_GEN (1830) TTGCACTGACTACCTGAGTGAAGAAGAGTACTGATTTGCAAGTTGGATTTTCATCTT-----TGCATTCTACATTAAATTCACCGTGTCTTTCTGGTGCTAGGTCCCCCCGAATCGATACATGACTGGACCAAT
APOSTART_2_GEN (1807) TTGCACTGACTACCTGAGTGAAGAATAGTACTGATTTGCAAGTTGGATTTTCATCTT-----TGCATTCTACATAAAATTCACCGTGTCTTTCTGGTGCTAGGTCCCCCCGAATCGATACATGACTGGACAAAT
APOSTART_5_GEN (1706) TTACACCGACTACCCGAGTGAAGCAGAGGATTGATTTGCATGTTGGATTTTGCATTTTTGCATGCTACATAAATAAAACTCAGCATGTCTTTATGGTGCTAGGTCCCCCCGAATCCATACATGATTGGACCTAT
APOSTART_6_GEN (1697) TTACACCGACTACCCGAGTGAAGCAGAGGATTGATTTGCATGTTGGATTTTGCATATTTGCATGCTACATAAATAAAACTCAGCGTGTCTTTATGGTGCTAGGTCCCCCCGAATCCATACATGATTGGACCAAT
APOSTART_7_GEN (1845) TTGCACTGACTACCCGAGTGAAGAAGAGTACTGATTTGCAAGTTGGATTTTCATCTT-----TGCATTCTAAATAAAATTCACCATGTCTTTCTGGTGCTAGGTCCCCCCGAATCGATACATGACTGGACCAAT
APOSTART_8_GEN (1848) TTACACCGACTACCCGAGTGAAGCAGAGGATTGATTTGCATGTTGGATTTTGCATTTTTGCATGCTACATAAATAAAACTCAGCATGTCTTTATGGTGCTAGGTCCCCCCGAATCGATACATGACTGGACCAAT
APOSTART_10_GEN (1829) TTGCACTGACTACCTGAGTGAAGAAGAGTACTGATTTGCAAAGTTGGATTTTCATCTTTGCATTCTCATTAAATTCACCGTGTC-----TTTC-TGGGCTAGGTCCCCCCGAATCGATACATGACTGGACCAAT
APOSTART_12_GEN (1838) TTGCACTGACTACCCGAGTGAAGAAGAGTACTGATTTGCAAGTTGGATTTTCATCTT-----TGCATTCTAAATAAAATTCACCATGTCTTTCTGGTGCTAGGTCCCCCCGAATCGATACATGACTGGACCAAT

                                                                                                                                                                              Section 16
               (2011) 2011           2020           2030           2040           2050           2060           2070           2080           2090           2100           2110           2120           2130           2144
APOSTART_1     (493)  GAACCTGATATCGGTTTGTCAAATCAAAGTGATCCTGCACAATCATTCTCAAAGAAGAATTGGCGGCTACTTCGATGCCAAAATGG------------------------------------------------
APOSTART_2     (493)  GAGCCTGATATCGGTTTGTCAAATCAGAGTGATCCTGCACAATCGTTCTCAAAGAAGAATTGGCGGCTACTTCGATGCCAAAATGG------------------------------------------------
APOSTART_3     (493)  GAACCTGATATCGGTTTGTCAAATCAAAGTGATCCTGCACAATCATTCTCAAAGAAGAATTGGCGGCTACTTCGATGCCAAAATGG------------------------------------------------
APOSTART_4     (496)  GAGCCTGATATCGGTTTGTCAAATCAGAGTGATCCTGCACAGTCTTTCTCAAAGAAGAATTGGCGGCTACTTCGATGCCAAAATGG------------------------------------------------
APOSTART_5     (493)  GAGCCTGATATTGGTTTGTCGAGTCAGAGTGACCCTTCACAATCTTTCTCGAAAAAGAATTGGCGGCTACTTAGATGCCAAAATGG------------------------------------------------
APOSTART_6     (493)  GAGCCTGATATTGGTTTGTCGAGTCAGAGTGACCCTTCACAATCTTTCTCGAAAAAGAATTGGCGGCTACTTAGATGCCAAAATGG------------------------------------------------
APOSTART_7     (496)  GAACCTGATATCGGTTTGACAAATCAGAGTGATCCTGCACAGTCTTTCTCAAAGAAGAATTGGCGGCTACTTCGATGCCAAAATGG------------------------------------------------
APOSTART_8     (496)  GAACCTGATATCGGTTTGTCAAATCAAAGTGATCCTGCACAATCATTCTCAAAGAAGAATTGGCGGCTACTTCGATGCCAAAATGG------------------------------------------------
APOSTART_9     (493)  GAACCTGATATCGGTTTGTCAAATCAAAGTGATCCTGCACAATCATTCTCAAAGAAGAATTGGCGGCTACTTCGATGCCAAAATGG------------------------------------------------
APOSTART_10    (496)  GAACCTGATATCGGTTTGTCAAATCAAAGTGATCCTGCACAATCATTCTCAAAGAAGAATTGGCGGCTACTTCGATGCCAAAATG-------------------------------------------------
APOSTART_11    (490)  GAACCTGATATCGGTTTGTCAAATCAAAGTGATCCTGCACAATCATTCTCAAAGAAGAATTGGCGGCTACTTCGATGCCAAAATGG------------------------------------------------
APOSTART_12    (496)  GAGCCTGATATCGGTTTGTCAAATCAGAGTGATCCTGCACAGTCTTTCTCAAAGAAGAATTGGCGGCTACTTCGATGCCAAAATGG------------------------------------------------
APOSTART_14    (493)  GAGCCTGATATCGGTTTGTCAAATCAGAGTGATCCTGCACAGTCTTTCTCAAAGAAGAATTGGCGGCTACTTCGATGCCAAAATGG------------------------------------------------
APOSTART_15    (490)  GAGCCTGATATCGGTTTGTCAAATCAGAGTGATCCTGCACAGTCTTTCTCAAAGAAGAATTGGCGGCTACTTCGATGCCAAAATGGCAAGCCAAAGTTCCTTATCCTTATGTCATCTACCCT-----TGCTTG-
APOSTART_13    (493)  GAGCCTGATATCGGTTTGTCAAATCAGAGTGATCCTGCACAGTCTTTCTCAAAGAAGAATTGGCGGCTACTTCGATGCCAAAATGG------------------------------------------------
APOSTART_1_GEN (1959) GAACCTGATATCGGTTTGTCAAATCAAAGTGATCCTGCACAATCATTCTCAAAGAAGAATTGGCGGCTACTTCGATGCCAAAATGGCAAGCCAAAGTTCCTTCTCCTTATGTCATCTACCCT-----TGCATGT
APOSTART_2_GEN (1936) GAGCCTGATATCGGTTTGTCAAATCAGAGTGATCCTGCACAATCGTTCTCAAAGAAGAATTGGCGGCTACTTCGATGCCAAAATGGCAAGCCAAAGTTCCTTCTCCTTATGTCATCTACCCT-----TGCATGT
APOSTART_5_GEN (1840) GAGCCTGATATTGGTTTGTCGAGTCAGAGTGACCCTTCACAATCTTTCTCGAAAAAGAATTGGCGGCTACTTAGATGCCAAAATGGCAAGCCCAACTTCCCTTACCTTATGTCATCCACCCTAACCTTGTATAT
APOSTART_6_GEN (1831) GAGCCTGATATTGGTTTGTCGAGTCAGAGTGACCCTTCACAATCTTTCTCGAAAAAGAATTGGCGGCTACTTAGATGCCAAAATGGCAAGCCCAACTTCCCTTACCTTATGTCATCCACCCTAACCTTGTATAT
APOSTART_7_GEN (1974) GAACCTGATATCGGTTTGACAAATCAGAGTGATCCTGCACAGTCTTTCTCAAAGAAGAATTGGCGGCTACTTCGATGCCAAAATGGCAAGCCAAAGTTC-TTCTCCTTATGTCATCTACCCT-----TGCATGT
APOSTART_8_GEN (1982) GAACCTGATATCGGTTTGTCAAATCAAAGTGATCCTGCACAATCATTCTCAAAGAAGAATTGGCGGCTACTTCGATGCCAAAATGGCAAGCCAAAGTTCCTTCTCCTTATGTCATCTACCCT-----TGCATGT
APOSTART_10_GEN (1957) GAACCTGATATCGGTTTGTCAAATCAAAGTGATCCTGCACAATCATTCTCAAAGAAGAATTGGCGGCTACTTCGATGCCAAAATGGCAAGCCAAAGTTCCTTCTCCTTATGTCATCTACCCT-----TGCATGT
APOSTART_12_GEN (1967) GAGCCTGATATCGGTTTGTCAAATCAGAGTGATCCTGCACAGTCTTTCTCAAAGAAGAATTGGCGGCTACTTCGATGCCAAAATGGCAAGCCAAAGTTCCTTCTCCTTATGTCATCTACCCT-----TGCATGT
```

Section 17

```
                  (2145) 2145 2150      2160      2170      2180      2190      2200      2210      2220      2230      2240      2250      2260           2278
      APOSTART 1   (579) -------------------------------ACTTCGCATCTTCGAAGAACTTCTGGAAGTCGATTACCTTG--------------------------------------------------------------
      APOSTART 2   (579) -------------------------------ACTTCGCATCTTCGAAGAACTTCTGGAAGTCGATTACCTTG--------------------------------------------------------------
      APOSTART_3   (579) -------------------------------ACTTCGCATCTTCGAAGAACTTCTGGAAGTCGATTACCTTG--------------------------------------------------------------
      APOSTART_4   (582) -------------------------------ACTTCGCATCTTCGAAGAACTTCTGGAAGTCGATTACCTCG--------------------------------------------------------------
      APOSTART_5   (579) -------------------------------ACTACGCATCTTTGAAGAACTTCTGGAAGTCGATTATCTTG--------------------------------------------------------------
      APOSTART_6   (579) -------------------------------ACTACGCATCTTTGAAGAACTTCTGGAAGTCGATTATCTTG--------------------------------------------------------------
      APOSTART 7   (582) -------------------------------ACTTCGCATCTTCGAAGAACTTCTGGAAGTCGATTACCTTG--------------------------------------------------------------
      APOSTART_8   (582) -------------------------------ACTTCGCATCTTCGAAGAACTTCTGGAAGTCGATTACCTTG--------------------------------------------------------------
      APOSTART_9   (579) -------------------------------ACTTCGCATCTTCGAAGAACTTCTGGAAGTCGATTACCTTG--------------------------------------------------------------
     APOSTART_10   (581) ------------------------------------------------AACTTCTGGAAGTCGATTACCTTG--------------------------------------------------------------
     APOSTART_11   (576) -------------------------------ACTTCGCATCTTCGAAGAACTTCTGGAAGTCGATTACCTTG--------------------------------------------------------------
     APOSTART_12   (582) -------------------------------ACTTCGCATCTTCGAAGAACTTCTGGAAGTCGATTACCTCG--------------------------------------------------------------
     APOSTART_14   (579) -------------------------------ACTTCGCATCTTCGAAGAACTTCTGGAAGTCGATTACCTCG--------------------------------------------------------------
     APOSTART_15   (618) GGAGTTGATTATCTCTTT-GTCATTAACAGGACTTCGCATCTTCGAAGAACTTCTGGAAGTCGATTACCTCG--------------------------------------------------------------
     APOSTART_13   (579) -------------------------------ACTTCGCATCTTCGAAGAACTTCTGGAAGTCGATTACCTCG--------------------------------------------------------------
 APOSTART_1_GEN   (2088) GGAGTTGCTTATCTCTTT-GTCATTAACAGGACTTCGCATCTTCGAAGAACTTCTGGAAGTCGATTACCTTGTATGTCCCGGCAGCAACCTTAACTGCTGGAGAGTTCCTGGAGAGTACCTGAAATTCTGAATC
 APOSTART_2_GEN   (2065) GGAGTTGCTTATCTCTTT-GTCATTAACAGGACTTCGCATCTTCGAAGAACTTCTGGAAGTCGATTACCTTGTATGTCCCAGCAGCAACCTTAACTGCTGGAGAGTTCCTGGACAGTACCTGAAATTCTGAATC
 APOSTART_5_GEN   (1974) GGAGTTGCTTACCTTTTTTGCCATTGGCAGGACTACGCATCTTTGAAGAACTTCTGGAAGTCGATTATCTTGTATGTCCCGG-----ATCTTAACTGTT-----------GACAAGTGCCTGAAATTCAGAAAC
 APOSTART_6_GEN   (1965) GTAGTTGCTTACCTTTTTTGCCATTGGCAGGACTACGCATCTTTGAAGAACTTCTGGAAGTCGATTATCTTGTATGTCCCGG-----ATCTTAACTGTT-----------GACAAGTGCCTGAAATTCAGAAAC
 APOSTART_7_GEN   (2102) GGAGTTGCTTATATCTTT-GTCATTAACAGGACTTCGCATCTTCGAAGAACTTCTGGAAGTCGATTACCTTGTATGTCCCGGCAGCAACCTTAACTGCTCGAGAGTTCCTGGACAGTACCTGAAATTCTGAATC
 APOSTART_8_GEN   (2111) GGAGTTGCTTATATCTTT-GTCATTAACAGGACTTCGCATCTTCGAAGAACTTCTGGAAGTCGATTACCTTGTATGTCCCGGCAGCAACCTTAACTGCTCGAGAGTTCCTGGACAGTACCTGAAATTCTGAATC
APOSTART_10_GEN   (2086) GGAGTTGCTTATCTCTTT-GTCATTAACAGGACTTCGCATCTTCGAAGAACTTCTGGAAGTCGATTACCTTGTATGTCCCGGCAGCAACCTTAACTGCTGGAGAGTTCCTGGAGAGTACCTGAAATTCTGAATC
APOSTART_12_GEN   (2096) GGAGTTGCTTATCTCTTT-GTCATTAACAGGACTTCGCATCTTCGAAGAACTTCTGGAAGTCGATTACCTCGTATGTCCCGGCAGCAACCTTAACTGCTGGAGAGTTCCTGGACAATACCTGAAATTCTGAATC
```

Section 18

```
                  (2279) 2279       2290      2300      2310      2320      2330      2340      2350      2360      2370      2380      2390      2400        2412
      APOSTART 1   (620) -----------------------------------CACGAAGCTGTAGCCGAGCTATGAGGGCTGTCGGCGTAGTGGAAGCCACATGCGAAGCCATTTTTGGGCTGGTGATGAGCATGGACATGACGAGATACG
      APOSTART 2   (620) -----------------------------------CACGAAACTGTAGCCGAGCTATGAGGGCTGTCGGCGTAATGGAAGCCACATGCGAAGCCATTTTTGGGCTGGTGATGAGCATGGACGTGACGAGATACG
      APOSTART_3   (620) -----------------------------------CACGAAGCTGTAGCCGAGCTATGAGGGCTGTCGGCGTAGTGGAAGCCACATGCGAAGCCATTTTTGGGCTGGTGATGAGCATGGACATGACGAGATACG
      APOSTART_4   (623) -----------------------------------CACGAAGCTGTAGCCGAGCTATGAGGGCTGTCGGCGTAGTGGAAGCCACATGCGAAGCCATTTTTGGGCTGGTGATGAGCATGGACGTGACGAGATACG
      APOSTART_5   (620) -----------------------------------CACGAAGCTGTAGCCGAGCTATGAGGGCTGTCGGCGTGGTGGAGGCCACATGTGAAGCCATTTTTGGGCTGGTGATGAGCATGGATGTGACTAGATACC
      APOSTART_6   (620) -----------------------------------CACGAAGCTGTAGCCGAGCTATGAGGGCTGTAGGCGTGGTGGAGGCCACATGTGAAGCCATTTTTGGGCTGGTGATGAGCATGGATGTAACTAGATACG
      APOSTART 7   (623) -----------------------------------CACGAAGCTGTAGCAGAGCTATGAGGGCTGTCGGCGTAGTGGAAGCCACATGTGAAGCCATTTTTGGGCTGGTGATGAGCATGGACGTGACTAGATACG
      APOSTART_8   (623) -----------------------------------CACGAAGCTGTAGCCGAGCTATGAGGGCTGTCGGCGTAGTGGAAGCCACATGCGAAGCCATTTTTGGGCTGGTGATGAGCATGGACATGACGAGATACG
      APOSTART_9   (620) -----------------------------------CACGAAGCTGTAGCCGAGCTATGAGGGCTGTCGGCGTAGTGGAAGCCACATGCGAAGCCATTTTTGGGCTGGTGATGAGCATGGACATGACGAGATACG
     APOSTART_10   (605) -----------------------------------CACGAAGCTGTAGCCGAGCTATGAGGGCTGTCGGCGTAGTGGAAGCCACATGCGAAGCCATTTTTGGGCTGGTGATGAGCATGGACATGACGAGATACG
     APOSTART_11   (617) -----------------------------------CACGAAGCTGTAGCCGAGCTATGAGGGCTGTCGGCGTAGTGGAAGCCACATGCGAAGCCATTTTTGGGCTGGTGATGAGCATGGACATGACGAGATACG
     APOSTART_12   (623) -----------------------------------CACGAAGCTGTAGCCGAGCTATGAGGGCTGTCGGCGTAGTGGAAGCCACATGCGAAGCCATTTTTGGGCTGGTGATGAGCATGGACGTGACGAGATACG
     APOSTART_14   (620) -----------------------------------CACGAAGCTGTAGCCGAGCTATGAGGGCTGTCGGCGTAGTGGAAGCCACATGCGAAGCCATTTTTGGGCTGGTGATGAGCATGGACGTGACGAGATACG
     APOSTART_15   (689) -----------------------------------CACGAAGCTGTAGCCGAGCTATGAGGGCTGTCGGCGTAGTGGAAGCCACATGCGAAGCCATTTTTGGGCTGGTGATGAGCATGGACGTGACGAGATACG
     APOSTART_13   (620) -----------------------------------CACGAAGCTGTAGCCGAGCTATGAGGGCTGTCGGCGTAGTGGAAGCCACATGCGAAGCCATTTTTGGGCTGGTGATGAGCATGGACGTGACGAGATACG
 APOSTART_1_GEN   (2221) TGACTATGAATCCTACTTTTCATGCCCTGTGTAGGCACGAAGCTGTAGCCGAGCTATGAGGGCTGTCGGCGTAGTGGAAGCCACATGCGAAGCCATTTTTGGGCTGGTGATGAGCATGGACATGACGAGATACG
 APOSTART_2_GEN   (2198) TGACTACCAATCGTACTTTTCATGCCCTGTGTAGGCACGAAACTGTAGCCGAGCTATGAGGGCTGTCGGCGTAATGGAAGCCACATGCGAAGCCATTTTTGGGCTGGTGATGAGCATGGACGTGACGAGATACG
 APOSTART_5_GEN   (2092) TGACTACGAATCGTACTTTTCTTGCCCCGTGTAGGCACGAAGCTGTAGCCGAGCTATGAGGGCTGTCGGCGTGGTGGAGGCCACATGTGAAGCCATTTTTGGGCTGGTGATGAGCATGGATGTGACTAGATACC
 APOSTART_6_GEN   (2083) TGACTACGAATCGTACTTTTCCTGCCCTGTGTAGGCACGAAGCTGTAGCCGAGCTATGAGGGCTGTAGGCGTGGTGGAGGCCACATGTGAAGCCATTTTTGGGCTGGTGATGAGCATGGATGTAACTAGATACG
 APOSTART_7_GEN   (2235) TGACTACGAATCGTACTTTTCATGCCTTGTGTAGGCACGAAGCTGTAGCAGAGCTATGAGGGCTGTCGGCGTAGTGGAAGCCACATGTGAAGCCATTTTTGGGCTGGTGATGAGCATGGACGTGACTAGATACG
 APOSTART_8_GEN   (2244) TGACTACGAATCGTACTTTTCATGCCTTGTGTAGGCACGAAGCTGTAGCCGAGCTATGAGGGCTGTCGGCGTAGTGGAAGCCACATGCGAAGCCATTTTTGGGCTGGTGATGAGCATGGACATGACGAGATACG
APOSTART_10_GEN   (2219) TGACTATGAATCCTACTTTTCATGCCCTGTGTAGGCACGAAGCTGTAGCCGAGCTATGAGGGCTGTCGGCGTAGTGGAAGCCACATGCGAAGCCATTTTTGGGCTGGTGATGAGCATGGACATGACGAGATACG
APOSTART_12_GEN   (2229) TGACTACGAATCGTACTTTTCATGCCCTGTGTAGGCACGAAGCTGTAGCCGAGCTATGAGGGCTGTCGGCGTAGTGGAAGCCACATGCGAAGCCATTTTTGGGCTGGTGATGAGCATGGACGTGACGAGATACG
```

Section 19

```
                   (2413) 2413      2420          2430          2440          2450          2460          2470          2480          2490          2500          2510          2520          2530        2546
APOSTART 1          (719) AGT-------------------------------------------------------------------------------------------------------GGGACTGTAGCTTTCGCTATGGCAGTTT
APOSTART 2          (719) AGT-------------------------------------------------------------------------------------------------------GGGACTGTAGCTTTCGCTATGGCAGTTT
APOSTART_3          (719) AGT-------------------------------------------------------------------------------------------------------GGGACTGTAGCTTTCGCTATGGCAGTTT
APOSTART_4          (722) AAT-------------------------------------------------------------------------------------------------------GGGACTGTAGCTTTCGCTATGGCAGTTT
APOSTART_5          (719) AGT-------------------------------------------------------------------------------------------------------GGGACTGTAGCTTTCGCTATGGCAGTTT
APOSTART_6          (719) AGT-------------------------------------------------------------------------------------------------------GGGACTGTAGCTTTCGCTATGGCAGTTT
APOSTART 7          (722) AGT-------------------------------------------------------------------------------------------------------GGGACTGTAGCTTTCGCTATGGAAGTTT
APOSTART_8          (722) AGT-------------------------------------------------------------------------------------------------------GGGACTGTAGCTTTCGCTATGGCAGTTT
APOSTART_9          (719) AGT-------------------------------------------------------------------------------------------------------GGGACTGTAGCTTTCGCTATGGCAGTTT
APOSTART_10         (704) AGT-------------------------------------------------------------------------------------------------------GGGACTGTAGCTTTCGCTATGGCAGTTT
APOSTART_11         (716) AGT-------------------------------------------------------------------------------------------------------GGGACTGTAGCTTTCGCTATGGCAGTTT
APOSTART_12         (722) AAT-------------------------------------------------------------------------------------------------------GGGACTGTAGCTTTCGCTATGGCAGTTT
APOSTART_14         (719) AAT-------------------------------------------------------------------------------------------------------GGGACTGTAGCTTTCGCTATGGCAGTTT
APOSTART_15         (788) AAT-------------------------------------------------------------------------------------------------------GGGACTGTAGCTTTCGCTATGGCAGTTT
APOSTART_13         (719) AAT-------------------------------------------------------------------------------------------------------GGGACTGTAGCTTTCGCTATGGCAGTTT
APOSTART_1_GEN     (2355) AGTAAGCGT----GCTCTTTAACTCTCTGCTGTT----------------------------ACAACTACCGATGCTGATGTCGATATTTATTCTCCAACATAGGTGGGACTGTAGCTTTCGCTATGGCAGTTT
APOSTART_2_GEN     (2332) AGTAAGCGTCTGTCCTCTTTAACTCTCTGCTGTT----------------------------ACAACTACCGATGCTGATGTCGATATTTATTCTCCAACATAGGTGGGACTGTAGCTTTCGCTATGGCAGTTT
APOSTART_5_GEN     (2226) AGTAAGCGTCCGGCCTCTTTA-CTCTCCGATGTTCCAACT-----------------------------CCGATGCTGATGCCGATATTTT---CCAATCATAGGTGGGACTGTAGCTTTCGCTATGGCAGTTT
APOSTART_6_GEN     (2217) AGTAAGCGTCTGGCCTCTTTAACTCTCCGATGTTC------C----------------------AACTACCAATGCTGATGCCAACATTT--TCACAATCATAGGTGGGACTGTAGCTTTCGCTATGGCAGTTT
APOSTART_7_GEN     (2369) AGTAAGTGTCTGGCCTCTTTAACTCTCTGCTGTTCCAACTACCGATGCTGCCCTTTAAAAAAAAAACTACCGATGCTGATGTCCATCTTTATTCTCCACTATAGGTGGGACTGTAGCTTTCGCTATGGAAGTTT
APOSTART_8_GEN     (2378) AGTAAGTGTCTGGCCTCTTTAACTCTCTGCTGTTCCAACTACCGATGCTGCCCTTTAAAAAAAAAACTACCGATGCTGATGTCCATCTTTATTCTCCACTATAGGTGGGACTGTAGCTTTCGCTATGGCAGTTT
APOSTART_10_GEN    (2353) AGTAAGCGT--G-----------------------------C-TCTTTAACTCTCTGCTGTTACAACTACCGATGCTGATGTCGATATTTATTCTCCAACATAGGTGGGACTGTAGCTTTCGCTATGGCAGTTT
APOSTART_12_GEN    (2363) AATAAGCCTCTGTCCTCTTTAACTATCTG------------CTGTT----------------ACAACTACCGATGCTGATGTCGATATTTATTCTCCAACATAGGTGGGACTGTAGCTTTCGCTATGGCAGTTT
```

Section 20

```
                   (2547) 2547         2560          2570          2580          2590          2600          2610          2620          2630          2640          2650          2660          2670        2680
APOSTART 1          (750) AGTCGAAGAGGTTGATGGCCACACCGCCATACTCTATCATAAGTTGCAGCTGCACTGGTGTCCAATGT------------------------------------------------------------------
APOSTART 2          (750) AGTCGAAGAGGTTGATGGCCACACCGCCATACTCTATCATAAGTTGCAGCTGCACTGGTGTCCAATGT------------------------------------------------------------------
APOSTART_3          (750) AGTCGAAGAGGTTGATGGCCACACCGCCATACTCTATCATAAGTTGCAGCTGCACTGGTGTCCAATGT------------------------------------------------------------------
APOSTART_4          (753) AGTCGAAGAGGTTGATGGCCACACCGTGATACTCTATCATAAGTTGCAGCTGCACTGGTGTCCAATGT------------------------------------------------------------------
APOSTART_5          (750) GGTCGAAGAAGTCGATGGCCACACCGCGATACTCTATCACAAGTTGCAGCTGCACTGGTGTCCAATGT------------------------------------------------------------------
APOSTART_6          (750) GGTCGAAGAAGTCGATGGCCACACCGCGATACTCTATCACAAGTTGCAGCTGCACTGGTGTCCAATGT------------------------------------------------------------------
APOSTART 7          (753) AGTCGAAGAGGTTGATGGGCACACCGCCATACTCTATCATAAGTTGCAGCTGCACTGGTGTCCAATGT------------------------------------------------------------------
APOSTART_8          (753) AGTCGAAGAGGTTGATGGCCACACCGCCATACTCTATCATAAGTTGCAGCTGCACTGGTGTCCAATGT------------------------------------------------------------------
APOSTART_9          (750) AGTCGAAGAGGTTGATGGCCACACCGCCATACTCTATCATAAGTTGCAGCTGCACTGGTGTCCAATGT------------------------------------------------------------------
APOSTART_10         (735) AGTCGAAGAGGTTGATGGCCACACCGCCATACTCTATCATAAGTTGCAGCTGCACTGGTGTCCAATGT------------------------------------------------------------------
APOSTART_11         (747) AGTCGAAGAGGTTGATGGCCACACCGCCATACTCTATCATAAGTTGCAGCTGCACTGGTGTCCAATGT------------------------------------------------------------------
APOSTART_12         (753) AGTCGAAGAGGTTGATGGCCACACCGCGATACTCTATCATAAGTTGCAGCTGCACTGGTGTCCAATGT------------------------------------------------------------------
APOSTART_14         (750) AGTCGAAGAGGTTGATGGCCACACCGTGATACTCTATCATAAGTTGCAGCTGCACTGGTGTCCAATGT------------------------------------------------------------------
APOSTART_15         (819) AGTCGAAGAGGTTGATGGCCACACCGTGATACTCTATCATAAGTTGCAGCTGCACTGGTGTCCAATGT------------------------------------------------------------------
APOSTART_13         (750) AGTCGAAGAGGTTGATGGCCACACCGCGATACTCTATCATAAGTTGCAGCTGCACTGGTGTCCAATGT------------------------------------------------------------------
APOSTART_1_GEN     (2457) AGTCGAAGAGGTTGATGGCCACACCGCCATACTCTATCATAAGTTGCAGCTGCACTGGTGTCCAATGTAA-CCACATTT---CATGTCAACAACTGTGTACCGTCTGTAAT---TTTTTGGATTGTCTGTCAGA
APOSTART_2_GEN     (2438) AGTCGAAGAGGTTGATGGCCACACCGCCATACTCTATCATAAGTTGCAGCTGCACTGGTGTCCAATGTAA-CCACATTT---CATGTCAACAACTGTGTACCGTCTGTAAT---TTTTTGGATTGTCTGTCAGA
APOSTART_5_GEN     (2327) GGTCGAAGAAGTCGATGGCCACACCGCGATACTCTATCACAAGTTGCAGCTGCACTGGTGTCCAATGTAAACCATATTCTCATATTTCAACAACCGTTTAGCGTCTGTAATACGTTTTTGGATTGTCTGTCAGA
APOSTART_6_GEN     (2321) GGTCGAAGAAGTCGATGGCCACACCGCGATACTCTATCACAAGTTGCAGCTGCACTGGTGTCCAATGTAAACCATATTCTCATATTTCAACAACCGTTTAGCGTCTGTAATACGTTTTTGGATTGTCTGTCAGA
APOSTART_7_GEN     (2503) AGTCGAAGAGGTTGATGGGCACACCGCCATACTCTATCATAAGTTGCAGCTGCACTGGTGTCCAATGTAA-CCACATTTTCACATGTCAACAACTGTGTACCGTCTATAAA---TTTTTGGATTGTCTGTCAGA
APOSTART_8_GEN     (2512) AGTCGAAGAGGTTGATGGCCACACCGCCATACTCTATCATAAGTTGCAGCTGCACTGGTGTCCAATGTAA-CCACATTTTCACATGTCAACAACTGTGTACCGTCTATAAA---TTTTTGGATTGTCTGTCAGA
APOSTART_10_GEN    (2455) AGTCGAAGAGGTTGATGGCCACACCGCCATACTCTATCATAAGTTGCAGCTGCACTGGTGTCCAATGTAA-CCACATTT---CATGTCAACAACTGTGTACCGTCTGTAAT---TTTTTGGATTGTCTGTCAGA
APOSTART_12_GEN    (2469) AGTCGAAGAGGTTGATGGCCACACCGCGATACTCTATCATAAGTTGCAGCTGCACTGGTGTCCAATGTAA-CCACATTT---CATGTCAACAACTGTGTACCGTCTGTAAT---TTTTTGGATTGTCTGTCAGA
```

Section 21

```
        (2681) 2681     2690      2700      2710      2720      2730      2740      2750      2760      2770      2780      2790      2800      2814
APOSTART 1   (818) ----------------------------------------TAGTGTGGCCTAAAGATCTGTGTTACGTTCGTTACTGGCGACGCAATGATGATGGAAGCTATG-------------------------------
APOSTART 2   (818) ----------------------------------------TAGTGTGGCCTAAAGATCTGTGTTACGTTCGTTACTGGCGACGCAATGATGATGGAAGCTATG-------------------------------
APOSTART_3   (818) ----------------------------------------TAGTGTGGCCTAAAGATCTGTGTTACGTTCGTTACTGGCGACGCAATGATGATGGAAGCTATG-------------------------------
APOSTART_4   (821) ----------------------------------------TAGTGTGGCCTAGAGATCTCTGTTATGTTCGTTACTGGCGACGCAATGATGATGGAAGCTATG-------------------------------
APOSTART_5   (818) ----------------------------------------TAGTCTGGCCTAGGGATCTCTGTTACGTTCGTTACTGGCGGCGCAATGATGATGGAAGCTATG-------------------------------
APOSTART_6   (818) ----------------------------------------TAGTCTGGCCTAGGGATCTCTGTTACGTTCGTTACTGGCGGCGCAATGATGATGGAAGCTATG-------------------------------
APOSTART 7   (821) ----------------------------------------TAGTGTGGCCTAGAGATCTCTGTTACGTTCGTTACTGGCGACGCAATGATGATGGAAGCTATG-------------------------------
APOSTART_8   (821) ----------------------------------------TAGTGTGGCCTAGAGATCTGTGTTACGTTCGTTACTGGCGACGCAATGATGATGGAAGCTATG-------------------------------
APOSTART_9   (818) ----------------------------------------TAGTGTGGCCTAGAGATCTGTGTTACGTTCGTTACTGGCGACGCAATGATGATGGAAGCTATG-------------------------------
APOSTART_10  (803) ----------------------------------------TAGTGTGGCCTAGAGATCTGTGTTACGTTCGTTACTGGCGACGCAATGATGATGGAAGCTATG-------------------------------
APOSTART_11  (815) ----------------------------------------TAGTGTGGCCTAGAGATCTGTGTTACGTTCGTTACTGGCGACGCAATGATGATGGAAGCTATG-------------------------------
APOSTART_12  (821) ----------------------------------------TAGTGTGGCCTAGAGATCTCTGTTATGTTCGTTACTGGCGACGCAATGATGATGGAAGCTATG-------------------------------
APOSTART_14  (818) ----------------------------------------TAGTGTGGCCTAGAGATCTCTGTTATGTTCGTTACTGGCGACGCAATGATGATGGAAGCTATG-------------------------------
APOSTART_15  (887) ----------------------------------------TAGTGTGGCCTAGAGATCTCTGTTATGTTCGTTACTGGCGACGCAATGATGATGGAAGCTATG-------------------------------
APOSTART_13  (818) ----------------------------------------TAGTGTGGCCTAGAGATCTCTGTTATGTTCGTTACTGGCGACGCAATGATGATGGAAGCTATG-------------------------------
APOSTART_1_GEN (2584) CTGAAATTTACATTCTTCCATCCTACATGAATATTCAGGTTAGTGTGGCCTAAAGATCTGTGTTACGTTCGTTACTGGCGACGCAATGATGATGGAAGCTATGGTAAACCCTTGAATTTTTCATGTTCCTGTCC
APOSTART_2_GEN (2565) CTGAAATTTACATTCTTCCATCCTACATGAATATTCAGGTTAGTGTGGCCTAAAGATCTGTGTTACGTTCGTTACTGGCGACGCAATGATGATGGAAGCTATGGTAAACCCTTGAATTTTTCATGTTCCTGTCC
APOSTART_5_GEN (2461) TTGAACCTTACATTCTTCCATCTTGCAT-AACATACAGGTTAGTCTGGCCTAGGGATCTCTGTTACGTTCGTTACTGGCGGCGCAATGATGATGGAAGCTATGGTAAATTCTTGAATTTTCCATGCTCATTTTC
APOSTART_6_GEN (2455) TTGAACCTTACATTCTTCCATCTTGCAT-AACATACAGGTTAGTCTGGCCTAGGGATCTCTGTTACGTTCGTTACTGGCGGCGCAATGATGATGGAAGCTATGGTAAATTATTGAATTTTCCATGCTCATTTTC
APOSTART_7_GEN (2633) CTGAAATTTACATTCTTCCATCCTACATGAATATTCAGGTTAGTGTGGCCTAGAGATCTCTGTTACGTTCGTTACTGGCGACGCAATGATGATGGAAGCTATGGTAAACCCTTGAATTTTCCATGTTCCTGTAC
APOSTART_8_GEN (2642) CTGAAATTTACATTCTTCCATCCTACATGAATATTCAGGTTAGTGTGGCCTAGAGATCTGTGTTACGTTCGTTACTGGCGACGCAATGATGATGGAAGCTATGGTAAACCCTTGAATTTTCCATGTTCCTGTAC
APOSTART_10_GEN (2582) CTGAAATTTACATTCTTCCATCCTACATGAATATTCAGGTTAGTGTGGCCTAGAGATCTGTGTTACGTTCGTTACTGGCGACGCAATGATGATGGAAGCTATGGTAAACCCTTGAATTTTTCATGTTCCTGTCC
APOSTART_12_GEN (2596) CTGAAATTTACATTCTTCCATCCTACATGAATATTCAGGTTAGTGTGGCCTAGAGATCTCTGTTATGTTCGTTACTGGCGACGCAATGATGATGGAAGCTATGGTAAACTCTTGAATTTTTCATGTTCCTGTCC
```

Section 22

```
        (2815) 2815 2820      2830      2840      2850      2860      2870      2880      2890      2900      2910      2920      2930      2948
APOSTART 1   (881) ---------------------------------------------------------------TTGTGCTGTTTCGATCTATCGAACATCCAAACTGTGGCCGCCAACGAGGATACGTAAGGGCTTTTATTGAA
APOSTART 2   (881) ---------------------------------------------------------------TTGTGCTGTTTCGATCTATCGAACATCCAAACTGTGGCCGCCAACGAGGATACGTAAGGGCTTTTATTGAA
APOSTART_3   (881) ---------------------------------------------------------------TTGTGCTGTTTCGATCTATCGAACATCCAAACTGTGGCCGCCAACGAGGATACGTAAGGGCTTTTATTGAA
APOSTART_4   (884) ---------------------------------------------------------------TTGTGCTCTTTCGATCTATCGAACATCCAAACTGTGGCCGCCAACGAGGATATGTAAGGGCTTTTATTGAA
APOSTART_5   (881) ---------------------------------------------------------------TTGTGCTGTTTCGATCTATCGAGCATCCAAACTGTGGCCACCAACGGGGATATGTAAGGGCTTTTATTGAA
APOSTART_6   (881) ---------------------------------------------------------------TTGTGCTGTTTCGATCTATCGAGCATCCAAACTGTTGCCGCCAACGGGGATATGTAAGGGCTTTTATTGAA
APOSTART 7   (884) ---------------------------------------------------------------TTGTGCTGTTTCGATCTATCGAACATCCAAACTGTGGCCGCCAACGAGGATACGTAAGGGCTTTTATTGAA
APOSTART_8   (884) ---------------------------------------------------------------TTGTGCTGTTTCGATCTATCGAACATCCAAACTGTGGCCGCCAACGAGGATACGTAAGGGCTTTTATTGAA
APOSTART_9   (881) ---------------------------------------------------------------TTGTGCTGTTTCGATCTATCGAACATCCAAACTGTGGCCGCCAACGAGGATACGTAAGGGCTTTTATTGAA
APOSTART_10  (866) ---------------------------------------------------------------TTGTGCTGTTTCGATCTATCGAACATCCAAACTGTGGCCGCCAACGAGGATACGTAAGGGCTTTTATTGAA
APOSTART_11  (878) ---------------------------------------------------------------TTGTGCTGTTTCGATCTATCGAACATCCAAACTGTGGCCGCCAACGAGGATACGTAAGGGCTTTTATTGAA
APOSTART_12  (884) ---------------------------------------------------------------TTGTGCTCTTTCGATCTATCGAACATCCAAACTGTGGCCGCCAACGAGGATATGTAAGGGCTTTTATTGAA
APOSTART_14  (881) ---------------------------------------------------------------TTGTGCTCTTTCGATCTATCGAACATCCAAACTGTGGCCGCCAACGAGGATATGTAAGGGCTTTTATTGAA
APOSTART_15  (950) ---------------------------------------------------------------TTGTGCTCTTTCGATCTATCGAACATCCAAACTGTGGCCGCCAACGAGGATATGTAAGGGCTTTTATTGAA
APOSTART_13  (881) ---------------------------------------------------------------TTGTGCTCTTTCGATCTATCGAACATCCAAACTGTGGCCGCCAACGAGGATATGTAAGGGCTTTTATTGAA
APOSTART_1_GEN (2718) CGTCACCTTCGCTGTCTGTATCATCTTTTGATCGACAATGTATATTTGTTCTTACACATACAGTTGTGCTGTTTCGATCTATCGAACATCCAAACTGTGGCCGCCAACGAGGATACGTAAGGGCTTTTATTGAA
APOSTART_2_GEN (2699) CGTCACCTTCGCTGTCTGTACCATCTTTTGATCGACAATGTATATTTGTTCTTACACATACAGTTGTGCTGTTTCGATCTATCGAACATCCAAACTGTGGCCGCCAACGAGGATACGTAAGGGCTTTTATTGAA
APOSTART_5_GEN (2594) ---AACTTTTTCTGTTTGTACCATCTTCTGATTGGCATTGTATGTTT-TTCTTACACATATAGTTGTGCTGTTTCGATCTATCGAGCATCCAAACTGTGGCCACCAACGGGGATATGTAAGGGCTTTTATTGAA
APOSTART_6_GEN (2588) ---AACTTTTTCTGTTTGTACCATCTTCTGATTGGCATTATATGTTTAT-CTTACACATATAGTTGTGCTGTTTCGATCTATCGAGCATCCAAACTGTTGCCGCCAACGGGGATATGTAAGGGCTTTTATTGAA
APOSTART_7_GEN (2767) CGTCACCTTCGCTGTCTGTACCATCTTTTAATCGACAATGTATATTTGTTCTTACACATACAGTTGTGCTGTTTCGATCTATCGAACATCCAAACTGTGGCCGCCAACGAGGATACGTAAGGGCTTTTATTGAA
APOSTART_8_GEN (2776) CGTCACCTTCGCTGTCTGTACCATCTTTTAATCGACAATGTATATTTGTTCTTACACATACAGTTGTGCTGTTTCGATCTATCGAACATCCAAACTGTGGCCGCCAACGAGGATACGTAAGGGCTTTTATTGAA
APOSTART_10_GEN (2716) CGTCACCTTCGCTGTCTGTACCATCTTTTGATCGACAATGTATATTTGTTCTTACACATACAGTTGTGCTGTTTCGATCTATCGAACATCCAAACTGTGGCCGCCAACGAGGATACGTAAGGGCTTTTATTGAA
APOSTART_12_GEN (2730) CGTCACCTTCGCTGTCTGTATCATCTTTTGATCGACAATGTATATTTGTTCTTACACATACAGTTGTGCTCTTTCGATCTATCGAACATCCAAACTGTGGCCGCCAACGAGGATATGTAAGGGCTTTTATTGAA
```

```
                                                                                                                                                      Section 23
                     (2949) 2949       2960      2970      2980      2990      3000      3010      3020      3030      3040      3050      3060      3070        3082
        APOSTART 1    (952) AGT----------------------------------------------------------------------------------------------------------------------GGAGGGTTCAAGA
        APOSTART 2    (952) AGT----------------------------------------------------------------------------------------------------------------------GGAGGGTTCAAGA
        APOSTART_3    (952) AGT----------------------------------------------------------------------------------------------------------------------GGAGGGTTCAAGA
        APOSTART_4    (955) AGT----------------------------------------------------------------------------------------------------------------------GGAGGGTTCAAGA
        APOSTART_5    (952) AGG----------------------------------------------------------------------------------------------------------------------GGAGGTTTCAAGA
        APOSTART_6    (952) AGT----------------------------------------------------------------------------------------------------------------------GGAGGTTTCAAGA
        APOSTART 7    (955) AGT----------------------------------------------------------------------------------------------------------------------GGAGGGTTCAAGA
        APOSTART_8    (955) AGT----------------------------------------------------------------------------------------------------------------------GGAGGGTTCAAGA
        APOSTART_9    (952) AGT----------------------------------------------------------------------------------------------------------------------GGAGGGTTCAAGA
        APOSTART_10   (937) AGT----------------------------------------------------------------------------------------------------------------------GGAGGGTTCAAGA
        APOSTART_11   (949) AGT----------------------------------------------------------------------------------------------------------------------GGAGGGTTCAAGA
        APOSTART_12   (955) AGT----------------------------------------------------------------------------------------------------------------------GGAGGGTTCAAGA
        APOSTART_14   (952) AGT----------------------------------------------------------------------------------------------------------------------GGAGGGTTCAAGA
        APOSTART_15  (1021) AGT----------------------------------------------------------------------------------------------------------------------GGAGGGTTCAAGA
        APOSTART_13   (952) AGT----------------------------------------------------------------------------------------------------------------------GGAGGGTTCAAGA
    APOSTART_1_GEN (2852) AGTAAGTCTTCTTCTGACCATCTGATATTCTTCATATTTACCCGGGAGGATGAGACACTTAAAATAAACAAAATTAACTTGCGTTACTTT-CTTTCTCTGAAC-TTCCTACTAGTTCAGGTGGAGGGTTCAAGA
    APOSTART_2_GEN (2833) AGTAAGTCTTCTTCTGACCATCTGATATTCGTCATATTTACCGGGGAGGATGAGACACTTAAAATAAACAAAATTAACTTGAGCTACTTT-CTTTCTCTGAAC-TTCCTACTAGTTCAGGTGGAGGGTTCAAGA
    APOSTART_5_GEN (2724) AGTAAGTCTCCTTCTCACCATTTGTGACCGTTCAT-TTTACCCGAGAGGATGATACATTTCAAATAAACAAAATTAACTTGGATTCGTTTTCCTTTTTTGAAAATTCCTACTAGTTCAGG-GGAGGTTTCAAGA
    APOSTART_6_GEN (2718) AGTAAGTCTCCTTCTCACCATTTGTGACCCTTCAT-TTTACCCGAGTGGATGATACATTTCAAATAAACAAAATTAACTTGGATTCGTTTTCCTTTTCTGAAA-TTCCTACTAGTTCAGGTGGAGGTTTCAAGA
    APOSTART_7_GEN (2901) AGTAAGTCTTCTTCTGACCATCTGATATTCTTCATATTTACCCGAGGGGATGAGACACTTAAAATAAACAAAATTAACTTGAGTTACTTT-CTTTCTCTGAAC-TTCCTACTAGTTCAGGTGGAGGGTTCAAGA
    APOSTART_8_GEN (2910) AGTAAGTCTTCTTCTGACCATCTGATATTCTTCATATTTACCCGAGGGGATGAGACACTTAAAATAAACAAAATTAACTTGAGTTACTTT-CTTTCTCTGAAC-TTCCTACTAGTTCAGGTGGAGGGTTCAAGA
   APOSTART_10_GEN (2850) AGTAAGTCTTCTTCTGACCATCTGATATTCTTCATATTTACCCGGGAGGATGAGACACTTAAAATAAACAAAATTAACTTGCGTTACTTT-CTTTCTCTGAAC-TTCCTACTAGTTCAGGTGGAGGGTTCAAGA
   APOSTART_12_GEN (2864) AGTAAGTCTTCTTCTGACCATCTGATATTCTTCATATTTACCCGGGAGGATGAGACACTTAAAATAAACAAAATTAACTTGAGCTACTTT-CTTTCTCTGAAC-TTCCTACTAGTTCAGGTGGAGGGTTCAAGA

                                                                                                                                                      Section 24
                     (3083) 3083    3090      3100      3110      3120      3130      3140      3150      3160      3170      3180      3190      3200          3216
        APOSTART 1    (968) TTTCTCCTCTCAAATGTCGCAACGGACGGCCACGTACTCAAGTTCAACACCTTATGCAGATTGACCTGAGAGGATGGTTCCTGAACTACTCCCCTTCCTTTCAGTATCACTCTTTGCTGCAGATACAGAACTGT
        APOSTART 2    (968) TTTCTCCTCTCAAATGTCGCAACGGACGACCACGTACTCAAGTTCAACACCTTATGCAGATTGACCTGAGAGGATGGTTCCTGAACTACTCCCCTTCCTTTCGGTATCACTCTTTGCTGCAGATACAGAACTGT
        APOSTART_3    (968) TTTCTCCTCTCAAATGTCGCAACGGACGGCCACGTACTCAAGTTCAACACCTTATGCAGATTGACCTGAGAGGATGGTTCCTGAACTACTCCCCTTCCTTTCAGTATCACTCTTTGCTGCAGATACAGAACTGT
        APOSTART_4    (971) TTTCTCCTCTCAAATGTCGCAACGGACGACCACATACTCAAGTTCAACACCTTATGCAGATTGACCTGAGAGGATGGTTCCTGAACTACTCCCCTTCCTTTCAGTATCACTCTTTGCTGCAGATACAGAACTGT
        APOSTART_5    (968) TTTCTCCTCTCAAGTGTCGCAACGGCAGGCCGCGTACTCAAGTTCAACACCTTATGCAGATTGATCTGAGAGGATGGTTTCTGAACTACTCTCCTTCCTTTCAGTATCATTCTTTGCTGCAGATACAAAACTGT
        APOSTART_6    (968) TTTCTCCTCTCAAGTGTCGCAACGGACGGCCGCGTACTCAAGTTCAACACCTTATGCAGATTGATCTGAGAGGATGGTTTCTGAACTACTCTCCTTCCTTTCAGTATCATTCTTTGCTGCAGATACAAAACTGT
        APOSTART 7    (971) TTTCTCCTCTCAAATGTCGCAACGGACGACCACGTACTCAAGTTCAACACCTTATGCAGATTGACCTGAGAGGATGGTTCCTGAACTACTCCCCTTCCTTTCAGTATCACTCTTTGCTGCAGATACAGAACTGT
        APOSTART_8    (971) TTTCTCCTCTCAAATGTCGCAACGGACGGCCACGTACTCAAGTTCAACACCTTATGCAGATTGACCTGAGAGGATGGTTCCTGAACTACTCCCCTTCCTTTCAGTATCACTCTTTGCTGCAGATACAGAACTGT
        APOSTART_9    (968) TTTCTCCTCTCAAATGTCGCAACGGACGGCCACGTACTCAAGTTCAACACCTTATGCAGATTGACCTGAGAGGATGGTTCCTGAACTACTCCCCTTCCTTTCAGTATCACTCTTTGCTGCAGATACAGAACTGT
        APOSTART_10   (953) TTTCTCCTCTCAAATGTCGCAACGGACGGCCACGTACTCAAGTTCAACACCTTATGCAGATTGACCTGAGAGGATGGTTCCTGAACTACTCCCCTTCCTTTCAGTATCACTCTTTGCTGCAGATACAGAACTGT
        APOSTART_11   (965) TTTCTCCTCTCAAATGTCGCAACGGACGGCCACGTACTCAAGTTCAACACCTTATGCAGATTGACCTGAGAGGATGGTTCCTGAACTACTCCCCTTCCTTTCAGTATCACTCTTTGCTGCAGATACAGAACTGT
        APOSTART_12   (971) TTTCTCCTCTCAAATGTCGCAACGGACGACCACGTACTCAAGTTCAACACCTTATGCAGATTGACCTGAGAGGATGGTTCCTGAACTACTCCCCTTCCTTTCAGTATCACTCTTTGCTGCAGATACAGAACTGT
        APOSTART_14   (968) TTTCTCCTCTCAAATGTCGCAACGGACGACCACATACTCAAGTTCAACACCTTATGCAGATTGACCTGAGAGGATGGTTCCTGAACTACTCCCCTTCCTTTCAGTATCACTCTTTGCTGCAGATACAGAACTGT
        APOSTART_15  (1037) TTTCTCCTCTCAAATGTCGCAACGGACGACCACATACTCAAGTTCAACACCTTATGCAGATTGACCTGAGAGGATGGTTCCTGAACTACTCCCCTTCCTTTCAGTATCACTCTTTGCTGCAGATACAGAACTGT
        APOSTART_13   (968) TTTCTCCTCTCAAATGTCGCAACGGACGACCACGTACTCAAGTTCAACACCTTATGCAGATTGACCTGAGAGGATGGTTCCTGAACTACTCCCCTTCCTTTCAGTATCACTCTTTGCTGCAGATACAGAACTGT
    APOSTART_1_GEN (2984) TTTCTCCTCTCAAATGTCGCAACGGACGGCCACGTACTCAAGTTCAACACCTTATGCAGATTGACCTGAGAGGATGGTTCCTGAACTACTCCCCTTCCTTTCAGTATCACTCTTTGCTGCAGATACAGAACTGT
    APOSTART_2_GEN (2965) TTTCTCCTCTCAAATGTCGCAACGGACGACCACGTACTCAAGTTCAACACCTTATGCAGATTGACCTGAGAGGATGGTTCCTGAACTACTCCCCTTCCTTTCGGTATCACTCTTTGCTGCAGATACAGAACTGT
    APOSTART_5_GEN (2856) TTTCTCCTCTCAAGTGTCGCAACGGCAGGCCGCGTACTCAAGTTCAACACCTTATGCAGATTGATCTGAGAGGATGGTTTCTGAACTACTCTCCTTCCTTTCAGTATCATTCTTTGCTGCAGATACAAAACTGT
    APOSTART_6_GEN (2850) TTTCTCCTCTCAAGTGTCGCAACGGACGGCCGCGTACTCAAGTTCAACACCTTATGCAGATTGATCTGAGAGGATGGTTTCTGAACTACTCTCCTTCCTTTCAGTATCATTCTTTGCTGCAGATACAAAACTGT
    APOSTART_7_GEN (3033) TTTCTCCTCTCAAATGTCGCAACGGACGACCACGTACTCAAGTTCAACACCTTATGCAGATTGACCTGAGAGGATGGTTCCTGAACTACTCCCCTTCCTTTCAGTATCACTCTTTGCTGCAGATACAGAACTGT
    APOSTART_8_GEN (3042) TTTCTCCTCTCAAATGTCGCAACGGACGGCCACGTACTCAAGTTCAACACCTTATGCAGATTGACCTGAGAGGATGGTTCCTGAACTACTCCCCTTCCTTTCAGTATCACTCTTTGCTGCAGATACAGAACTGT
   APOSTART_10_GEN (2982) TTTCTCCTCTCAAATGTCGCAACGGACGGCCACGTACTCAAGTTCAACACCTTATGCAGATTGACCTGAGAGGATGGTTCCTGAACTACTCCCCTTCCTTTCAGTATCACTCTTTGCTGCAGATACAGAACTGT
   APOSTART_12_GEN (2996) TTTCTCCTCTCAAATGTCGCAACGGACGACCACGTACTCAAGTTCAACACCTTATGCAGATTGACCTGAGAGGATGGTTCCTGAACTACTCCCCTTCCTTTCAGTATCACTCTTTGCTGCAGATACAGAACTGT
```

```
                                                                                                          Section 25
(3217)              3217         3230      3240      3250      3260      3270      3280      3290      3300      3310      3320      3330      3340      3350
APOSTART 1 (1102)   GTTGCTGG---------------------------------------------------------------------------------------------ACTGCGCGAATACTTTTCACAAACAGATGAATG
APOSTART 2 (1102)   GTTGCTGG---------------------------------------------------------------------------------------------ACTGCGCGAATACTTTTCACAAACAGATGAATG
APOSTART_3 (1102)   GTTGCTGG---------------------------------------------------------------------------------------------ACTGCGCGAATACTTTTCACAAACAGATGAATG
APOSTART_4 (1105)   GTTGCTGGG---------------------------------------------------------------------------------------------CTGCGCGAATACTTTTCACAAACAGATGAATG
APOSTART_5 (1102)   GTTGCTGGG---------------------------------------------------------------------------------------------CTGCGCGAATACTTTTCACAAACAGATGAATG
APOSTART_6 (1102)   GTTGCTGGG---------------------------------------------------------------------------------------------CTGCGCGAATACTTTTCACAAACAGATGAATG
APOSTART 7 (1105)   GTTGCTGG---------------------------------------------------------------------------------------------ACTGCGCGAATACTTTTCACAAACAGATGAATG
APOSTART_8 (1105)   GTTGCTG------------------------------------------------------------------------------------------------------------------------ATGAATG
APOSTART_9 (1102)   GTTGCTGG---------------------------------------------------------------------------------------------ACTGCGCGAATACTTTTCACAAACAGATGAATG
APOSTART_10 (1087)  GTTGCTGG---------------------------------------------------------------------------------------------ACTGCGCGAATACTTTTCACAAACAGATGAATG
APOSTART_11 (1099)  GTTGCTGG---------------------------------------------------------------------------------------------ACTGCGCGAATACTTTTCACAAACAGATGAATG
APOSTART_12 (1105)  GTTGCTGGG---------------------------------------------------------------------------------------------CTGCGCGAATACTTTTCACAAACAGATGAATG
APOSTART_14 (1102)  GTTGCTG------------------------------------------------------------------------------------------------------------------------ATGAATG
APOSTART_15 (1171)  GTTGCTGGG---------------------------------------------------------------------------------------------CTGCGCGAATACTTTTCACAAACAGATGAATG
APOSTART_13 (1102)  GTTGCTG------------------------------------------------------------------------------------------------------------------------ATGAATG
APOSTART_1_GEN (3118)  GTTGCTGGTACTGCATTTGGTTTTTCCTTATTAACAACC--TGAACACAGTTCACCAATTCATACTGGTACTTGTGGATTTAACTGACACTTACTGCCAGGACTGCGCGAATACTTTTCACAAACAGATGAATG
APOSTART_2_GEN (3099)  GTTGCTGGTACTGCATTTGGTTTTTCCTTATTAACAACCTCTGAACACAGTTCACCAATTCATATTGGTACTTGTGGATTTAACTGACACTTACTGCCAGGACTGCGCGAATACTTTTCACAAACAGATGAATG
APOSTART_5_GEN (2990)  GTTGCTGGTACTGCATGTG-TTTTCCCTTATTGGAAACC--TGAACATAATTCACCAATTCATTCTGGTACTTGTGGATTTAACTGACACTTACTGCCAGGGCTGCGCGAATACTTTTCACAAACAGATGAATG
APOSTART_6_GEN (2984)  GTTGCTGGTACTGCATGTGCTTTTCCCTTATTGGCAACC--TGAACATAATTCACCAATTCATTCTGGTACTTGTGGATTTAACTGACACTTACTGCCAGGGCTGCGCGAATACTTTTCACAAACAGATGAATG
APOSTART_7_GEN (3167)  GTTGCTGGTACTGCATTTGGTTTTCCCTTATTAACAACC--TGAACACAGTTCACCAATTCATACTGGTACTTGTGTTTTTAACTGACACTTACTGCCAGGACTGCGCGAATACTTTTCACAAACAGATGAATG
APOSTART_8_GEN (3176)  GTTGCTGGTACTGCATTTGGTTTTCCCTTATTAACAACC--TGAACACAGTTCACCAATTCATACTGGTACTTGTGTTTTTAACTGACACTTACTGCCAGGACTGCGCGAATACTTTTCACAAACAGATGAATG
APOSTART_10_GEN (3116) GTTGCTGGTACTGCATTTGGTTTTTCCTTATTAACAACC--TGAACACAGTTCACCAATTCATACTGGTACTTGTGGATTTAACTGACACTTACTGCCAGGACTGCGCGAATACTTTTCACAAACAGATGAATG
APOSTART_12_GEN (3130) GTTGCTGGTACTGCATTTGGTTTTTCCTTATTAACAACCACTGAACACAGTTCACCAATTCATATTGGTACTTGTGGATTTAACTGACACTTACTGCCAGGGCTGCGCGAATACTTTTCACAAACAGATGAATG
```

```
                                                                                                          Section 26
(3351)              3351     3360      3370      3380      3390      3400      3410      3420      3430      3440      3450      3460      3470         3484
APOSTART 1 (1143)   CCATATAACTCCAAGGATTCCTGTGATGGAAAACATGGTTGATCCATCGATGCCGAAAAGCCAGAAGCTACATGAAATGGAGTCCAAGACTAAACCAGCACATGGAGGACAAGCAGATAATAAAAGCATGTCGA
APOSTART 2 (1143)   CCATATAACTCCAAGGATTCCTGTGATGGAAAACATGGTTGATCCATCGATGCCGAAAAGCCAGAAGCTACATGAAATGGAGTCCAAGACTAAACCAGCACATGGAGGACAAGCAGATAATAAAAGCATGTCGA
APOSTART_3 (1143)   CCATATAACTCCAAGGATTCCTGTGATGGAAAACATGGTTGATCCATCGATGCCGAAAAGCCAGAAGCTACATGAAATGGAGTCCAAGACTAAACCAGCACATGGAGGACAAGCAGATAATAAAAGCATGTCGA
APOSTART_4 (1146)   CCATATAACTCCAAGGATTCCTGTGATGGAAAACATGGTTGATCCGTCAATGCCGAAAAACCAGAAGCTTCACGAAATGGAATCCAAGTCTAAGCCAGCACATGGAGGACAAACAGATAATAAGAGCATGTCGA
APOSTART_5 (1143)   CCATATAACTCCAAGAATTCCTGTGATGGAGAACATGGTTGATCCGTCAATGCCGAAAAACCAGAAGCTTCACGAAATGGAATCCAAGACTAAGCCAGCACATGGAGGACAAACAGATAATAAGAGCATGTCGA
APOSTART_6 (1143)   CCATATAACTCCAAGAATTCCTGTGATGGAGAACATGGTTGATCCGTCAATGCCGAAAAACCAGAAGCTTCACGAAATGGAATCCAAGACTAAGCCAGCACATGGAGGACAAACAGATAATAAGAGCATGTCGA
APOSTART 7 (1146)   CCATATAACTCCAAGGATTCCTGTGATGGAAAACATGGTTGATCCGTCGATGCTGAAAAGCCAGAAGCTGCATGAAATGGAGCCCAAGACTAAACCAGCACATGGAGGACAAGCAGATAATAAAAGCATGTCGA
APOSTART_8 (1119)   CCATATAACTCCAAGGATTCCTGTGATGGAAAACATGGTTGATCCATCAATGCCGAAAAGCCAGAAGCTACATGAAATGGAGTCCAAGACTAAACCAGCACATGGAGGACAAGCAGATAATAAAAGCATGTCGA
APOSTART_9 (1143)   CCATATAACTCCAAGGATTCCTGTGATGGAAAACATGGTTGATCCATCGATGCCGAAAAGCCAGAAGCTACATGAAATGGAGTCCAAGACTAAACCAGCACATGGAGGACAAGCAGATAATAAAAGCATGTCGA
APOSTART_10 (1128)  CCATATAACTCCAAGGATTCCTGTGATGGAAAACATGGTTGATCCATCGATGCCGAAAAGCCAGAAGCTACATGAAATGGAGTCCAAGACTAAACCAGCACATGGAGGACAAGCAGATAATAAAAGCATGTCGA
APOSTART_11 (1140)  CCATATAACTCCAAGGATTCCTGTGATGGAAAACATGGTTGATCCATCGATGCCGAAAAGCCAGAAGCTACATGAAATGGAGTCCAAGACTAAACCAGCACATGGAGGACAAGCAGATAATAAAAGCATGTCGA
APOSTART_12 (1146)  CCATATAACTCCAAGGATTCCTGTGATGGAAAACATGGTTGATCCGTCAATGCCGAAAAACCAGAAGCTTCACGAAATGGAATCCAAGTCTAAGCCAGCACATGGAGGACAAACAGATAATAAGAGCATGTCGA
APOSTART_14 (1116)  CCATATAACTCCAAGGATTCCTGTGATGGAAAACATGGTTGATCCGTCAATGCCGAAAAACCAGAAGCTTCACGAAATGGAATCCAAGTCTAAGCCAGCACATGGAGGACAAACAGATAATAAGAGCATGTCGA
APOSTART_15 (1212)  CCATATAACTCCAAGGATTCCTGTGATGGAAAACATGGTTGATCCGTCAATGCCGAAAAACCAGAAGCTTCACGAAATGGAATCCAAGTCTAAGCCAGCACATGGAGGACAAACAGATAATAAGAGCATGTCGA
APOSTART_13 (1116)  CCATATAACTCCAAGGATTCCTGTGATGGAAAACATGGTTGATCCGTCAATGCCGAAAAACCAGAAGCTTCACGAAATGGAATCCAAGTCTAAGCCAGCACATGGAGGACAAACAGATAATAAGAGCATGTCGA
APOSTART_1_GEN (3250)  CCATATAACTCCAAGGATTCCTGTGATGGAAAACATGGTTGATCCATCGATGCCGAAAAGCCAGAAGCTACATGAAATGGAGTCCAAGACTAAACCAGCACATGGAGGACAAGCAGATAATAAAAGCATGTCGA
APOSTART_2_GEN (3233)  CCATATAACTCCAAGGATTCCTGTGATGGAAAACATGGTTGATCCATCGATGCCGAAAAGCCAGAAGCTACATGAAATGGAGTCCAAGACTAAACCAGCACATGGAGGACAAGCAGATAATAAAAGCATGTCGA
APOSTART_5_GEN (3121)  CCATATAACTCCAAGAATTCCTGTGATGGAGAACATGGTTGATCCGTCAATGCCGAAAAACCAGAAGCTTCACGAAATGGAATCCAAGACTAAGCCAGCACATGGAGGACAAACAGATAATAAGAGCATGTCGA
APOSTART_6_GEN (3116)  CCATATAACTCCAAGAATTCCTGTGATGGAGAACATGGTTGATCCGTCAATGCCGAAAAACCAGAAGCTTCACGAAATGGAATCCAAGACTAAGCCAGCACATGGAGGACAAACAGATAATAAGAGCATGTCGA
APOSTART_7_GEN (3299)  CCATATAACTCCAAGGATTCCTGTGATGGAAAACATGGTTGATCCGTCGATGCTGAAAAGCCAGAAGCTGCATGAAATGGAGCCCAAGACTAAACCAGCACATGGAGGACAAGCAGATAATAAAAGCATGTCGA
APOSTART_8_GEN (3308)  CCATATAACTCCAAGGATTCCTGTGATGGAAAACATGGTTGATCCATCAATGCCGAAAAGCCAGAAGCTACATGAAATGGAGTCCAAGACTAAACCAGCACATGGAGGACAAGCAGATAATAAAAGCATGTCGA
APOSTART_10_GEN (3248) CCATATAACTCCAAGGATTCCTGTGATGGAAAACATGGTTGATCCATCGATGCCGAAAAGCCAGAAGCTACATGAAATGGAGTCCAAGACTAAACCAGCACATGGAGGACAAGCAGATAATAAAAGCATGTCGA
APOSTART_12_GEN (3264) CCATATAACTCCAAGGATTCCTGTGATGGAAAACATGGTTGATCCGTCAATGCCGAAAAACCAGAAGCTTCACGAAATGGAATCCAAGTCTAAGCCAGCACATGGAGGACAAACAGATAATAAGAGCATGTCGA
```

```
                                                                                                                                   Section 27
           (3485) 3485 3490      3500      3510      3520      3530      3540      3550      3560      3570      3580      3590      3600              3618
   APOSTART 1 (1277) TAATCGATGAAGAATCGGATGAGGATGATGACTATCAAGTTCCGGAAGCTAACATAGAGG--------------------------------------------------------------------------
   APOSTART 2 (1277) TAATCGATGAAGAGTCGGATGAGGATGATGACTATCAAGTTCCGGAAGCTAACATAGAGG--------------------------------------------------------------------------
   APOSTART_3 (1277) TAATCGATGAAGAATCGGATGAGGATGATGACTATCAAGTTCCGGAAGCTAACATAGAGG--------------------------------------------------------------------------
   APOSTART_4 (1280) TAATCGATGAAGAGTCAGATGAGGATGAGGACTATCAAGTTGCTGAAGCTAACATAGAGG--------------------------------------------------------------------------
   APOSTART_5 (1277) TAATCGATGAAGAGTCAGATGAGGATGAGGACTATCAAGTTGCTGAAGCTAACATAGAGG--------------------------------------------------------------------------
   APOSTART_6 (1277) TAATCGATGAAGAGTCAGATGAGGATGAGGACTATCAAGTTGCTGAAGCTAACATAGAGG--------------------------------------------------------------------------
   APOSTART 7 (1280) TAATCGATGAAGAGTCGGACGAGGATGATGACTATCAAGTTCCGGAAGCTAACATAGAGG--------------------------------------------------------------------------
   APOSTART_8 (1253) TAATCGATGAAGAATCGGATGAGGATGATGACTATCAAGTTCCGGAAGCTAACATAGAGG--------------------------------------------------------------------------
   APOSTART_9 (1277) TAATCGATGAAGAATCGGATGAGGATGATGACTATCAAGTTCCGGAAGCTAACATAGAGG--------------------------------------------------------------------------
  APOSTART_10 (1262) TAATCGATGAAGAATCGGATGAGGATGATGACTATCAAGTTCCGGAAGCTAACATAGAGG--------------------------------------------------------------------------
  APOSTART_11 (1274) TAATCGATGAAGAATCGGATGAGGATGATGACTATCAAGTTCCGGAAGCTAACATAGAGG--------------------------------------------------------------------------
  APOSTART_12 (1280) TAATCGATGAAGAGTCAGATGAGGATGAGGACTATCAAGTTGCTGAAGCTAACATAGAGG--------------------------------------------------------------------------
  APOSTART_14 (1250) TAATCGATGAAGAGTCAGATGAGGATGAGGACTATCAAGTTGCTGAAGCTAACATAGAGG--------------------------------------------------------------------------
  APOSTART_15 (1346) TAATCGATGAAGAGTCAGATGAGGATGAGGACTATCAAGTTGCTGAAGCTAACATAGAGG--------------------------------------------------------------------------
  APOSTART_13 (1250) TAATCGATGAAGAGTCAGATGAGGATGAGGACTATCAAGTTGCTGAAGCTAACATAGAGG--------------------------------------------------------------------------
 APOSTART_1_GEN (3384) TAATCGATGAAGAATCGGATGAGGATGATGACTATCAAGTTCCGGAAGCTAACATAGAGGTTACATATCCTGAGA--ACACACTATGAAGAACCAGATTTCATGATCAGTTTT-----------------ACAT
 APOSTART_2_GEN (3367) TAATCGATGAAGAGTCGGATGAGGATGATGACTATCAAGTTCCGGAAGCTAACATAGAGGTTACATATCCTGAGA--ACACACTGTGACGAACCAGATTTCATGATCAGTTTT-----------------ACAT
 APOSTART_5_GEN (3255) TAATCGATGAAGAGTCAGATGAGGATGAGGACTATCAAGTTGCTGAAGCTAACATAGAGGTTACATATCCTGAGACCACACACTATGAACACTCAGTTTTCCTTATCAATTTACGTCATACATTTTTTACACGA
 APOSTART_6_GEN (3250) TAATCGATGAAGAGTCAGATGAGGATGAGGACTATCAAGTTGCTGAAGCTAACATAGAGGTTACATATCCTGAGACCACACACTATGAGCACCCAGTTTTCCTTATCAATTTACGTCGTACAGTTTTTACACGA
 APOSTART_7_GEN (3433) TAATCGA-GAAGAGTCGGACGAGGATGATGACTATCAAGTTCCGGAAGCTAACATAGAGGTTACATATCCTGAGA--ACACACTATGAAGAACCAGATTTCATGATCAGTTTT-----------------ACAT
 APOSTART_8_GEN (3442) TAATCGATGAAGAATCGGATGAGGATGATGACTATCAAGTTCCGGAAGCTAACATAGAGGTTACATATCCTGAGA--ACACACTATGAAGAACCAGATTTCATGATCAGTTTT-----------------ACAT
APOSTART_10_GEN (3382) TAATCGATGAAGAATCGGATGAGGATGATGACTATCAAGTTCCGGAAGCTAACATAGAGGTTACATATCCTGAGA--ACACACTATGAAGAACCAGATTTCATGATCAGTTTT-----------------ACAT
APOSTART_12_GEN (3398) TAATCGATGAAGAGTCAGATGAGGATGAGGACTATCAAGTTGCTGAAGCTAACATAGAGGTTACATATCCTGAGA--ACACACT--GACGAACCAGATTTCATGATCAGTTTT-----------------ACAT
                                                                                                                                   Section 28
           (3619) 3619       3630      3640      3650      3660      3670      3680      3690      3700      3710      3720      3730      3740      3752
   APOSTART 1 (1337) ---------------------------------------------AAGACTCCAACAAGTCTGACAATGATGCTAAGCGTACAG--------------------------------------------------
   APOSTART 2 (1337) ---------------------------------------------AAGACTCCAACAAGTCTGACAATGATGCTAAGCGTACAG--------------------------------------------------
   APOSTART_3 (1337) ---------------------------------------------AAGACTCCAACAAGTCTGACAATGATGCTAAGCGTACAG--------------------------------------------------
   APOSTART_4 (1340) ---------------------------------------------AAGACTCCACCAAGTCTGACAATGATGCTAAGCGTACAG--------------------------------------------------
   APOSTART_5 (1337) ---------------------------------------------AAGACTCCAGTAAAGCTGACAACGATGCTAAGCGTACAG--------------------------------------------------
   APOSTART_6 (1337) ---------------------------------------------AAGACTCCAATAAAGCTGACAACGATGCTAAGCGTACAG--------------------------------------------------
   APOSTART 7 (1340) ---------------------------------------------AAGACTCCAACAAGTCTGACAATGATGCTAAGCGTGCAG--------------------------------------------------
   APOSTART_8 (1313) ---------------------------------------------AAGACTCCAACAAGTCTGACAATGATGCTAAGCGTACAG--------------------------------------------------
   APOSTART_9 (1337) ---------------------------------------------AAGACTCCAACAAGTCTGACAATGATGCTAAGCGTACAG--------------------------------------------------
  APOSTART_10 (1322) ---------------------------------------------AAGACTCCAACAAGTCTGACAATGATGCTAAGCGTACAG--------------------------------------------------
  APOSTART_11 (1334) ---------------------------------------------AAGACTCCAACAAGTCTGACAATGATGCTAAGCGTACAG--------------------------------------------------
  APOSTART_12 (1340) ---------------------------------------------AAGACTCCACCAAGTCTGACAATGATGCTAAGCGTACAG--------------------------------------------------
  APOSTART_14 (1310) ---------------------------------------------AAGACTCCACCAAGTCTGACAATGATGCTAAGCGTACAG--------------------------------------------------
  APOSTART_15 (1406) ---------------------------------------------AAGACTCCACCAAGTCTGACAATGATGCTAAGCGTACAG--------------------------------------------------
  APOSTART_13 (1310) ---------------------------------------------AAGACTCCACCAAGTCTGACAATGATGCTAAGCGTACAG--------------------------------------------------
 APOSTART_1_GEN (3499) GTTTTTTCACTTTCTATTAAACCATTTAAAAAATAAATGTACAGGAAGACTCCAACAAGTCTGACAATGATGCTAAGCGTACAGGTTTGATCCTTTCCGACAAATTACGTAAGCTGTCAAGTTCTTAGGATATA
 APOSTART_2_GEN (3482) GTTTTTTCACTTTCTATTAAACCATTTTAAAAATAAATGTACAGGAAGACTCCAACAAGTCTGACAATGATGCTAAGCGTACAGGTTTGATCCTTTCCGAAAAATTACGTCAGCTGTCAAGTTCTTAGGATAGA
 APOSTART_5_GEN (3389) TTTCTCACATCATA-ATAAACCTTTTATCAAAATTAATGTGCAGGAAGACTCCAGTAAAGCTGACAACGATGCTAAGCGTACAGGTTTGATTCCATGACACAAATAATGCCTGCTGTCAAGTGATGAGGATAGA
 APOSTART_6_GEN (3384) TTTCTTACATCATAAGTAAAACTTTTATCAAAATTAATGTGCAGGAAGACTCCAATAAAGCTGACAACGATGCTAAGCGTACAGGTTTTATTCCCTCACACAAATCATGCCTGATGTCAAGTGATGAGGATAGA
 APOSTART_7_GEN (3547) GTTTTTTCACTTTATATTAAATCTTTTTTAAA-TAAATGTACAGGAAGACTCCAACAAGTCTGACAATGATGCTAAGCGTACAGGTTTGATCCTTTCCGACAAATCACATCAGCTGTCAAGTTCTTAGGATAGA
 APOSTART_8_GEN (3557) GTTTTTTCACTTTATATTAAATCTTTTTTAAA-TAAATGTACAGGAAGACTCCAACAAGTCTGACAATGATGCTAAGCGTACAGGTTTGATCCTTTCCGACAAATCACATCAGCTGTCAAGTTCTTAGGATAGA
APOSTART_10_GEN (3497) GTTTTTTCACTTTCTATTAAACCATTTAAAAAATAAATGTACAGGAAGACTCCAACAAGTCTGACAATGATGCTAAGCGTACAGGTTTGATCCTTTCCGACAAATTACGTAAGCTGTCAAGTTCTTAGGATATA
APOSTART_12_GEN (3511) GTTTTTTCACTTTCTATTAAACCATTTAAAAAATAAATGTACAGGAAGACTCCACCAAGTCTGACAATGATGCTAAGCGTACAGGTTTGATCCTTTCCGACAAATTACGTCAGCTGTCAAGTTCTTAGGATAGA
```

Section 29

```
                    (3753) 3753      3760          3770          3780          3790          3800          3810          3820          3830          3840          3850          3860          3870          3886
       APOSTART 1 (1376) -----------------------------------------AAGAACCTCCAGAAAAAATTGATCTGTCTTGCTTTTCGGGCATCCTTCATCGTGACACGGATGAGAAAAGCCGTAACTATTGGACAGTACCCG
       APOSTART 2 (1376) -----------------------------------------AAGAACCTCCAGAAAAAATTGATCTGTCTTGCTTTTCGGGCATCCTTCATCGTGACACGGATGAGAAAAGCCGTAACTGTTGGACAGTACCCG
       APOSTART_3 (1376) -----------------------------------------AAGAACCTCCAGAAAAAATTGATCTGTCTTGCTTTTCGGGCATCCTTCATCGTGACACGGATGAGAAAAGCCGTAACTATTGGACAGTACCCG
       APOSTART_4 (1379) -----------------------------------------AAGAACCTCCGGAAAAAATTGATCTGTCTTGCTTTTCGGGCATCCTTCATCGTGACACGGATGAGAAAAGCCGTAACTGTTGGACAGTACCCG
       APOSTART_5 (1376) -----------------------------------------AAGAGCCTCCAGAAAAGATTGATTTGTCTTGCTTTTCGGGCATCCTTCATCGTGACCCGGATGAGAAAAGCCGCAACTGTTGGACAGTACCCG
       APOSTART_6 (1376) -----------------------------------------AAGAGCCTCCAGAAAAGATTGATTTGTCTTGCTTTTCGGGCATCCTTCATCATGACCCGGATGAGAAAAGCCGCAACTGTTGGACAGTACCGG
       APOSTART 7 (1379) -----------------------------------------AAGAACCTCCAGAAAAAATTGATCTGTCTTGCTTTTCGGGCATCCTTCATCGTGACACTGATGAGAAAAGCCGTAACTGTTGGACAGTACCTG
       APOSTART_8 (1352) -----------------------------------------AAGAACCTCCAGAAAAAATTGATCTGTCTTGCTTTTCGGGCATCCTTCATCGTGACACGGATGAGAAAAGCCGTAACTATTGGACAGTACCCG
       APOSTART_9 (1376) -----------------------------------------AAGAACCTCCAGAAAAAATTGATCTGTCTTGCTTTTCGGGCATCCTTCATCGTGACACGGATGAGAAAAGCCGTAACTATTGGACAGTACCCG
      APOSTART_10 (1361) -----------------------------------------AAGAACCTCCAGAAAAAATTGATCTGTCTTGCTTTTCGGGCATCCTTCATCGTGACACGGATGAGAAAAGCCGTAACTATTGGACAGTACCCG
      APOSTART_11 (1373) -----------------------------------------AAGAACCTCCAGAAAAAATTGATCTGTCTTGCTTTTCGGGCATCCTTCATCGTGACACGGATGAGAAAAGCCGTAACTATTGGACAGTACCCG
      APOSTART_12 (1379) -----------------------------------------AAGAACCTCCGGAAAAAATTGATCTGTCTTGCTTTTCGGGCATCCTTCATCGTGATACGGATGAGAAAAGCCGTAACTGTTGGACAGTACCCG
      APOSTART_14 (1349) -----------------------------------------AAGAACCTCCGGAAAAAATTGATCTGTCTTGCTTTTCGGGCATCCTTCATCGTGACACGGATGAGAAAAGCCGTAACTGTTGGACAGTACCCG
      APOSTART_15 (1445) -----------------------------------------AAGAACCTCCGGAAAAAATTGATCTGTCTTGCTTTTCGGGCATCCTTCATCGTGACACGGATGAGAAAAGCCGTAACTGTTGGACAGTACCCG
      APOSTART_13 (1349) -----------------------------------------AAGAACCTCCGGAAAAAATTGATCTGTCTTGCTTTTCGGGCATCCTTCATCGTGACACGGATGAGAAAAGCCGTAACTATTGGACAGTACCCG
  APOSTART_1_GEN (3633) TTTTATTTTTTCTTTAACGGCCGATATCTTTGCCGTGGCAGAAGAACCTCCAGAAAAAATTGATCTGTCTTGCTTTTCGGGCATCCTTCATCGTGACACGGATGAGAAAAGCCGTAACTATTGGACAGTACCCG
  APOSTART_2_GEN (3616) TTTTATTTTTTCTTTAACGGCCGATATCTTTGC-GTGGCAGAAGAACCTCCAGAAAAAATTGATCTGTCTTGCTTTTCGGGCATCCTTCATCGTGACACGGATGAGAAAAGCCGTAACTGTTGGACAGTACCCG
  APOSTART_5_GEN (3522) TGTTATTTTTTCTCTCTAACGCAATATATTATTTTTGGCAGAAGAGCCTCCAGAAAAGATTGATTTGTCTTGCTTTTCGGGCATCCTTCATCGTGACCCGGATGAGAAAAGCCGCAACTGTTGGACAGTACCCG
  APOSTART_6_GEN (3518) TGTTATTTTTTCTCTCTAACGCAATATATTATTTTTGGCAGAAGAGCCTCCAGAAAAGATTGATTTGTCTTGCTTTTCGGGCATCCTTCATCATGACCCGGATGAGAAAAGCCGCAACTGTTGGACAGTACCGG
  APOSTART_7_GEN (3680) TTTTATTTTTTCTTTAACGGCCGATATCTTTGCCATGGAAGAAGAACCTCCAGAAAAAATTGATCTGTCTTGCTTTTCGGGCATCCTTCATCGTGACACTGATGAGAAAAGCCGTAACTGTTGGACAGTACCTG
  APOSTART_8_GEN (3690) TTTTATTTTTTCTTTAACGGCCGATATCTTTGCCATGGAAGAAGAACCTCCAGAAAAAATTGATCTGTCTTGCTTTTCGGGCATCCTTCATCGTGACACGGATGAGAAAAGCCGTAACTATTGGACAGTACCCG
 APOSTART_10_GEN (3631) TTTTATTTTTTCTTTAACGGCCGATATCTTTGCCGTGGCAGAAGAACCTCCAGAAAAAATTGATCTGTCTTGCTTTTCGGGCATCCTTCATCGTGACACGGATGAGAAAAGCCGTAACTATTGGACAGTACCCG
 APOSTART_12_GEN (3645) TTTTATTTTTTCTTTAACGGCCGATATCTTTGC-ATGGCAGAAGAACCTCCGGAAAAAATTGATCTGTCTTGCTTTTCGGGCATCCTTCATCGTGATACGGATGAGAAAAGCCGTAACTGTTGGACAGTACCCG
```

Section 30

```
                    (3887) 3887         3900          3910          3920          3930          3940          3950          3960          3970          3980          3990          4000          4010      4020
       APOSTART 1 (1469) ATAGCACACTCTTTAAAGTCCGTAGCAAGAACTTCCCTACTGATAAATCAAAG---------------------------------------------------------------------------------
       APOSTART 2 (1469) ATAGCACACTCTTTAAAGTCCGTAGCAAGAACTTCCCTACTGATAAATCAAAG---------------------------------------------------------------------------------
       APOSTART_3 (1469) ATAGCACACTCTTTAAAGTCCGTAGCAAGAACTTCCCTACTGATAAATCAAAG---------------------------------------------------------------------------------
       APOSTART_4 (1472) ATAGCACACTCTTTAAAGTCCGTAGCAAGAACTTCCCTACTGACAAATCAAAG---------------------------------------------------------------------------------
       APOSTART_5 (1469) ATAGCACACTCTTTAAAGTCCGTAGCAAGAACTTCCCTACTGATAAATCAAAG---------------------------------------------------------------------------------
       APOSTART_6 (1469) ATAGCACGCTCTTTAAAGTCCGTAGCAAGAACTTCCCTACTGATAAATCAAAG---------------------------------------------------------------------------------
       APOSTART 7 (1472) ATAGCACACTCTTTAAAGTCCGTAGCAAGAACTTCCCTACTGATAAATCAAAG---------------------------------------------------------------------------------
       APOSTART_8 (1445) ATAGCACACTCTTTAAAGTCCGTAGCAAGAACTTCCCTACTGATAAATCAAAG---------------------------------------------------------------------------------
       APOSTART_9 (1469) ATAGCACACTCTTTAAAGTCCGTAGCAAGAACTTCCCTACTGATAAATCAAAG---------------------------------------------------------------------------------
      APOSTART_10 (1454) ATAGCACACTCTTTAAAGTCCGTAGCAAGAACTTCCCTACTGATAAATCAAAG---------------------------------------------------------------------------------
      APOSTART_11 (1466) ATAGCACACTCTTTAAAGTCCGTAGCAAGAACTTCCCTACTGATAAATCAAAG---------------------------------------------------------------------------------
      APOSTART_12 (1472) ATAGCACACTCTTTAAAGTCCGTAGCAAGAACTTCCCTACTGACAAATCAAAG---------------------------------------------------------------------------------
      APOSTART_14 (1442) ATAGCACACTCTTTAAAGTCCGTAGCAAGAACTTCCCTACTGACAAATCAAAG---------------------------------------------------------------------------------
      APOSTART_15 (1538) ATAGCACACTCTTTAAAGTCCGTAGCAAGAACTTCCCTACTGACAAATCAAAG---------------------------------------------------------------------------------
      APOSTART_13 (1442) ATAGCACACTCTTTAAAGTCCGTAGCAAGAACTTCCCTACTGACAAATCAAAG---------------------------------------------------------------------------------
  APOSTART_1_GEN (3767) ATAGCACACTCTTTAAAGTCCGTAGCAAGAACTTCCCTACTGATAAATCAAAGGTCTAAATCTTCCAATTTATGCTTAATTTATTATGAAGAATATCTGATGAAGAAATTCAGGACACTCACATT-AAAGTTGC
  APOSTART_2_GEN (3749) ATAGCACACTCTTTAAAGTCCGTAGCAAGAACTTCCCTACTGATAAATCAAAGGTCTAAATCTTCCAATTTATGCTTAATTTATTATGAAGAATATCTGATGAGTAAATTCAGGACACTCACATT-AAACTTGC
  APOSTART_5_GEN (3656) ATAGCACACTCTTTAAAGTCCGTAGCAAGAACTTCCCTACTGATAAATCAAAGGTCTAAATCTTCCAATTTATGCTTAGTCTACAATGAAGCATATTAGATAAATAAATTCAGGACACTGACATTTTAAGTTAT
  APOSTART_6_GEN (3652) ATAGCACGCTCTTTAAAGTCCGTAGCAAGAACTTCCCTACTGATAAATCAAAGGTCTAAATCTTCCGGTTTATGCTTAGTCTATAATGAAGCATATTAGAT-AAGAACTTCAGGACACTGACATTTTAAGTTAC
  APOSTART_7_GEN (3814) ATAGCACACTCTTTAAAGTCCGTAGCAAGAACTTCCCTACTGATAAATCAAAGGTCTAAATCTTCCAATTTATGCTTAATTTATTATGAAGAATATATGATGAAGAAATTCAGGACACTCACATT-AAAGTTGT
  APOSTART_8_GEN (3824) ATAGCACACTCTTTAAAGTCCGTAGCAAGAACTTCCCTACTGATAAATCAAAGGTCTAAATCTTCCAATTTATGCTTAATTTATTATGAAGAATATATGATGAAGAAATTCAGGACACTCACATT-AAAGTTGT
 APOSTART_10_GEN (3765) ATAGCACACTCTTTAAAGTCCGTAGCAAGAACTTCCCTACTGATAAATCAAAGGTCTAAATCTTCCAATTTATGCTTAATTTATTATGAAGAATATCTGATGAAGAAATTCAGGACACTCACAT--AAAGTTGC
 APOSTART_12_GEN (3778) ATAGCACACTCTTTAAAGTCCGTAGCAAGAACTTCCCTACTGACAAATCAAAGGTCTAAATCTTCCAATTTATGCTTAATTTATTATGAAGAATATCTGATGAATAAATTCAGGACACTCACATT-AAACTTGC
```

```
                                                                                                                          Section 31
(4021)                 4021     4030      4040      4050      4060      4070      4080      4090      4100      4110      4120      4130      4140          4154
APOSTART 1 (1522)      --ATACCTGCACCAAGTTATCTCATGGAGCTTGCCGCAATTGACTGGTTTAAGGACACCAAGCGTATGGATAATGTTGGCAGGCAGAAAGGTTGTGTTGCACAGGT----------------------------
APOSTART 2 (1522)      --ATACCTGCCCCAAGTTATCTCATGGAGCTTGCCGCAATTGACTGGTTTAAGGACACCAAGCGTATGGATAATGTTGGCAGGCAGAAAGGTTGTGTTGCACAGGT----------------------------
APOSTART_3 (1522)      --ATACCTGCACCAAGTTATCTCATGGAGCTTGCCGCAATTGACTGGTTTAAGGACACCAAGCGTATGGATAATGTTGGCAGGCAGAAAGGTTGTGTTGCACAGGT----------------------------
APOSTART_4 (1525)      --ATACCTGCACCAAGTTATCTCATGGAGCTTGGCGCGATTGACTGGTTTAAGGACACCAAGCGTATGGATAATGTTGGCAGGCAGAAAGGTTGTGTTGCACAGGT----------------------------
APOSTART_5 (1522)      --ATACCAGCACCAAGTTATCTCATGGAGCTCGCGGCGATTGACTGGTTTAAGGACACCAAGCGTATGGATAATGTCGGCAGGCAGAAAGGCTGTGTTGCTCAGGT----------------------------
APOSTART_6 (1522)      --ATACCAGCACCAAGTTATCTCATGGAGCTCGCGGCGATTGACTGGTTTAAGGACACCAAGCGTATGGATAATGTCGGCAGGCAGAAAGGCTGTGTTGCTCAGGT----------------------------
APOSTART 7 (1525)      --ATACCTGCACCAAGTTATCTCATGGAGCTTGCCGCAATTGACTGGTTTAAGGACACCAAGCGTATGGATAATGTTGGCAGGCAGAAAGGTTGCGTTGCACAGGT----------------------------
APOSTART_8 (1498)      --ATACCTGCACCAAGTTATCTCATGGAGCTTGCCGCAATTGACTGGTTTAAGGACACCAAGCGTATGGATAATGTTGGCAGGCAGAAAGGTTGTGTTGCACAGGT----------------------------
APOSTART_9 (1522)      --ATACCTGCACCAAGTTATCTCATGGAGCTTGCCGCAATTGACTGGTTTAAGGACACCAAGCGTATGGATAATGTTGGCAGGCAGAAAGGTTGTGTTGCACAGGT----------------------------
APOSTART_10 (1507)     --ATACCTGCACCAAGTTATCTCATGGAGCTTGCCGCAATTGACTGGTTTAAGGACACCAAGCGTATGGATAATGTTGGCAGGCAGAAAGGTTGTGTTGCACAGGT----------------------------
APOSTART_11 (1519)     --ATACCTGCACCAAGTTATCTCATGGAGCTTGCCGCAATTGACTGGTTTAAGGACACCAAGCGTATGGATAATGTTGGCAGGCAGAAAGGTTGTGTTGCACAGGT----------------------------
APOSTART_12 (1525)     --ATACCTGCACCAAGTTATCTCATGGAGCTTGCCGCGATTGACTGGTTTAAGGACACCAAGCGTATGGATAATGTTGGCAGGCAGAAAGGTTGTGTTGCACAGGT----------------------------
APOSTART_14 (1495)     --ATACCTGCACCAAGTTATCTCATGGAGCTTGGCGCGATTGACTGGTTTAAGGACACCAAGCGTATGGATAATGTTGGCAGGCAGAAAGGTTGTGTTGCACAGGT----------------------------
APOSTART_15 (1591)     --ATGCCTGCACCAAGTTATCTCATGGAGCTTGGCGCGATTGACTGGTTTAAGGACACCAAGCGTATGGATAATGTTGGCAGGCAGAAAGGTTGTGTTGCACAGGT----------------------------
APOSTART_13 (1495)     --ATACCTGCACCAAGTTATCTCATGGAGCTTGCCGCGATTGACTGGTTTAAGGACACCAAGCGTATGGATAATGTTGGCAGGCAGAAAGGTTGTGTTGCACAGGT----------------------------
APOSTART_1_GEN (3900)  AGATACCTGCACCAAGTTATCTCATGGAGCTTGCCGCAATTGACTGGTTTAAGGACACCAAGCGTATGGATAATGTTGGCAGGCAGAAAGGTTGTGTTGCACAGGTAAACTTGATAATTTCTAGAATATTTCCT
APOSTART_2_GEN (3882)  AGATACCTGCCCCAAGTTATCTCATGGAGCTTGCCGCAATTGACTGGTTTAAGGACACCAAGCGTATGGATAATGTTGGCAGGCAGAAAGGTTGTGTTGCACAGGTAAACTTGATAATTTCCAGTATATTTCCT
APOSTART_5_GEN (3790)  AGATACCAGCACCAAGTTATCTCATGGAGCTCGCGGCGATTGACTGGTTTAAGGACACCAAGCGTATGGATAATGTCGGCAGGCAGAAAGGCTGTGTTGCTCAGGTAAACATGATGACTTACTACAAATTTCTT
APOSTART_6_GEN (3785)  AGATACCAGCACCAAGTTATCTCATGGAGCTCGCGGCGATTGACTGGTTTAAGGACACCAAGCGTATGGATAATGTCGGCAGGCAGAAAGGCTGTGTTGCTCAGGTAAACATGATGACTCACCACAAATTTCTT
APOSTART_7_GEN (3947)  AGATACCTGCACCAAGTTATCTCA-GGAGCT-GCCGCAATTGACTGGTTTAAGGACACCAAGCGTATGGATAATGTTGGCAGGCAGAAAGGTTGCGTTGCACAGGTAAACTTGATAATTTCCAGA-TATTTCCT
APOSTART_8_GEN (3957)  AGATACCTGCACCAAGTTATCTCATGGAGCTTGCCGCAATTGACTGGTTTAAGGACACCAAGCGTATGGATAATGTTGGCAGGCAGAAAGGTTGTGTTGCACAGGTAAACTTGATAATTTCCAGA-TATTTCCT
APOSTART_10_GEN (3897) AGATACCTGCACCAAGTTATCTCATGGAGCTTGCCGCAATTGACTGGTTTAAGGACACCAAGCGTATGGATAATGTTGGCAGGCAGAAAGGTTGTGTTGCACAGGTAAACTTGATAATTTCTAGAATATTTCCT
APOSTART_12_GEN (3911) AGATACCTGCACCAAGTTATCTCATGGAGCTTGCCGCGATTGACTGGTTTAAGGACACCAAGCGTATGGATAATGTTGGCAGGCAGAAAGGTTGTGTTGCACAGGTAAACTTGATAATTTCCAGTATATTTCCT
                                                                                                                          Section 32
(4155)                 4155 4160      4170      4180      4190      4200      4210      4220      4230      4240      4250      4260      4270            4288
APOSTART 1 (1626)      --------------------------------------------------------------------------------------------------------------------------------------
APOSTART 2 (1626)      --------------------------------------------------------------------------------------------------------------------------------------
APOSTART_3 (1626)      --------------------------------------------------------------------------------------------------------------------------------------
APOSTART_4 (1629)      --------------------------------------------------------------------------------------------------------------------------------------
APOSTART_5 (1626)      --------------------------------------------------------------------------------------------------------------------------------------
APOSTART_6 (1626)      --------------------------------------------------------------------------------------------------------------------------------------
APOSTART 7 (1629)      --------------------------------------------------------------------------------------------------------------------------------------
APOSTART_8 (1602)      --------------------------------------------------------------------------------------------------------------------------------------
APOSTART_9 (1626)      --------------------------------------------------------------------------------------------------------------------------------------
APOSTART_10 (1611)     --------------------------------------------------------------------------------------------------------------------------------------
APOSTART_11 (1623)     --------------------------------------------------------------------------------------------------------------------------------------
APOSTART_12 (1629)     --------------------------------------------------------------------------------------------------------------------------------------
APOSTART_14 (1599)     --------------------------------------------------------------------------------------------------------------------------------------
APOSTART_15 (1695)     --------------------------------------------------------------------------------------------------------------------------------------
APOSTART_13 (1599)     --------------------------------------------------------------------------------------------------------------------------------------
APOSTART_1_GEN (4034)  AAT--TTATCTTATTCTTGTATTTATGCATTTTATTTTATTAAATATTATTTTCAAAAGATCTCATTAACATTGTTAATCTGACCATCCAATTTATGAGGCAGCATCTAGAATCTATATAGTACTATTTCTCCT
APOSTART_2_GEN (4016)  ATT--TTATCTTATTCTTGTATTTATGCATTTTATTTTCTTAAATATTATTTTCAAAAGATCTCATTAACATTGTGAATCTGACCATCCAATTTACGAGGCAGCATCTAAAATCTATATAGTACTATTTCTCCT
APOSTART_5_GEN (3924)  GGCGCTTATCTTATTCTTGTATTTTTGTATTTTCTTTTTGTAAATATCAATT-CAAAATATCTCATTAACATTCT----------------------------------GAATC--------------------
APOSTART_6_GEN (3919)  AGC--GTAAAAAATTATCTTTTTCTTGTAGTTT-----------------------------------------------------------------------------------------------------
APOSTART_7_GEN (4078)  ATT--TTATCTTATTCTTGTATTTGTGCATTTTATTTTCTTAAATATTATTTTCAAAATATCTCATTAACATTGTGAATCTGACACTCCAACTTATGAAGCAGCATCTAGAATCTATATACTAGTATTTCTCCT
APOSTART_8_GEN (4090)  ATT--TTATCTTATTCTTGTATTTGTGCATTTTATTTTCTTAAATATTATTTTCAAAATATCTCATTAACATTGTGAATCTGACACTCCAACTTATGAAGCAGCATCTAGAATCTATATACTAGTATTTCTCCT
APOSTART_10_GEN (4031) AAT--TTATCTTATTCTTGTATTTATGCATTTTATTTTATTAAATATTATTTTCAAAAGATCTCATTAACATTGTTAATCTGACCATCCAATTTATGAGGCAGCATCTAGAATCTATATAG-ACTATTTCTCCT
APOSTART_12_GEN (4045) ATT--TTATCTTATTCTTGTATTTATGCATTTTATTTTCTTAAATATTATTTTCAAAAGATCTCACTAACGTTGTGAATCTGACCATCCAATTTATGAGGCAGCATCTAGAATCTATATAGTACTATTTCTCCT
```

Section 33

```
                  (4289) 4289      4300      4310      4320      4330      4340      4350      4360      4370      4380      4390      4400      4410      4422
        APOSTART 1 (1626) --------------------------------------------------------------------------------------------------------------------------------------
        APOSTART 2 (1626) --------------------------------------------------------------------------------------------------------------------------------------
        APOSTART_3 (1626) --------------------------------------------------------------------------------------------------------------------------------------
        APOSTART_4 (1629) --------------------------------------------------------------------------------------------------------------------------------------
        APOSTART_5 (1626) --------------------------------------------------------------------------------------------------------------------------------------
        APOSTART_6 (1626) --------------------------------------------------------------------------------------------------------------------------------------
        APOSTART 7 (1629) --------------------------------------------------------------------------------------------------------------------------------------
        APOSTART_8 (1602) --------------------------------------------------------------------------------------------------------------------------------------
        APOSTART_9 (1626) --------------------------------------------------------------------------------------------------------------------------------------
       APOSTART_10 (1611) --------------------------------------------------------------------------------------------------------------------------------------
       APOSTART_11 (1623) --------------------------------------------------------------------------------------------------------------------------------------
       APOSTART_12 (1629) --------------------------------------------------------------------------------------------------------------------------------------
       APOSTART_14 (1599) --------------------------------------------------------------------------------------------------------------------------------------
       APOSTART_15 (1695) --------------------------------------------------------------------------------------------------------------------------------------
       APOSTART_13 (1599) --------------------------------------------------------------------------------------------------------------------------------------
    APOSTART_1_GEN (4166) GTCGAGCAAGACCAGAACATAACAGTCTTTTTTA-ATGAAAAGTGGCGCAGCCGCATCGTGCTTCACTATAGTACAAATTATCGTGTTAATAGCTCTATTTTTTTTTATTGAATGCTTAAGAGAGGTTCACGAT
    APOSTART_2_GEN (4148) GTCGAGCAAGACCAGAACATAACAGTCTTTTTTT-ATGAAAAGTGGCACAGCCGCATCGTGCTTCGCTATAGTACAAATTATCGTGTTGATAGCTCTAATTTTTTG-ATTGATTGCTTAAGAGAGATTCATGAT
    APOSTART_5_GEN (4003) ------------------------------------------------------------------------------------------GATG----------------------------------------
    APOSTART_6_GEN (3950) -------------------------------------------------------------TTGCATTTTCTTTGTAAATATCATTTCAATATATCTAATT---------------------------------
    APOSTART_7_GEN (4210) GTCGAGCAAGACCAGAACATAACAGTCTTTTTTTTATGAAAAGTGGCACAGCTGCATCGTGCTTCGCTATAGTACAAATTATCGTGTTGATAGCTCTAATTTTTTG-ATTGAATGCTTAAGAGAGGTTCACGAT
    APOSTART_8_GEN (4222) GTCGAGCAAGACCAGAACATAACAGTCTTTTTTTTATGAAAAGTGGCACAGCTGCATCGTGCTTCGCTATAGTACAAATTATCGTGTTGATAGCTCTAATTTTTTG-ATTGAATGCTTAAGAGAGGTTCACGAT
   APOSTART_10_GEN (4162) GTCGAGCAAGACCAGAACATAACAGTCTTTTTTAATGAAAAGTGGCGCAGCCGCATCGTGCTTCACTATAGTACAAATTATCGTGTTAATAGCTCTATTTTTTTTT-ATTGAATGCTTAAGAGAGGTTCACGAT
   APOSTART_12_GEN (4177) GTCGAGCAAGACCAGAACATAATAGTCTTTTTTT-ATGAAAAGTGGCACAGCCGCATCGTGCTTCGCTATTGTACAAATTATCGTGTTGATAGCTCTAATTTTTTG-ATTGAATGCTTAAGAGAGGTTCACGAT
```

Section 34

```
                  (4423) 4423   4430      4440      4450      4460      4470      4480      4490      4500      4510      4520      4530      4540           4556
        APOSTART 1 (1626) --------------------------------------------------------------------------------------------------------------------------------------
        APOSTART 2 (1626) --------------------------------------------------------------------------------------------------------------------------------------
        APOSTART_3 (1626) --------------------------------------------------------------------------------------------------------------------------------------
        APOSTART_4 (1629) --------------------------------------------------------------------------------------------------------------------------------------
        APOSTART_5 (1626) --------------------------------------------------------------------------------------------------------------------------------------
        APOSTART_6 (1626) --------------------------------------------------------------------------------------------------------------------------------------
        APOSTART 7 (1629) --------------------------------------------------------------------------------------------------------------------------------------
        APOSTART_8 (1602) --------------------------------------------------------------------------------------------------------------------------------------
        APOSTART_9 (1626) --------------------------------------------------------------------------------------------------------------------------------------
       APOSTART_10 (1611) --------------------------------------------------------------------------------------------------------------------------------------
       APOSTART_11 (1623) --------------------------------------------------------------------------------------------------------------------------------------
       APOSTART_12 (1629) --------------------------------------------------------------------------------------------------------------------------------------
       APOSTART_14 (1599) --------------------------------------------------------------------------------------------------------------------------------------
       APOSTART_15 (1695) --------------------------------------------------------------------------------------------------------------------------------------
       APOSTART_13 (1599) --------------------------------------------------------------------------------------------------------------------------------------
    APOSTART_1_GEN (4299) ATTATAACTTTGAAATCATTTAGTACACTGAGAACAAGTATTACAGAAATTTAGCCAAATGTTTCAGATACCAATCAGAAACTCATGCTTGTTTCTGTCTGAAGTGTAAGACAAGAAAACTGAGTCATTGTTCA
    APOSTART_2_GEN (4280) ATTATAACTTTCAAATCATTTAGTACACTGAGAACAAGTATTACAGAAATTTAGCCAAATGTTTCAGATACCAATCAGAAACTCATGCTTG----------AAGTGTAAGACAAGAAAACTGAGTCATTGTTCA
    APOSTART_5_GEN (4007) --------------------------------------------------T------------------------------------------TCTATCTGAAGAATAAGATAATAAAACTGAGCCCTTGTTCA
    APOSTART_6_GEN (3990) ---------------------------------------------------------------------AACATTCGGAATCTC-----------TGTTTCTATCTGAAGAGAATAAAGCTGAGCCCTTGTTCA
    APOSTART_7_GEN (4343) ATTATAACTTTGAAATCATTTAGTACACTGAGAACAAGTATTACAGAAATTTAGCCAAATGTTTCAGATACCAATCAGAAACTCATGCTTGTTTTTGTATGAAGTGTAAGACAAGAAAACTGAGTCATTGTTCA
    APOSTART_8_GEN (4355) ATTATAACTTTGAAATCATTTAGTACACTGAGAACAAGTATTACAGAAATTTAGCCAAATGTTTCAGATACCAATCAGAAACTCATGCTTGTTTTTGTATGAAGTGTAAGACAAGAAAACTGAGTCATTGTTCA
   APOSTART_10_GEN (4295) ATTATAACTTTGAAATCATTTAGTACACTGAGAACAAGTATTACAGAAATTTAGCCAAATGTTTCAGATACCAATCAGAAACTCATGCTTGTTTCTGTCTGAAGTGTAAGACAAGAAAACTGAGTCATTGTTCA
   APOSTART_12_GEN (4309) ATTATAACTTTGAAATCATTTAGTACACTGAGAACAAGTATTACAGAAATTTAGCCAAATGTTTCAGACACCAATCAGAAACTCATGCTTGTTTCTGTCTGAAGTGTAAGACAAGAAAACTGAGTCATTGTTCA
```

Section 35

```
                      (4557) 4557         4570      4580      4590      4600      4610      4620      4630      4640      4650      4660      4670      4680      4690
         APOSTART 1 (1626) ----------------------------------TGCTGCTGAGAAAGGGATGCACACATTTGTTGCCAACATACAG---------------------------------------------------------
         APOSTART 2 (1626) ----------------------------------TGCTGCTGAGAAAGGGATGCACACATTTGTTGCCAACATACAG---------------------------------------------------------
         APOSTART_3 (1626) ----------------------------------TGCTGCTGAGAAAGGGATGCACACATTTGTTGCCAACATACAG---------------------------------------------------------
         APOSTART_4 (1629) ----------------------------------TGCTGCTGAGAAAGGGATGCACACATTTGTTGCCAACATACAG---------------------------------------------------------
         APOSTART_5 (1626) ----------------------------------TGCCGCTGAGAAAGGAATGCACACATTTGTTGCCAACATACAG---------------------------------------------------------
         APOSTART_6 (1626) ----------------------------------TGCCGCTGAGAAAGGAATGCACACATTTGTTGCCAACATACAG---------------------------------------------------------
         APOSTART 7 (1629) ----------------------------------TGCTGCTGAGAAAGGGATGCACACATTTGTTGCCAACATACAG---------------------------------------------------------
         APOSTART_8 (1602) ----------------------------------TGCTGCTGAGAAAGGGATGCACACATTTGTTGCCAACATACAG---------------------------------------------------------
         APOSTART_9 (1626) ----------------------------------TGCTGCTGAGAAAGGGATGCACACATTTGTTGCCAACATACAG---------------------------------------------------------
        APOSTART_10 (1611) ----------------------------------TGCTGCTGAGAAAGGGATGCACACATTTGTTGCCAACATACAG---------------------------------------------------------
        APOSTART_11 (1623) ----------------------------------TGCTGCTGAGAAAGGGATGCACACATTTGTTGCCAACATACAG---------------------------------------------------------
        APOSTART_12 (1629) ----------------------------------TGCTGCTGAGAAAGGGATGCACACATTTGTTGCCAACATACAG---------------------------------------------------------
        APOSTART_14 (1599) ----------------------------------TGCTGCTGAGAAAGGGATGCACACATTTGTTGCCAACATACAG---------------------------------------------------------
        APOSTART_15 (1695) ----------------------------------TGCTGCTGAGAAAGGGATGCACACATTTGTTGCCAACATACAG---------------------------------------------------------
        APOSTART_13 (1599) ----------------------------------TGCTGCTGAGAAAGGGATGCACACATTTGTTGCCAACATACAG---------------------------------------------------------
    APOSTART_1_GEN (4433) TAATTTCATTGCTCATATTACCTGCTTCGTAGGTTGCTGCTGAGAAAGGGATGCACACATTTGTTGCCAACATACAGGTGAGCTATTCCGCTATAAATTTCATTGTACTGTTATTCCTGCATTTGAAGACTGGT
    APOSTART_2_GEN (4404) TAATTTCATTGCTCA-----CCTGCTTTGTAGGTTGCTGCTGAGAAAGGGATGCACACATTTGTTGCCAACATACAGGTGAGCTATTCCGCTATAAATTTCATTGTACTGTTATTCCTGCGTTTGAAGACTGGT
    APOSTART_5_GEN (4049) TACTT-CATTGCTCATGTTACTGTCTTTGTAGGTTGCCGCTGAGAAAGGAATGCACACATTTGTTGCCAACATACAGGCGAGTTATTCTGCGATAGATATCTTTGTAATATTATTCATGCGTTTGAAGGCTAGT
    APOSTART_6_GEN (4044) TAATTTCATTGCTCATGTTACTGGCTTTGTAGGTTGCCGCTGAGAAAGGAATGCACACATTTGTTGCCAACATACAGGCGAGTTATTCTGTGATAGATTTGTTTGAAATATTATTAATGTGTTTGTAGAATAGT
    APOSTART_7_GEN (4477) AAATTTAATTGCTCATGTTACCTGCTTTGTAGGTTGCTGCTGAGAAAGGGATGCACACATTTGTTGCCAACATACAGGTGAGCTATTCCGCTATAAATTTCATTGTACTGTTATTCATGCGTTTGAAGACTGGT
    APOSTART_8_GEN (4489) AAATTTAATTGCTCATGTTACCTGCTTTGTAGGTTGCTGCTGAGAAAGGGATGCACACATTTGTTGCCAACATACAGGTGAGCTATTCCGCTATAAATTTCATTGTACTGTTATTCATGCGTTTGAAGACTGGT
   APOSTART_10_GEN (4429) TAATTTCATTGCTCATATTACCTGCTTCGTAGGTTGCTGCTGAGAAAGGGATGCACACATTTGTTGCCAACATACAGGTGAGCTATTCCGCTATAAATTTCATTGTACTGTTATTCCTGCATTTGAAGACTGGT
   APOSTART_12_GEN (4443) TAATTTCATTGCTCATATTACCTGCTTTGTAGGTTGCTGCTGAGAAAGGGATGCACACATTTGTTGCCAACATACAGGTGAGCTATTCCGCTATCAATTTCATTGTACTGTTATTCCTGCGTTTGAAAACTGGT
```

Section 36

```
                      (4691) 4691     4700      4710      4720      4730      4740      4750      4760      4770      4780      4790      4800      4810         4824
         APOSTART 1 (1669) ------------------------------------------------------------------------ATTCCGGGATCAACTCATTACAGCTTGGTGATGTATTTCGTCACGAGTTGCATGAAAAAAGG
         APOSTART 2 (1669) ------------------------------------------------------------------------ATTCCTGGATCAACTCATTACAGCTTGGTGATGTATTTCGTCACGAGTTCCATGAAAAAAGG
         APOSTART_3 (1669) ------------------------------------------------------------------------ATTCCGGGATCAACTCATTACAGCTTGGTGATGTATTTCGTCACGAGTTGCATGAAAAAAGG
         APOSTART_4 (1672) ------------------------------------------------------------------------ATTCCCGGATCAACTCATTACAGCTTGGTGATGTATTTCGTCACGAGTTCCATGAAAAAAGG
         APOSTART_5 (1669) ------------------------------------------------------------------------ATTCCTGGATCAACTCATTACAGCTTGGTGATGTATTTCGTCACGAGTACCATGAAAAAAGG
         APOSTART_6 (1669) ------------------------------------------------------------------------ATTCCTGGATCAACTCATTACAGCTTGGTGATGTATTTCGTCACGAGTACCATGAAAAAGGG
         APOSTART 7 (1672) ------------------------------------------------------------------------ATTCCTGGATCAACTCATTACAGCTTGGTGATGTATTTCGTCACGAGTTCCATGAAAAAAGG
         APOSTART_8 (1645) ------------------------------------------------------------------------ATTCCTGGATCAACTCATTACAGCTTGGTGATGTATTTCGTCACGAGTTGCATGAAAAAAGG
         APOSTART_9 (1669) ------------------------------------------------------------------------ATTCCTGGATCAACTCATTACAGCTTGGTGATGTATTTCGTCACGAGTTGCATGAAAAAAGG
        APOSTART_10 (1654) ------------------------------------------------------------------------ATTCCTGGATCAACTCATTACAGCTTGGTGATGTATTTCGTCACGAGTTGCATGAAAAAAGG
        APOSTART_11 (1666) ------------------------------------------------------------------------ATTCCTGGATCAACTCATTACAGCTTGGTGATGTATTTCGTCACGAGTTGCATGAAAAAAGG
        APOSTART_12 (1672) ------------------------------------------------------------------------ATTCCCGGATCAACTCATTACAGCTTGGTGATGTATTTCGTCACGAGTTCCATGAAAAAAGG
        APOSTART_14 (1642) ------------------------------------------------------------------------ATTCCCGGATCAACTCATTACAGCTTGGTGATGTATTTCGTCACGAGTTCCATGAAAAAAGG
        APOSTART_15 (1738) ------------------------------------------------------------------------ATTCCCGGATCAACTCATTACAGCTTGGTGATGTATTTCGTCACGAGTTCCATGAAAAAAGG
        APOSTART_13 (1642) ------------------------------------------------------------------------ATTCCCGGATCAACTCATTACAGCTTGGTGATGTATTTCGTCACGAGTTCCATGAAAAAAGG
    APOSTART_1_GEN (4567) ATATATCCAACTTTTTTTTTCGAAAGGAAAGA-TTTTTTTGGGAAGTTAATTAACTTAATATCCTATTGCAGATTCCGGGATCAACTCATTACAGCTTGGTGATGTATTTCGTCACGAGTTGCATGAAAAAAGG
    APOSTART_2_GEN (4533) ATATATCAAACTTTTTTTTTCGAAAGGAAAGA-TTTTTTGGTGAAGTTAATTAACTTAATATCCTATTGCAGATTCCTGGATCAACTCATTACAGCTTGGTGATGTATTTCGTCACGAGTTCCATGAAAAAAGG
    APOSTART_5_GEN (4182) GTT----GAACTTTTTTCCAAAAGGAAACA----TGTTTGGTGAAGTTAAT---------ATCCCATTGCAGATTCCTGGATCAACTCATTACAGCTTGGTGATGTATTTCGTCACGAGTACCATGAAAAAAGG
    APOSTART_6_GEN (4178) GTT----GAACTTTTTTCCAAAATGAAACA----TGTTTGGTGAAGTTAAT---------ATCCCATTGCAGATTCCTGGATCAACTCATTACAGCTTGGTGATGTATTTCGTCACGAGTACCATGAAAAAGGG
    APOSTART_7_GEN (4611) ATATATCAAACTTTTTTTTGAAAGGAGAGA---TTTTTTGGTGAAGTTAATTAACTTAATATCCTATTGCAGATTCCTGGATCAACTCATTACAGCTTGGTGATGTATTTCGTCACGAGTTCCATGAAAAAAGG
    APOSTART_8_GEN (4623) ATATATCAAACTTTTTTTTGAAAGGAGAGA---TTTTTTGKKGAAGTTAATTAACTTAATATCCTATTGCAGATTCCTGGATCAACTCATTACAGCTTGGTGATGTATTTCGTCACGAGTTGCATGAAAAAAGG
   APOSTART_10_GEN (4563) ATATATCCAACTTTTTTTTTCGAAAGGAAAGATTTTTTTTGGTGAAGTTAATTAACTTAATATCCTATTGCAGATTCCTGGATCAACTCATTACAGCTTGGTGATGTATTTCGTCACGAGTTGCATGAAAAAAGG
   APOSTART_12_GEN (4577) ATATATCAAACTTTTTTTTGAAAGGAGAGA---TTTTTTGGTGAAGTTAATTAACTTAATATCCTATTGCAGATTCCCGGATCAACTCATTACAGCTTGGTGATGTATTTCGTCACGAGTTCCATGAAAAAAGG
```

Section 37

```
                   (4825) 4825      4830      4840      4850      4860      4870      4880      4890      4900      4910      4920      4930      4940            4958
      APOSTART 1 (1731) ATCATTGCTGCAACGTTTCTTTGACGGAGATGATGAATTCCGCAATAGCAGACTAAAGCTTATACCAGCCGTTCCAAAGG------------------------------------------------------
      APOSTART 2 (1731) ATCATTGCTGCAACGTTTCTTTGATGGAGATGATGAATTCCGCAATAGCAGACTAAAGCTTATACCGGCCGTTCCAAAGG------------------------------------------------------
      APOSTART_3 (1731) ATCATTGCTGCAACGTTTCTTTGACGGAGATGATGAATTCCGCAATAGCAGACTAAAGCTTATACCAGCCGTTCCAAAGG------------------------------------------------------
      APOSTART_4 (1734) ATCATTGCTGCAACGTTTCTTTGACGGAGATGATGAATTCCGCAATAGCAGACTGAAGCTTATACCGGCCGTTCCAAAGG------------------------------------------------------
      APOSTART_5 (1731) ATCATTGCTGCAACGTTTCTTTGATGGAGATGATGAATTCCGCAATAGTAGACTGAAGCTTATACCGGCGGTTCCAAAGG------------------------------------------------------
      APOSTART_6 (1731) ATCATTGCTGCAACGTTTCTTCGACGGAGATGATGAATTCCGCAATAGTAGACTCAAGCTTATACCGGCGGTTCCAAAGG------------------------------------------------------
      APOSTART 7 (1734) ATCATTGCTGCAACGTTTCTTTGACGGAGATGATGAATTCCGCAATAGCAGACTGAAGCTTATACCAGCCGTTCCAAAGG------------------------------------------------------
      APOSTART_8 (1707) ATCATTGCTGCAACGTTTCTTTGACGGAGATGATGAATTCCGCAATAGCAGACTAAAGCTTATACCAGCCGTTCCAAAGG------------------------------------------------------
      APOSTART_9 (1731) ATCATTGCTGCAACGTTTCTTTGACGGAGATGATGAATTCCGCAATAGCAGACTAAAGCTTATACCAGCCGTTCCAAAGG------------------------------------------------------
     APOSTART_10 (1716) ATCATTGCTGCAACGTTTCTTTGACGGAGATGATGAATTCCGCAATAGCAGACTAAAGCTTATACCAGCCGTTCCAAAGG------------------------------------------------------
     APOSTART_11 (1728) ATCATTGCTGCAACGTTTCTTTGACGGAGATGATGAATTCCGCAATAGCAGACTAAAGCTTATACCAGCCGTTCCAAAGG------------------------------------------------------
     APOSTART_12 (1734) ATCATTGCTGCAACGTTTCTTTGACGGAGATGATGAATTCCGCAATAGCAGACTGAAGCTTATACCGGCCGTTCCAAAGG------------------------------------------------------
     APOSTART_14 (1704) ATCATTGCTGCAACGTTTCTTTGACGGAGATGATGAATTCCGCAATAGCAGACTGAAGCTTATACCGGCCGTTCCAAAGG------------------------------------------------------
     APOSTART_15 (1800) ATCATTGCTGCAACGTTTCTTTGACGGAGATGATGAATTCCGCAATAGCAGACTGAAGCTTATACCGGCCGTTCCAAAGG------------------------------------------------------
     APOSTART_13 (1704) ATCATTGCTGCAACGTTTCTTTGACGGAGATGATGAATTCCGCAATAGCAGACTGAAGCTTATACCGGCCGTTCCAAAGG------------------------------------------------------
 APOSTART_1_GEN (4700) ATCATTGCTGCAACGTTTCTTTGACGGAGATGATGAATTCCGCAATAGCAGACTAAAGCTTATACCAGCCGTTCCAAAGGTCTGTTTTTTTTCTTCATAAACTCATGTGTGGGATGCAAAAAAAAAAAAAATCT
 APOSTART_2_GEN (4666) ATCATTGCTGCAACGTTTCTTTGATGGAGATGATGAATTCCGCAATAGCAGACTAAAGCTTATACCGGCCGTTCCAAAGGTCTGTTTTTTTCTTCATAAACTCATGTGGGATGCAAAAAAAAAAAAATTACTCT
 APOSTART_5_GEN (4299) ATCATTGCTGCAACGTTTCTTTGATGGAGATGATGAATTCCGCAATAGTAGACTGAAGCTTATACCGGCGGTTCCAAAGGTCTGCTTTTTTCAATAAAAA---CATGTACGCTGCAACTATTATGTTGTACTCT
 APOSTART_6_GEN (4295) ATCATTGCTGCAACGTTTCTTCGACGGAGATGATGAATTCCGCAATAGTAGACTCAAGCTTATACCGGCGGTTCCAAAGGTATGTTTTTTCCCATAAAAACATGTAGGGT--------------------GATG
 APOSTART_7_GEN (4742) ATCATTGCTGCAACGTTTCTTTGACGGAGATGATGAATTCCGCAATAGCAGACTGAAGCTTATACCAGCCGTTCCAAAGGTCTTTTTTTTTTCTTCATAAAACTCATGTG--GGATGCAAAAAAAAATTTACTTT
 APOSTART_8_GEN (4754) ATCATTGCTGCAACGTTTCTTTGACGGAGATGATGAATTCCGCAATAGCAGACTAAAGCTTATACCAGCCGTTCCAAAGGTCTTTTTTTTTTCTTCATAAAACTCATGTG--GGATGCAAAAAAAAATTTACTTT
APOSTART_10_GEN (4697) ATCATTGCTGCAACGTTTCTTTGACGGAGATGATGAATTCCGCAATAGCAGACTAAAGCTTATACCAGCCGTTCCAAAGGTCTGTTTTTTTTCTTCATAAACTCATGTGT-GGGATGCAAAAAAAAAAAAATCT
APOSTART_12_GEN (4708) ATCATTGCTGCAACGTTTCTTTGACGGAGATGATGAATTCCGCAATAGCAGACTGAAGCTTATACCGGCCGTTCCAAAGGTCTTTTTTTTTTCTTCATAAAACTCATGTG--GGATGCAAAAAAAAATTTACTTT
```

Section 38

```
                   (4959) 4959       4970      4980      4990      5000      5010      5020      5030      5040      5050      5060      5070      5080        5092
      APOSTART 1 (1811) ---------------------------------------------GCTCATGGATAGTGCGGCAGAGCGTTGGAAGTACCCCTTGTTTATTGGGAAAGGCGGTCGATTGCAGCTACGTGCGTGGTCCAGGTTAC
      APOSTART 2 (1811) ---------------------------------------------GCTCATGGATAGTGCGGCAGAGCGTTGGAAGTACCCCTTGTTTATTGGGAAAGGCGGTCGATTGCAGCTACGTGCGTGGTCCAGGTTAC
      APOSTART_3 (1811) ---------------------------------------------GCTCATGGATAGTGCGGCAGAGCGTTGGAAGTACCCCTTGTTTATTGGGAAAGGCGGTCGATTGCAGCTACGTGCGTGGTCCAGGTTAC
      APOSTART_4 (1814) ---------------------------------------------GCTCATGGATAGTGCGGCAGAGCGTTGGAAGTACCCCTTGTTTATTGGGAAAGGCGGTTGATTGCAGCTACGTGCGTGGTCCGGGTTAC
      APOSTART_5 (1811) ---------------------------------------------GATCTTGGATAGTGCGGCAGAGCGTTGGGAGTACCCCTTGTTTATTGGGAAAGGCCGTCGATTGCAGCTACGTGCGTGGTCCAGGGTAC
      APOSTART_6 (1811) ---------------------------------------------GATCTTGGATAGTGCGGCAGAGCGTCGGGAGTACCCCTTGTTTATTGGGAAAGGCCGTCGATTGCAGCTACGTGCGTGGTCCAGGTTAC
      APOSTART 7 (1814) ---------------------------------------------GCTCATGGATAGTGCGGCAGAGCGTTGGAAGTACCCCTTGTTTATTGGGAAAGGCGGTCGATTGCAGCTACGTGCGTGGTCCGGGTTAC
      APOSTART_8 (1787) ---------------------------------------------GCTCATGGATAGTGCGGCAGAGCGTTGGAAGTACCCCTTGTTTATTGGGAAAGGCAGTCGATTGCAGCTACGTGCGTGGTCCAGGTTAC
      APOSTART_9 (1811) ---------------------------------------------GCTCATGGATAGTGCGGCAGAGCGTTGGAAGTACCCCTTGTTTATTGGGAAAGGCAGTCGATTGCAGCTACGTGCGTGGTCCAGGTTAC
     APOSTART_10 (1796) ---------------------------------------------GCTCATGGATAGTGCGGCAGAGCGTTGGAAGTACCCCTTGTTTATTGGGAAAGGCAGTCGATTGCAGCTACGTGCGTGGTCCAGGTTAC
     APOSTART_11 (1808) ---------------------------------------------GCTCATGGATAGTGCGGCAGAGCGTTGGAAGTACCCCTTGTTTATTGGGAAAGGCAGTCGATTGCAGCTACGTGCGTGGTCCAGGTTAC
     APOSTART_12 (1814) ---------------------------------------------GCTCATGGATAGTGCGGCAGAGCGTTGGAAGTACCCCTTGTTTATTGGGAAAGGCGGTTGATTGCAGCTACGTGCGTGGTCCGGGTTAC
     APOSTART_14 (1784) ---------------------------------------------GCTCATGGATAGTGCGGCAGAGCGTTGGAAGTACCCCTTGTTTATTGGGAAAGGCGGTTGATTGCAGCTACGTGCGTGGTCCGGGTTAC
     APOSTART_15 (1880) ---------------------------------------------GCTCATGGATAGTGCGGCAGAGCGTTGGAAGTACCCCTTGTTTATTGGGAAAGGCGGTTGATTGCAGCTACGTGCGTGGTCCGGGTTAC
     APOSTART_13 (1784) ---------------------------------------------GCTCATGGATAGTGCGGCAGAGCGTTGGAAGTACCCCTTGTTTATTGGGAAAGGCGGTTGATTGCAGCTACGTGCGTGGTCCGGGTTAC
 APOSTART_1_GEN (4834) TCATGTCAACTCTGAAACAACGAACAACAAAAATAAATCTTCAGGGCTCATGGATAGTGCGGCAGAGCGTTGGAAGTACCCCTTGTTTATTGGGAAAGGCGGTCGATTGCAGCTACGTGCGTGGTCCAGGTTAC
 APOSTART_2_GEN (4800) TCATGTCAACTTTGACACAACGAACAACAAAAATAAATCTTCAGGGCTCATGGATAGTGCGGCAGAGCGTTGGAAGTACCCCTTGTTTATTGGGAAAGGCGGTCGATTGCAGCTACGTGCGTGGTCCAGGTTAC
 APOSTART_5_GEN (4430) TCAGCTTCACTCTGACTCCACAAACAA---ATAAAAATTGTCAGGGATCTTGGATAGTGCGGCAGAGCGTTGGGAGTACCCCTTGTTTATTGGGAAAGGCCGTCGATTGCAGCTACGTGCGTGGTCCAGGGTAC
 APOSTART_6_GEN (4409) TACTCTTCACTCTGACTCCACAGACAA-ACAAAAAAATCGTCAGGGATCTTGGATAGTGCGGCAGAGCGTCGGGAGTACCCCTTGTTTATTGGGAAAGGCCGTCGATTGCAGCTACGTGCGTGGTCCAGGTTAC
 APOSTART_7_GEN (4874) TCATGTCAACTTTGACACAACGAACAACAAAAATAAATCTTCAGGGCTCATGGATAGTGCGGCAGAGCGTTGGAAGTACCCCTTGTTTATTGGGAAAGGCGGTCGATTGCAGCTACGTGCGTGGTCCGGGTTAC
 APOSTART_8_GEN (4886) TCATGTCAACTTTGACACAACGAACAACAAAAATAAATCTTCAGGGCTCATGGATAGTGCGGCAGAGCGTTGGAAGTACCCCTTGTTTATTGGGAAAGGCAGTCGATTGCAGCTACGTGCGTGGTCCAGGTTAC
APOSTART_10_GEN (4830) TCATGTCAACTCTGAAACAACGAACAACAAAAATAAATCTTCAGGGCTCATGGATAGTGCGGCAGAGCGTTGGAAGTACCCCTTGTTTATTGGGAAAGGCAGTCGATTGCAGCTACGTGCGTGGTCCAGGTTAC
APOSTART_12_GEN (4840) TCATGTCAACTTTGACACAACGAACAACAAAAATAAATCTTCAGGGCTCATGGATAGTGCGGCAGAGCGTTGGAAGTACCCCTTGTTTATTGGGAAAGGCGGTTGATTGCAGCTACGTGCGTGGTCCGGGTTAC
```

Section 39

```
                   (5093) 5093  5100      5110      5120      5130      5140      5150      5160      5170      5180      5190      5200      5210         5226
       APOSTART 1 (1900) TTGGAAGT------------------------------------------------------------------------------------------------------------------------------
       APOSTART 2 (1900) TTAGAAGT------------------------------------------------------------------------------------------------------------------------------
       APOSTART_3 (1900) TTGGAAGT------------------------------------------------------------------------------------------------------------------------------
       APOSTART_4 (1903) TTGGAAGT------------------------------------------------------------------------------------------------------------------------------
       APOSTART_5 (1900) TTGGAAGT------------------------------------------------------------------------------------------------------------------------------
       APOSTART_6 (1900) TTGGAAGT------------------------------------------------------------------------------------------------------------------------------
       APOSTART 7 (1903) TTGGAAGT------------------------------------------------------------------------------------------------------------------------------
       APOSTART_8 (1876) TTGGAAGT------------------------------------------------------------------------------------------------------------------------------
       APOSTART_9 (1900) TTGGAAGT------------------------------------------------------------------------------------------------------------------------------
      APOSTART_10 (1885) TTGGAAGT------------------------------------------------------------------------------------------------------------------------------
      APOSTART_11 (1897) TTGGAAGT------------------------------------------------------------------------------------------------------------------------------
      APOSTART_12 (1903) TTGGAAGT------------------------------------------------------------------------------------------------------------------------------
      APOSTART_14 (1873) TTGGAAGT------------------------------------------------------------------------------------------------------------------------------
      APOSTART_15 (1969) TTGGAAGT------------------------------------------------------------------------------------------------------------------------------
      APOSTART_13 (1873) TTGGAAGT------------------------------------------------------------------------------------------------------------------------------
   APOSTART_1_GEN (4968) TTGGAAGTAAGTCATGCA-TCCGTTTTCCATCTACTGGCGA-------AATATCA----TAGAGTACTAGC---AAAATTTTATCATCGTTATTATTTCCATTTTTAACATGGAATGCATCCTGATCGGTGTTT
   APOSTART_2_GEN (4934) TTAGAAGTAAGTCATGCA-TCCGTTTTCCATCTACTGGCGA-------AATATCA----TAGAGTACTAGC---AACATTTTATCATCGTTATTATTTCCATTTTTAACATGGAATGCATCCTGATCGGTGTTT
   APOSTART_5_GEN (4561) TTGGAAGTAAGTCATATACTTTTTTTCCATCTCATGTTCACAACATAAAAATGGTCAGTAGAGTTATAGTCCAGACACTGAGTACTCATTATTATTTCCATTTTAAACAT-GAATGCACTTTGATTGATATTT
   APOSTART_6_GEN (4542) TTGGAAGTAAGTCATTTA-GTTTTTTTCCATCTTGTATGCA-------------CA-----------A-------------TATAAAAATGGTGAGTAGAATTATGGAAAATGAATGCATTTTGAGTGGTATTT
   APOSTART_7_GEN (5008) TTGGAAGTAAGTCGTGCA-TCCGTTTTCCATCTACTGGCGA-------AATATCATGCATAGAGTACTAGC---GAAATTTTATGGTCGTTGTTATTTCCATTTTTAACATGGAATGCATCCTGATCGGTGTTT
   APOSTART_8_GEN (5020) TTGGAAGTAAGTCGTGCA-TCCGTTTTCCATCTACTGGCGA-------AATATCATGCATAGAGTACTAGC---GAAATTTTATGGTCGTTGTTATTTCCATTTTTAACATGGAATGCATCCTGATCGGTGTTT
  APOSTART_10_GEN (4964) TTGGAAGTAAGTCATGCA-TCCGTTTTCCATCTACTGGCGA-------AATATCA----TAGAGTACTAGC---AAAATTTTATCATCGTTATTATTTCCATTTTTAACATGGAATGCATCCCGATCGGTGTTT
  APOSTART_12_GEN (4974) TTGGAAGTAAGTCGTGCA-TCCGTTTTCCATCTACTGGCGA-------AATATCATGCATAGAGTACTAGC---GAAATTTTATGGTCGTTGTTATTTCCATTTTTAACATGGAATGCATCCTGATCGGTGTTT
```

Section 40

```
                   (5227) 5227           5240      5250      5260      5270      5280      5290      5300      5310      5320      5330      5340      5350      5360
       APOSTART 1 (1908) ---------GGATGTTGACATCGGTTCTTCGGCGGTGGCCAATGGGGTTTTGGGCCTGGTGTTTGGTGTTGTCACAACATTGGTAGTTGACATGGCCTTCCTAATACAGG------------------------
       APOSTART 2 (1908) ---------GGATGTTGACATTGGTTCTTCGGCGGTGGCCAATGGGGTTTTGGGCCTGGTGTTTGGTGTTGTCACAACATTGGTAGTTGACATGGCCTTCCTAATACAGG------------------------
       APOSTART_3 (1908) ---------GGATGTTGACATCGGTTCTTCGGCGGTGGCCAATGGGGTTTTGGGCCTGGTGTTTGGTGTTGTCACAACATTGGTAGTTGACATGGCCTTCCTAATACAGG------------------------
       APOSTART_4 (1911) ---------GGATGTTGACATTGGTTCTTCGGCGGTGGCCAATGGGGTTTTGGGCCTGGTGTTTGGTGTTGTCACAACATTGGTAGTTGACATGGCCTTCCTAATACAGG------------------------
       APOSTART_5 (1908) ---------GGATGTTGACATAGGTTCTTCGGCGGTGGCCAACGGCGTTTTGGGCCTGGTGTTTGGTGTTGTCACAACATTGGTAGTTGACATGGCCTTCCTAATACAGG------------------------
       APOSTART_6 (1908) ---------GGATGTTGACATCGGTTCCTCGGCAGTGGCCAACGGGGTTTTGGGCCTGGTGTTTGGTGTTGTTGCAACATTGGTAGTTGACATGGCCTTCCTAATACAGG------------------------
       APOSTART 7 (1911) ---------GGATGTTGACATTGGTTCTTCGGCGGTGGCCAATGGGGTTTTGGGCCTGGTGTTTGGTGTTGTCACAACATTGGTAGTTGACATGGCCTTCCTAATACAGG------------------------
       APOSTART_8 (1884) ---------GGATGTTGACATTGGTTCTTCGGCGGTGGCCAATGGGGTTTTGGGCCTGGTGTTTGGTGTTGTCACAACATTGGTAGTTGACATGGCCTTCCTAATACAGG------------------------
       APOSTART_9 (1908) ---------GGATGTTGACATTGGTTCTTCGGCGGTGGCCAATGGGGTTTTGGGCCTGGTGTTTGGTGTTGTCACAACATTGGTAGTTGACATGGCCTTCCTAATACAGG------------------------
      APOSTART_10 (1893) ---------GGATGTTGACATTGGTTCTTCGGCGGTGGCCAATGGGGTTTTGGGCCTGGTGTTTGGTGTTGTCACAACATTGGTAGTTGACATGGCCTTCCTAATACAGG------------------------
      APOSTART_11 (1905) ---------GGATGTTGACATTGGTTCTTCGGCGGTGGCCAATGGGGTTTTGGGCCTGGTGTTTGGTGTTGTCACAACATTGGTAGTTGACATGGCCTTCCTAATACAGG------------------------
      APOSTART_12 (1911) ---------GGATGTTGACATTGGTTCTTCGGCGGTGGCCAATGGGGTTTTGGGCCTGGTGTTTGGTGTTGTCACAACATTGGTAGTTGACATGGCCTTCCTAATACAGG------------------------
      APOSTART_14 (1881) ---------GGATGTTGACATTGGTTCTTCGGCGGTGGCCAATGGGGTTTTGGGCCTGGTGTTTGGTGTTGTCACAACATTGGTAGTTGACATGGCCTTCCTAATACAGG------------------------
      APOSTART_15 (1977) ---------GGATGTTGACATTGGTTCTTCGGCGGTGGCCAATGGGGTTTTGGGCCTGGTGTTTGGTGTTGTCACAACATTGGTAGTTGACATGGCCTTCCTAATACAGG------------------------
      APOSTART_13 (1881) ---------GGATGTTGACATTGGTTCTTCGGCGGTGGCCAATGGGGTTTTGGGCCTGGTGTTTGGTGTTGTCACAACATTGGTAGTTGACATGGCCTTCCTAATACAGG------------------------
   APOSTART_1_GEN (5087) TCTTCAGGTGGATGTTGACATCGGTTCTTCGGCGGTGGCCAATGGGGTTTTGGGCCTGGTGTTTGGTGTTGTCACAACATTGGTAGTTGACATGGCCTTCCTAATACAGGTAAGAGACCCCAGTGGCTAATGCA
   APOSTART_2_GEN (5053) TCTTCAGGTGGATGTTGACATTGGTTCTTCGGCGGTGGCCAATGGGGTTTTGGGCCTGGTGTTTGGTGTTGTCACAACATTGGTAGTTGACATGGCCTTCCTAATACAGGTAAGAGACCCCAGTGGCTAATGCA
   APOSTART_5_GEN (4694) TCTGCAGGTGGATGTTGACATAGGTTCTTCGGCGGTGGCCAACGGCGTTTTGGGCCTGGTGTTTGGTGTTGTCACAACATTGGTAGTTGACATGGCCTTCCTAATACAGGTAAGGGACCCCAATTGCTATTTGA
   APOSTART_6_GEN (4638) TCTGCAGGTGGATGTTGACATCGGTTCCTCGGCAGTGGCCAACGGGGTTTTGGGCCTGGTGTTTGGTGTTGTTGCAACATTGGTAGTTGACATGGCCTTCCTAATACAGGTAAGGGACCCCAATTGCTATTGCA
   APOSTART_7_GEN (5131) TTTGCAGGTGGATGTTGACATTGGTTCTTCGGCGGTGGCCAATGGGGTTTTGGGCCTGGTGTTTGGTGTTGTCACAACATTGGTAGTTGACATGGCCTTCCTAATACAGGTAAGAGACCCTAGTTGCTAATGCA
   APOSTART_8_GEN (5143) TTTGCAGGTGGATGTTGACATTGGTTCTTCGGCGGTGGCCAATGGGGTTTTGGGCCTGGTGTTTGGTGTTGTCACAACATTGGTAGTTGACATGGCCTTCCTAATACAGGTAAGAGACCCTAGTTGCTAATGCA
  APOSTART_10_GEN (5083) TCTTCAGGTGGATGTTGACATTGGTTCTTCGGCGGTGGCCAATGGGGTTTTGGGCCTGGTGTTTGGTGTTGTCACAACATTGGTAGTTGACATGGCCTTCCTAATACAGGTAAGAGACCCCAGTGGCTAATGCA
  APOSTART_12_GEN (5097) TTTGCAGGTGGATGTTGACATTGGTTCTTCGGCGGTGGCCAATGGGGTTTTGGGCCTGGTGTTTGGTGTTGTCACAACATTGGTAGTTGACATGGCCTTCCTAATACAGGTAAGAGACCCTAGTTGCTAATGCA
```

Section 41

```
                    (5361) 5361     5370      5380      5390      5400      5410      5420      5430      5440      5450      5460      5470      5480     5494
APOSTART 1 (2009)     ----------------------------------------------------------------------------CTAACACGTATGACGAGCTCCCAGAGCAAGTGATCGGTGCAGCCCGGCTGGCCCACGT
APOSTART 2 (2009)     ----------------------------------------------------------------------------CAAACACGTATGATGAGCTCCCAGAGCAAGTGATCGGTGCAGCCCGGCTGGCCCATGT
APOSTART_3 (2009)     ----------------------------------------------------------------------------CTAACACGTATGACGAGCTCCCAGAGCAAGTGATCGGTGCAGCCCGGCTGGCCCACGT
APOSTART_4 (2012)     ----------------------------------------------------------------------------CGAACACGTATGACGAGCTCCCAGAGCAGGTGATCGGTGCAGCCCGGCTGGCCCACGT
APOSTART_5 (2009)     ----------------------------------------------------------------------------CGAACACGTATGACGAGCTCCCAGAGCAGGTGATCGGGGCGGCTCGGCTGGCCCATGT
APOSTART_6 (2009)     ----------------------------------------------------------------------------CGAACACGTATGATGAGCTCCCAGAACAGGTGATCGGTGCGGCCCGATTGGCCCACGT
APOSTART 7 (2012)     ----------------------------------------------------------------------------CGAACACGTATGACGAGCTCCCAGAGCAGGTGATCGGTGCAGCCCGGCTGGCTCACGT
APOSTART_8 (1985)     ----------------------------------------------------------------------------CTAACACGTATGACGAGCTCCCAGAGCAAGTGATCGGTGCAGCCCGGCTGGCCCACGT
APOSTART_9 (2009)     ----------------------------------------------------------------------------CTAACACGTATGACGAGCTCCCAGAGCAAGTGATCGGTGCAGCCCGGCTGGCCCACGT
APOSTART_10 (1994)    ----------------------------------------------------------------------------CTAACACGTATGACGAGCTCCCAGAGCAAGTGATCGGTGCAGCCCGGCTGGCCCACGT
APOSTART_11 (2006)    ----------------------------------------------------------------------------CTAACACGTATGACGAGCTCCCAGAGCAAGTGATCGGTGCAGCCCGGCTGGCCCACGT
APOSTART_12 (2012)    ----------------------------------------------------------------------------CGAACACGTATGACGAGCTCCCAGAGCAGGTGATCGGTGCAGCCCGGCTGGCCCACGT
APOSTART_14 (1982)    ----------------------------------------------------------------------------CGAACACGTATGACGAGCTCCCAGAGCAGGTGATCGGTGCAGCCCGGCTGGCCCACGT
APOSTART_15 (2078)    ----------------------------------------------------------------------------CGAACACGTATGACGAGCTCCCAGAGCAGGTGATCGGTGCAGCCCGGCTGGCCCACGT
APOSTART_13 (1982)    ----------------------------------------------------------------------------CGAACACGTATGACGAGCTCCCAGAGCAGGTGATCGGTGCAGCCCGGCTGGCCCACGT
APOSTART_1_GEN (5221) AAATCCTGAGTTGACTGAAGAAGAGAAAATCTAGTACGGTCTTATCATTGTACGGTTCATTTTATGAACTTGCAGGCTAACACGTATGACGAGCTCCCAGAGCAAGTGATCGGTGCAGCCCGGCTGGCCCACGT
APOSTART_2_GEN (5187) AAATCCTGAGTTGACTGAAGAAGAGAAAATCTAGTACGGTCTTATCATTGTACGGTTCATCTTATGAACTTGCAGGCAAACACGTATGATGAGCTCCCAGAGCAAGTGATCGGTGCAGCCCGGCTGGCCCATGT
APOSTART_5_GEN (4828) GAACCATGAGTAGACTGAAGAAGAGAAAATCTAGTATGGTCTTATCATTGTACGTTTCATATAATGAATTTGCAGGCGAACACGTATGACGAGCTCCCAGAGCAGGTGATCGGGGCGGCTCGGCTGGCCCATGT
APOSTART_6_GEN (4772) AAC-CATGAGTAGACTGAAGAAGATAAA-TCTAGTATGGTCTTATCATTGAACGCTTCATATTCTGAATTTGCAGGCGAACACGTATGATGAGCTCCCAGAACAGGTGATCGGTGCGGCCCGATTGGCCCACGT
APOSTART_7_GEN (5265) AAATCATGAGTTGACTGAAGAAGAGAAAATCTAGTATGGTCTTAGCGTTGTACGGTTCATCTTATGAACTTGTAGGCGAACACGTATGACGAGCTCCCAGAGCAGGTGATCGGTGCAGCCCGGCTGGCTCACGT
APOSTART_8_GEN (5277) AAATCATGAGTTGACTGAAGAAGAGAAAATCTAGTATGGTCTTAGCGTTGTACGGTTCATCTTATGAACTTGTAGGCTAACACGTATGACGAGCTCCCAGAGCAAGTGATCGGTGCAGCCCGGCTGGCCCACGT
APOSTART_10_GEN (5217) AAATCCTGAGTTGACTGAAGAAGAGAAAATCTAGTACGGTCTTATCATTGTACGGTTCATTTTATGAACTTGCAGGCTAACACGTATGACGAGCTCCCAGAGCAAGTGATCGGTGCAGCCCGGCTGGCCCACGT
APOSTART_12_GEN (5231) AAATCATGAGTTGACTGAAGAAGAGAAAATCTAGTATGGTCTTAGCGTTGTACGGTTCATCTTATGAACTTGTAGGCGAACACGTATGACGAGCTCCCAGAGCAGGTGATCGGTGCAGCCCGGCTGGCCCACGT
```

Section 42

```
                    (5495) 5495 5500      5510      5520      5530      5540      5550      5560      5570      5580      5590      5600      5610      5621
APOSTART 1 (2067)     TGAACCGGCGGCAGCGGTAGTACCTGACCTTGACAATAACAGTGATAGTAAAGATAGCAGCAACGATG------ACAACAACAATAACACTTCCTCGGATGACGACTCGTCCAAGAAAACCAACTGA
APOSTART 2 (2067)     TGAACCGGCGGCAGCGGTAGTACCTGACCTTGACAATAACAGTGATAGTAAAGATAGCAGCAACGCTG------ACAACAACAATAACACTTCCTCGGAGGACGACTCGTCCAAGAAAACCAACTGA
APOSTART_3 (2067)     TGAACCGGCGGCAGCGGTAGTACCTGACCTTGACAATAACAGTGATAGTAAAGATAGCAGCAACGATG------ACAACAACAATAACACTTCCTCGGATGACGACTCGTCCAAGAAAACCAACTGA
APOSTART_4 (2070)     TGAACCGGCGGCAGCGGTAGTACCTGACCTTGACAATAACAGTGATAGTAAAGATAGCAGCAACGATG------ACAACAACAATAACACTTCCTCGGAGGACGACTCGTCCAAGAAAACCAACTGA
APOSTART_5 (2067)     CGAACCGGCAGCAGCCGTAGTACCTGATCTTGACAATAACAATGATAGTAAAGATAGCAGCAACGATG------ACAATAACAATAACACTTCCTCGGAGGACGACTCGTCCAAGAAAACCAACTGA
APOSTART_6 (2067)     CGAACCGGCTGCAGCGGTAGTACCTGACCTTGACAATAACAATGATAGTAAAGATATCAGCAACGATGACAATAACAATAACAATAACACTTCCTCGGAGTACGACTCGTCCAAGAAAACCAACTGA
APOSTART 7 (2070)     TGAACCGGCGGCAGCGGTAGTACCTGACCTTGACAATAACAGTGATAGTAAAGATAGCAGCAACGATG------ACAACAACAATAACACTTCCTCGGAGGACGACTCGTCCAAGAAAACCAACTGA
APOSTART_8 (2043)     TGAACCGGCGGCAGCGGTAGTACCTGACCTTGACAATAACAGTGATAGTAAAGATAGCAGCAACGATG------ACAACAACAATAACACTTCCTCGGAGGACGACTCGTCCAAGAAAACCAACTGA
APOSTART_9 (2067)     TGAACCGGCGGCAGCGGTAGTACCTGACCTTGACAATAACAGTGATAGTAAAGATAGCAGCAACGATG------ACAACAACAATAACACTTCCTCGGAGGACGACTCGTCCAAGAAAACCAACTGA
APOSTART_10 (2052)    TGAACCGGCGGCAGCGGTAGTACCTGACCTTGACAATAACAGTGATAGTAAAGATAGCAGCAACGATG------ACAACAACAATAACACTTCCTCGGACGACGACTCGTCCAAGAAAACCAACTGA
APOSTART_11 (2064)    TGAACCGGCGGCAGCGGTAGTACCTGACCTTGACAATAACAGTGATAGTAAAGATAGCAGCAACGATG------ACAACAACAATAACACTTCCTCGGAGGACGACTCGTCCAAGAAAACCAACTGA
APOSTART_12 (2070)    TGAACCGGCGGCAGCGGTAGTACCTGACCTTGACAATAACAGTGATAGTAAAGATAGCAGCAACGATG------ACAACAACAATAACACTTCCTCGGAGGACGACTCGTCCAAGAAAACCAACTGA
APOSTART_14 (2040)    TGAACCGGCGGCAGCGGTAGTACCTGACCTTGACAATAACAGTGATAGTAAAGA-AGCAGCAACGAT-------ACAACAACAATAACACTTCCTCGGAGGACGACTCGTCCAAGAAAACCAACTGA
APOSTART_15 (2136)    TGAACCGGCGGCAGCGGTAGTACCTGACCTTGACAATAACAGTGATAGTAAAGATAGCAGCAACGATG------ACAACAACAATAACACTTCCTCGAAGGACGACTCGTCCAAGAAAACCAACTGA
APOSTART_13 (2040)    TGAACCGGCGGCAGCGGTAGTACCTGACCTTGACAATAACAGTGATAGTAAAGATAGCAGCAACGATG------ACAACAACAATAACACTTCCTCGGAGGACGACTCGTCCAAGAAAACCAACTGA
APOSTART_1_GEN (5355) TGAACCGGCGGCAGCGGTAGTACCTGACCTTGACAATAACAGTGATAGTAAAGATAGCAGCAACGATG------ACAACAACAATAACACTTCCTCGGATGACGACTCGTCCAAGAAAACCAACTGA
APOSTART_2_GEN (5321) TGAACCGGCGGCAGCGGTAGTACCTGACCTTGACAATAACAGTGATAGTAAAGATAGCAGCAACGCTG------ACAACAACAATAACACTTCCTCGGAGGACGACTCGTCCAAGAAAACCAACTGA
APOSTART_5_GEN (4962) CGAACCGGCAGCAGCCGTAGTACCTGATCTTGACAATAACAATGATAGTAAAGATAGCAGCAACGATG------ACAATAACAATAACACTTCCTCGGAGGACGACTCGTCCAAGAAAACCAACTGA
APOSTART_6_GEN (4904) CGAACCGGCTGCAGCGGTAGTACCTGACCTTGACAATAACAATGATAGTAAAGATATCAGCAACGATGACAATAACAATAACAATAACACTTCCTCGGAGTACGACTCGTCCAAGAAAACCAACTGA
APOSTART_7_GEN (5399) TGAACCGGCGGCAGCGGTAGTACCTGACCTTGACAATAACAGTGATAGTAAAGATAGCAGCAACGATG------ACAACAACAATAACACTTCCTCGGAGGACGACTCGTCCAAGAAAACCAACTGA
APOSTART_8_GEN (5411) TGAACCGGCGGCAGCGGTAGTACCTGACCTTGACAATAACAGTGATAGTAAAGATAGCAGCAACGATG------ACAACAACAATAACACTTCCTCGGAGGACGACTCGTCCAAGAAAACCAACTGA
APOSTART_10_GEN (5351) TGAACCGGCGGCAGCGGTAGTACCTGACCTTGACAATAACAGTGATAGTAAAGATAGCAGCAACGATG------ACAACAACAATAACACTTCCTCGGACGACGACTCGTCCAAGAAAACCAACTGA
APOSTART_12_GEN (5365) TGAACCGGCGGCAGCGGTAGTACCTGACCTTGACAATAACAGTGATAGTAAAGATAGCAGCAACGATG------ACAACAACAATAACACTTCCTCGGAGGACGACTCGTCCAAGAAAACCAACTGA
```
