# Supplementary material for: The Role of APOSTART in Switching between Sexuality and Apomixis in Poa pratensis
Source: Genes (Basel). 2020 Aug 14;11(8):941. doi: 10.3390/genes11080941 (PMC7464379; doi:10.3390/genes11080941)
Supplement: Supplementary file 1 [file genes-11-00941-s001.zip › Supplementary Tables/TableS1.docx]

**Table S1.** Information on germplasm source mode of reproduction, number of genotypes analyses and results of the APO-SCAR testing of 63 *P. pratensis* genotypes, 1 *Poa* *arachnifera* genotype and 44 *Poa* *arachnifera* x *Poa pratensis* hybrids.

| **Plant**  **code** | **Germplasm origin** | **Genotypes**  **analyzed** | **Mode of reproduction** | **APO- SCAR amplicon** |
| --- | --- | --- | --- | --- |
| RS7 | Naturalized *P. pratensis* clone  collected, Rionero Sannitico (IS), IT [1] | 1 | apomictic | + |
| S1/1 | *P. pratensis* sexual genotype selected from a Dutch cultivar [2] | 1 | sexual | - |
| L4 | Naturalized *P. pratensis* clone  collected, Colfiorito (PG), IT [3] | 1 | apomictic | na |
| APO | F_1_ segreg population obtained from a cross between L4 and RS7 and characterized for its mode of reproduction [3] | 4 | 2 sexual  1 apomictic  1 parthenogenetic | na |
| PG-F_1_ | F_1_ segreg population obtained from a cross between S1/1 and RS7 and characterized for its mode of reproduction [4,5] | 3 (qRT-PCR)  48 (APO-SCAR) | 20 apomictic  28 sexual | + (20)  - (28) |
| Baron | *P. pratensis* cv. Barenbrug Seeds, Albany, OR, USA | 1 | apomictic | + |
| Barethia | *P. pratensis* cv. Barenbrug Seeds, Albany, OR, USA | 1 | apomictic | + |
| Barrister | *P. pratensis* cv. Barenbrug Seeds, Albany, OR, USA | 1 | apomictic | + |
| KB1 | Naturalized *P. pratensis* clone  collected, USDA-ARS, El Reno, OK, USA | 1 | apomictic | + |
| KB3 | Naturalized *P. pratensis* clone  collected, USDA-ARS, El Reno, OK, USA | 1 | apomictic | + |
| KB5 | Naturalized *P. pratensis* clone  collected, USDA-ARS, El Reno, OK, USA | 1 | sexual | - |
| KB6 | Naturalized *P. pratensis* clone  collected, USDA-ARS, El Reno, OK, USA | 1 | apomictic | + |
| Alene | *P. pratensis* cultivar, Summit Seed, Inc., Manteno, IL, USA | 1 | apomictic | + |
| Cache | *P. pratensis* cultivar, Summit Seed, Inc., Manteno, IL, USA | 1 | apomictic | + |
| KenBlue | *P. pratensis* cultivar, Summit Seed, Inc., Manteno, IL, USA | 1 | apomictic | + |
| S55 | *P. pratensis* experimental forage cultivar, Barenbrug Seeds, Albany, OR, USA | 1 | apomictic | + |
| 9601 | *P. pratensis*, experimental cultivar,  Barenbrug Seeds, Albany, OR, USA | 1 | apomictic | + |
| 1915 | *P. pratensis*, experimental cultivar,  Barenbrug Seeds, Albany, OR, USA | 1 | apomictic | + |
| Pa1FM | *Sexual P. arachnifera genotype used as maternal plant,* USDA-ARS, El Reno, OK, USA | 1 | sexual | - |
| PaPp | F1 population from a  *P. aracnifera* x *P. pratensis* cross (USDA-ARS, El Reno, OK, USA) | 43 | 7 apomictic  36 sexual | + (7)  - (36) |
| Reveille | *P. aracnifera* x *P. pratensis* cv. Gardner Turfgras, Inc., Westminster, CO, USA | 1 | apomictic | + |

**References**

1. Mazzucato, A.; Barcaccia, G.; Pezzotti, M.; Falcinelli, M. Biochemical and molecular markers for investigating the mode of reproduction in the facultative apomict *Poa pratensis L*. *Sexual Plant Reprod* **1995**, *8*, 133–138
2. Matzk, F. New efforts to overcome apomixis in *Poa pratensis L*. *Euphytica*, **1991,** *55***,** 65–72
3. Raggi, L. Mappaggio genetico e mappatura fisica dei geni candidati al controllo dell’apomissia in *Poa pratensis L*. *PhD Thesis*, University of Perugia, Italy. **2007**
4. Albertini, E.; Porceddu, A.; Ferranti, F.; Reale, L.; Barcaccia, G.; Romano, B.; Falcinelli, M. Apospory and parthenogenesis may be uncoupled in *Poa pratensis*: A cytological investigation. *Sex. Plant Reprod.* **2001**, *14*, 213–217
5. Albertini, E.; Porceddu, A.; Ferranti, F.; Reale, L.; Barcaccia, G.; Romano, B.; Falcinelli, M. Apospory and parthenogenesis may be uncoupled in *Poa pratensis*: A cytological investigation. *Sex. Plant Reprod.* **2001**, *14*, 213–217.
