# Supplementary material for: The Role of APOSTART in Switching between Sexuality and Apomixis in Poa pratensis
Source: Genes (Basel). 2020 Aug 14;11(8):941. doi: 10.3390/genes11080941 (PMC7464379; doi:10.3390/genes11080941)
Supplement: Supplementary file 1 [file genes-11-00941-s001.zip › Supplementary Tables/TableS2.docx]

**Table S2.** Sequence of primers used (rpoC2/rps2 as in Raggi et al. [1]).

| **Name** |  | **Sequence 5’-> 3’** |
| --- | --- | --- |
| APOSTART_5 | For | CAATGCCGAAAAACCAGAAG |
|  | Rev | GCTTAGCATCGTTGTCAGCTTTAC |
| APOSTART_6 | For | TGGACAGTACCGGATAGCACG |
|  | Rev | AACGTTGCAGCAATGATCCC |
| APOSTART_7 | For | TTGACCTGAGAGGATGGTTCCT |
|  | Rev | CCATGTGCTGGTTTAGTCTTGG |
| APOSTART_8 | For | CTGTGTTGCTGATGAATGCC |
|  | Rev | CTCCATTTCATGTAGCTTCTGG |
| APOSTART_10 | For | GCTACTTCGATGCCAAAATGAA |
|  | Rev | CTGCCATAGCGAAAGCTACAGTC |
| APOSTART-12 | For | GGAACTTGGAGGGCAGTTCTT |
|  | Rev | ACCGATATCAGGCTCATTGGTC |
| Ppβ-tubulin | For | GTGGAGTGGATCCCCAACAA |
|  | Rev | AAAGCCTTCCTCCTGAACATGG |
| APO- SCAR | For | AGTTTTTTTCCATCTTGTATG |
|  | Rev | CCTGTATTAGGAAGGCCATGTCAACTACC |
| rpoC2/rps2 | For | TTATTTATTTCAAGCTATTTCGG |
|  | Rev | AATATCTTCTTGTCATTTTTTCC |

References

1. Raggi, L.; Bitocchi, E.; Russi, L.; Marconi, G.; Sharbel, T.F.; Veronesi, F.; Albertini, E. Understanding genetic diversity and population structure of a Poa pratensis worldwide collection through morphological, nuclear and chloroplast diversity analysis. *PLoS ONE* **2015**, *10*, doi:10.1371/journal.pone.0124709.
