# Supplementary material for: The Role of APOSTART in Switching between Sexuality and Apomixis in Poa pratensis
Source: Genes (Basel). 2020 Aug 14;11(8):941. doi: 10.3390/genes11080941 (PMC7464379; doi:10.3390/genes11080941)
Supplement: Supplementary file 1 [file genes-11-00941-s001.zip › Supplementary Tables/TableS3.docx]

**Table 3.** List of APOSTART cDNA and genomic clones with relative NCBI accession numbers.

| **Name** | **CDNA accession number** | **Genomic DNA accession number** |
| --- | --- | --- |
| APOSTART_1 | AJ786392 | AJ786392 |
| APOSTART_2 | AJ786393 | AJ786393 |
| APOSTART_3 | MK135894 | - |
| APOSTART_4 | MK135895 | - |
| APOSTART_5 | MK135896 | MT327123 |
| APOSTART_6 | MK135897 | MT327124 |
| APOSTART_7 | MK135898 | MT327125 |
| APOSTART_8 | MK135899 | MT327126 |
| APOSTART_9 | MK135900 | - |
| APOSTART_10 | MK135901 | MT327127 |
| APOSTART_11 | MK135902 | - |
| APOSTART_12 | MK135903 | MT327128 |
| APOSTART_13 | MK135904 | - |
| APOSTART_14 | MK135905 | - |
| APOSTART_15 | MK135906 | - |
