# Supplementary material for: The Role of APOSTART in Switching between Sexuality and Apomixis in Poa pratensis
Source: Genes (Basel). 2020 Aug 14;11(8):941. doi: 10.3390/genes11080941 (PMC7464379; doi:10.3390/genes11080941)
Supplement: Supplementary file 1 [file genes-11-00941-s001.zip › Supplementary Tables/TableS7.docx]

**Table 7.** APOSTART protein secondary structures predictions from analyzed genes. Α α-helices, β-sheets and coils are represented in percentages as well as the solvent access and were calculated with PredictProtein algorithms. Disordered regions were predicted with RaptorX. H (α-helix; %); E (β-sheet; %); C (Coil; %); E (Exposed; %); M (Medium; %); B (Buried; %).

| **Gene** | **Secondary structure** | | | **Solvent access** | | | **Disordered**  **regions** | |
| --- | --- | --- | --- | --- | --- | --- | --- | --- |
|  | **H** | **E** | **C** | **E** | **M** | **B** | **N. of basis** | **%** |
| *P. pratensis* APOSTART_1 | 12 | 28 | 58 | 48 | 27 | 23 | 192 | 26 |
| *P. pratensis* APOSTART_2 | 13 | 28 | 58 | 48 | 27 | 23 | 189 | 26 |
| *P. pratensis* APOSTART_3 | 12 | 28 | 58 | 48 | 27 | 23 | 192 | 26 |
| *P. pratensis* APOSTART_4 | 13 | 28 | 57 | 48 | 26 | 24 | 194 | 27 |
| *P. pratensis* APOSTART_5 | 12 | 28 | 58 | 48 | 27 | 23 | 196 | 27 |
| *P. pratensis* APOSTART_6 | 13 | 28 | 58 | 49 | 27 | 23 | 193 | 26 |
| *P. pratensis* APOSTART_7 | 12 | 29 | 58 | 49 | 27 | 23 | 193 | 26 |
| *P. pratensis* APOSTART_8 | 11 | 29 | 59 | 48 | 26 | 24 | 189 | 26 |
| *P. pratensis* APOSTART_9 | 12 | 29 | 57 | 47 | 27 | 25 | 190 | 26 |
| *P. pratensis* APOSTART_10 | 12 | 27 | 59 | 49 | 26 | 23 | 190 | 26 |
| *P. pratensis* APOSTART_11 | 12 | 30 | 57 | 47 | 27 | 24 | 187 | 26 |
| *P. pratensis* APOSTART_12 | 12 | 29 | 57 | 49 | 26 | 24 | 192 | 26 |
| *P. pratensis* APOSTART_13 | 11 | 29 | 58 | 48 | 27 | 24 | 185 | 26 |
| *P. pratensis* APOSTART_14 | 11 | 29 | 58 | 47 | 27 | 24 | 187 | 26 |
| *P. pratensis* APOSTART_15 | 12 | 27 | 59 | 49 | 26 | 23 | 213 | 28 |
