# Supplementary figures and images for: The Role of APOSTART in Switching between Sexuality and Apomixis in Poa pratensis
Source: Genes (Basel). 2020 Aug 14;11(8):941. doi: 10.3390/genes11080941 (PMC7464379; doi:10.3390/genes11080941)

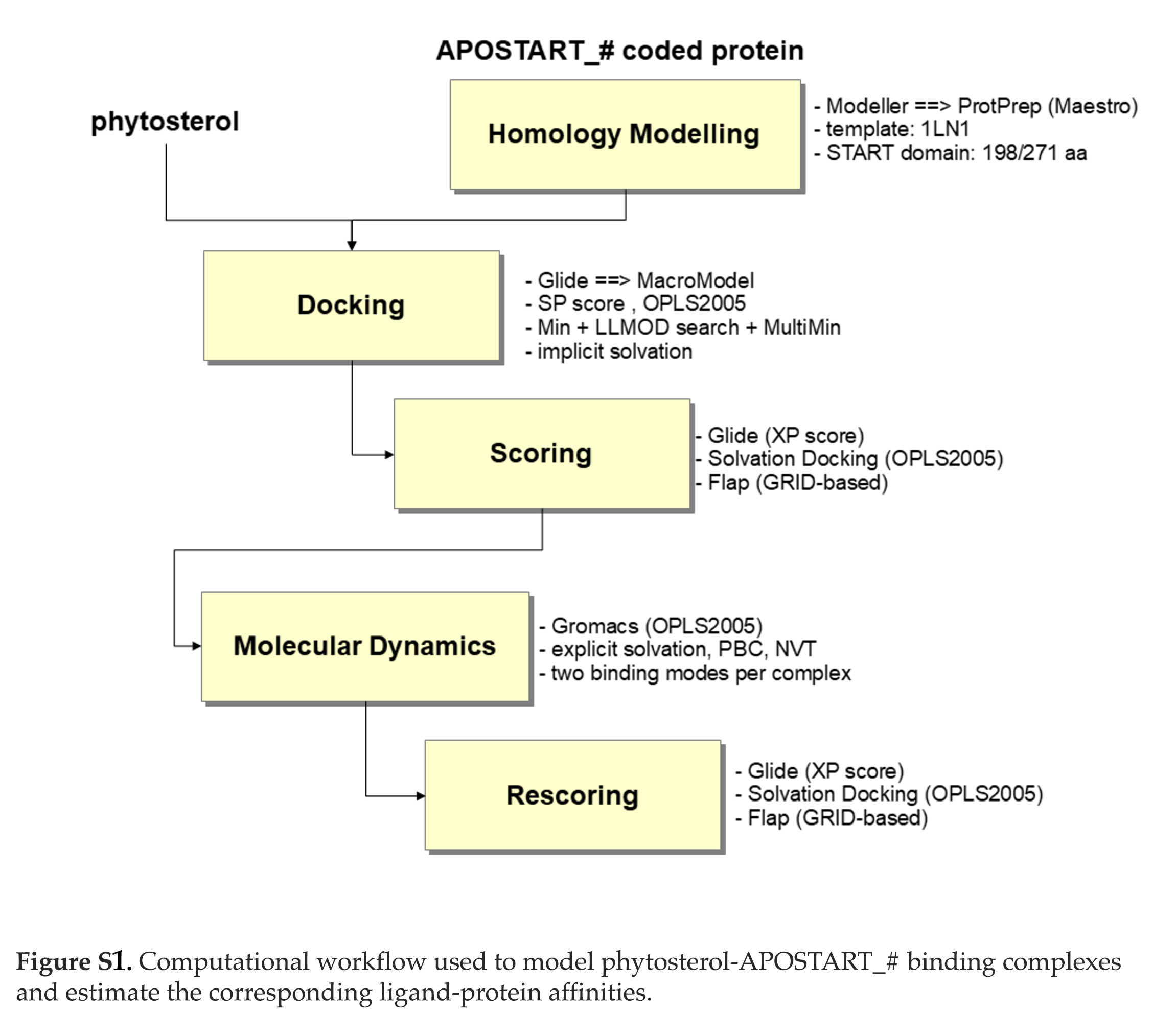

Supplement: Supplementary file 1 [file genes-11-00941-s001.zip › Supplementary Figures/Figure S1.tif]

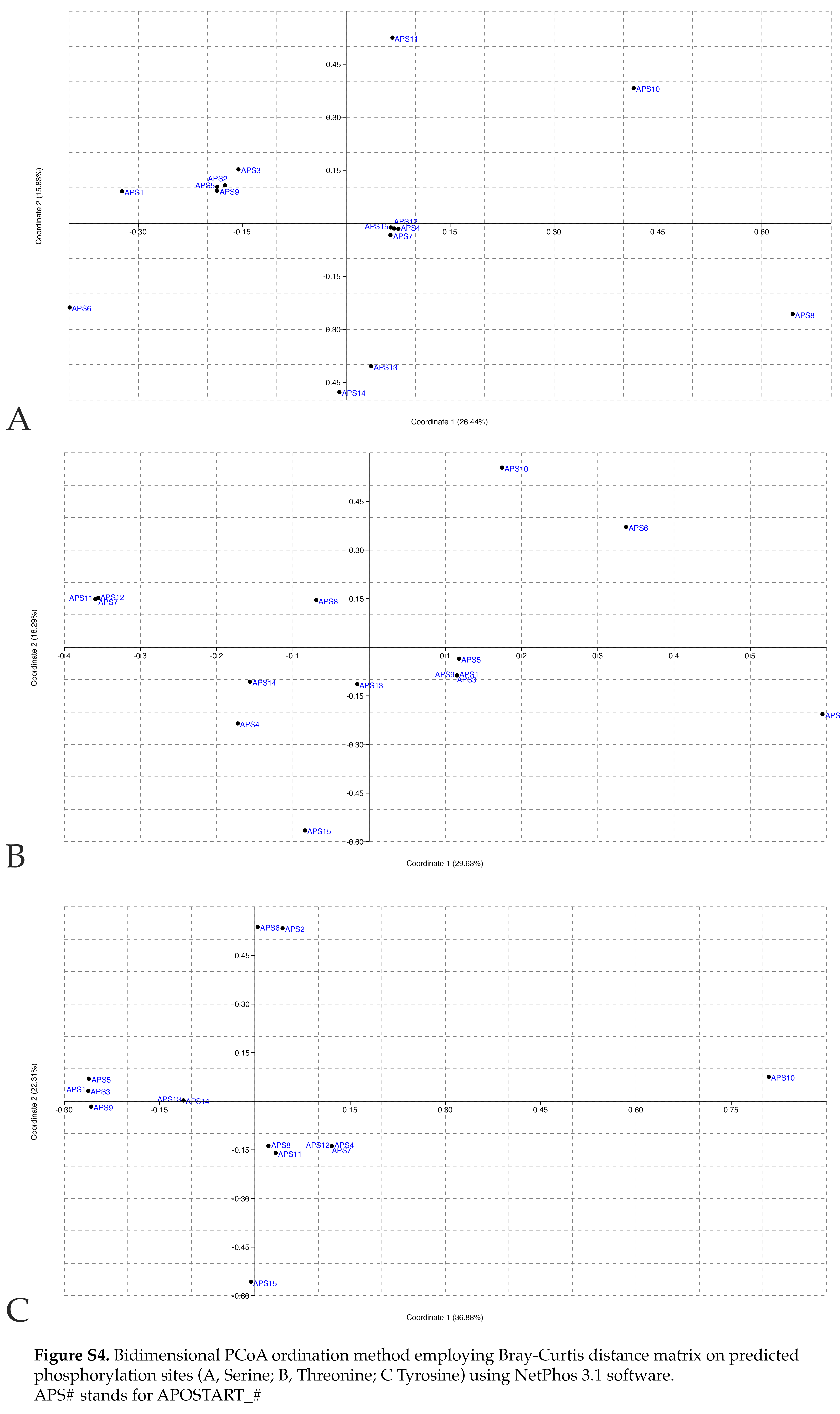

Supplement: Supplementary file 1 [file genes-11-00941-s001.zip › Supplementary Figures/Figure S4.tif]

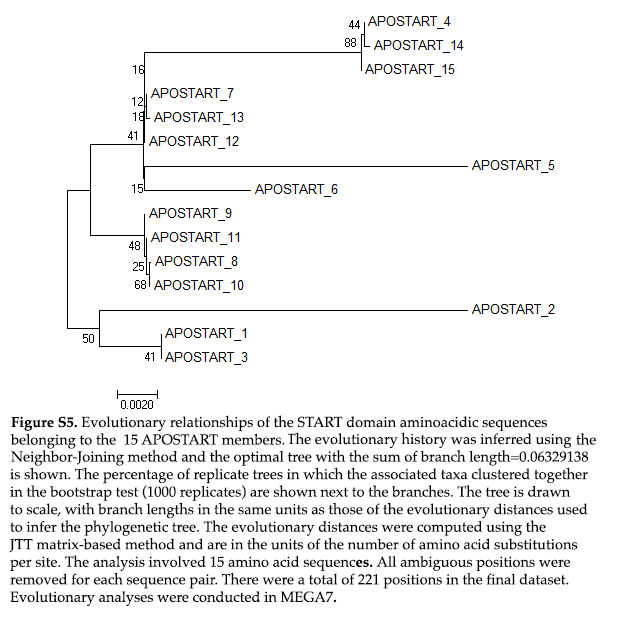

Supplement: Supplementary file 1 [file genes-11-00941-s001.zip › Supplementary Figures/Figure S5.tif]

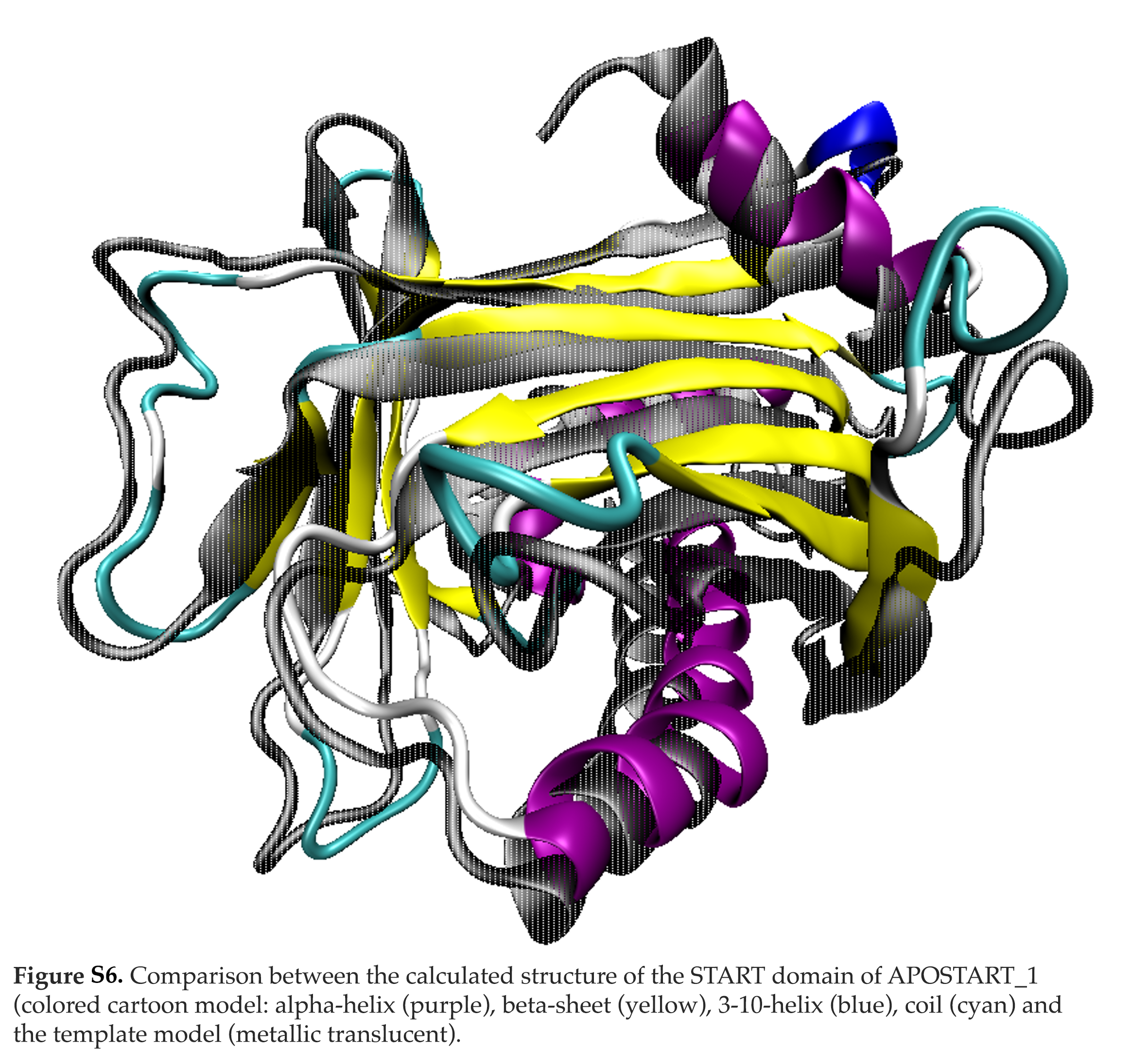

Supplement: Supplementary file 1 [file genes-11-00941-s001.zip › Supplementary Figures/Figure S6.tif]

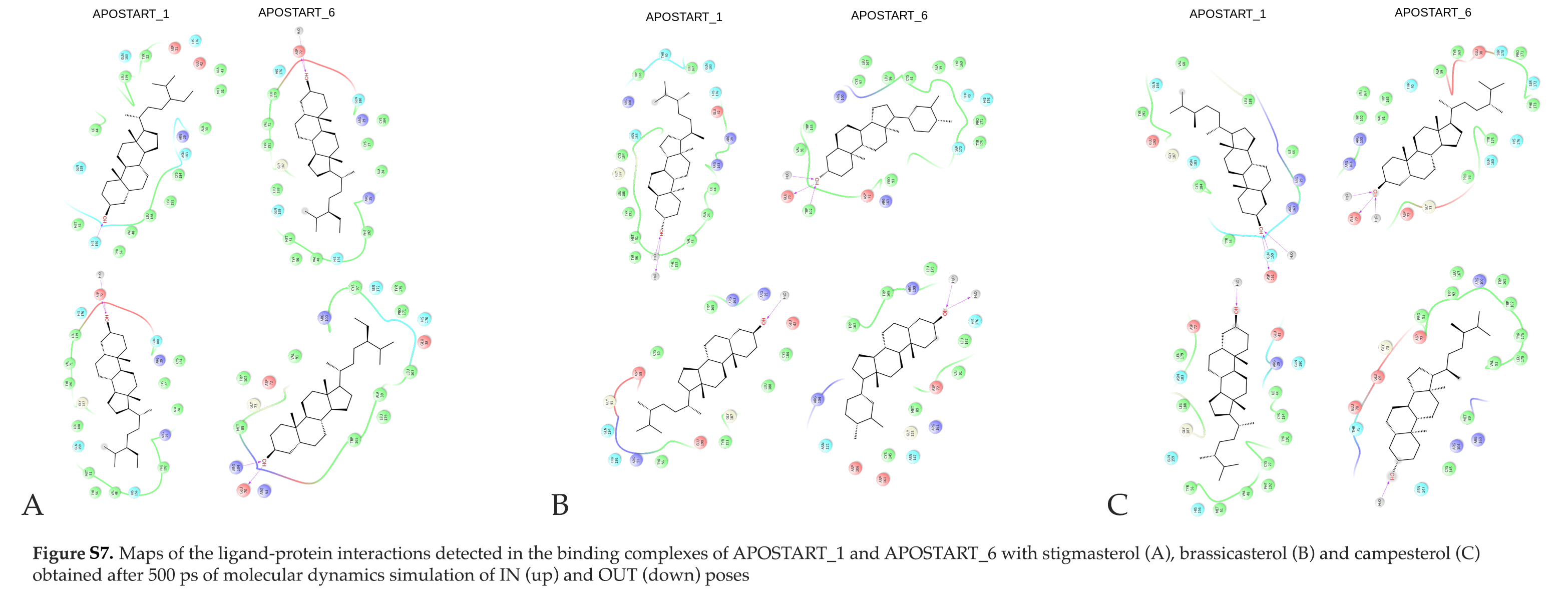

Supplement: Supplementary file 1 [file genes-11-00941-s001.zip › Supplementary Figures/Figure S7.tif]
